# Is the co-option of jasmonate signalling for botanical carnivory a universal trait for all carnivorous plants?

**DOI:** 10.1093/jxb/erad359

**Published:** 2023-09-14

**Authors:** Andrej Pavlovič, Jana Koller, Ondřej Vrobel, Ivo Chamrád, René Lenobel, Petr Tarkowski

**Affiliations:** Department of Biophysics, Faculty of Science, Palacký University, Šlechtitelů 27, CZ-783 71, Olomouc, Czech Republic; Department of Biophysics, Faculty of Science, Palacký University, Šlechtitelů 27, CZ-783 71, Olomouc, Czech Republic; Czech Advanced Technology and Research Institute, Palacký University, Šlechtitelů 27, CZ-783 71, Olomouc, Czech Republic; Center of the Region Haná for Biotechnological and Agricultural Research, Department of Genetic Resources for Vegetables, Medicinal and Special Plants, Crop Research Institute, Šlechtitelů 29, CZ-783 71 Olomouc, Czech Republic; Laboratory of Growth Regulators, Faculty of Science, Palacký University and Institute of Experimental Botany of the Czech Academy of Sciences, Šlechtitelů 27, CZ-783 71, Olomouc, Czech Republic; Laboratory of Growth Regulators, Faculty of Science, Palacký University and Institute of Experimental Botany of the Czech Academy of Sciences, Šlechtitelů 27, CZ-783 71, Olomouc, Czech Republic; Czech Advanced Technology and Research Institute, Palacký University, Šlechtitelů 27, CZ-783 71, Olomouc, Czech Republic; Center of the Region Haná for Biotechnological and Agricultural Research, Department of Genetic Resources for Vegetables, Medicinal and Special Plants, Crop Research Institute, Šlechtitelů 29, CZ-783 71 Olomouc, Czech Republic; University of Ghent, Belgium

**Keywords:** Aspartic protease, carnivorous plant, digestive enzymes, electrical signal, plant defence, jasmonic acid, phytohormone, wounding

## Abstract

The carnivorous plants in the order *Caryophyllales* co-opted jasmonate signalling from plant defence to botanical carnivory. However, carnivorous plants have at least 11 independent origins, and here we ask whether jasmonate signalling has been co-opted repeatedly in different evolutionary lineages. We experimentally wounded and fed the carnivorous plants *Sarracenia purpurea* (order *Ericales*), *Cephalotus follicularis* (order *Oxalidales*), *Drosophyllum lusitanicum* (order *Caryophyllales*), and measured electrical signals, phytohormone tissue level, and digestive enzymes activity. Coronatine was added exogenously to confirm the role of jasmonates in the induction of digestive process. Immunodetection of aspartic protease and proteomic analysis of digestive fluid was also performed. We found that prey capture induced accumulation of endogenous jasmonates only in *D. lusitanicum*, in accordance with increased enzyme activity after insect prey or coronatine application. In *C. follicularis*, the enzyme activity was constitutive while in *S. purpurea* was regulated by multiple factors. Several classes of digestive enzymes were identified in the digestive fluid of *D. lusitanicum*. Although carnivorous plants from different evolutionary lineages use the same digestive enzymes, the mechanism of their regulation differs. All investigated genera use jasmonates for their ancient role, defence, but jasmonate signalling has been co-opted for botanical carnivory only in some of them.

## Teaser

Carnivorous plants use jasmonates for plant defence like ordinary plants, but jasmonates were co-opted for botanical carnivory only in some of them.

## Introduction

Jasmonates (JAs) are important regulators in plant responses to biotic and abiotic stresses as well as in development (Wasternack and Hause, 2013). Mechanical wounding or herbivore attack is one of the most prominent examples where JAs are involved as a signal (Koo *et al*., 2009). The endogenous rise of JAs upon wounding or herbivore and pathogen attack is associated with the induction of synthesis of secondary metabolites and activation of the defence response (Wasternack and Hause, 2013). Local wounding by herbivores leads to a burst in newly synthesized JAs not only in local leaves but also in leaves distal to wounds, activating a systemic response. The signals responsible for systemic JA accumulation in distal leaves of the experimental model plant Arabidopsis are electrical signals (Mousavi *et al*., 2013), Ca^2+^ wave (Toyota *et al*., 2018), reactive oxygen species (ROS; Miller *et al*., 2009; Suzuki *et al*., 2013), and/or JAs (Li *et al*. 2020), and the signals co-propagate together (Gilroy *et al*., 2014). All leaves that receive the signals accumulate JAs within a few minutes. In the nucleus, the bioactive isoleucine conjugate of JA (JA-Ile) triggers an interaction between the CORONATINE INSENSITIVE1 (COI1) receptor and members of the JASMONATE ZIM-DOMAIN (JAZ) family of repressors. COI1-mediated degradation of JAZ repressors activates the reprogramming of gene expression leading to the plant defence response (Chini *et al*., 2007; Thines *et al*., 2007; Fonseca *et al*., 2009; Sheard *et al*., 2010).

Jasmonate signalling is a very old pathway, present already in *Bryophyta* and first appearing probably in the common ancestor of extant land plants more than 450 million years ago (mya). Already the liverwort *Marchantia polymorpha* contains all the core components of the JA signalling pathway (COI1, JAZ, etc.), but it is not able to synthesize the bioactive ligand JA-Ile known from flowering plants (Monte *et al*., 2018; Chini *et al*., 2023), which probably first appeared in lycophytes (Pratiwi *et al*., 2017). Later the JA signalling pathway was co-opted to regulate fertility in plants (Monte *et al*., 2018). Thus, it can be suggested that the first flowering plants (angiosperms), dated to approx. 194 mya, surely had the complete JA signalling pathway as we know it today from the experimental model plant Arabidopsis.

In nutrient-poor habitats, a special group of so-called carnivorous plants have evolved sophisticated mechanisms to obtain nutrients deficient in the soil from an animal prey. They modified their leaves into traps, which capture an animal prey by five different mechanisms: adhesive (‘flypaper’) traps with a sticky glandular surface; pitfall (‘pitcher’) traps forming a central cavity or small tanks; mobile snap-traps with rapidly closing lobes; suction (‘bladder’) traps actively forming negative pressure inside; and specialized eel (‘lobster-pot’ and ‘cork-screw’) traps formed by screwed, tubular leaves with a narrow cavity lined with retrorse hairs (Adamec *et al*., 2021). The phylogenetically oldest lineage of carnivorous plants is *Caryophyllales*, dating back to 83–95.1 mya (Fleischmann *et al*., 2018), and they surely exploited the JA signalling pathway for defence as we know in non-carnivorous plants (Dávila-Lara *et al*., 2021). Recent studies have shown that all the studied genera of carnivorous plants within the order *Caryophyllales* regardless of trapping mechanisms (adhesive traps in *Drosera*, snap-traps in *Dionaea* and *Aldrovanda*, pitfall traps in *Nepenthes*) accumulate JA and JA-Ile in response to experimental feeding. Moreover, the exogenous application of JA (or its molecular agonist coronatine) induced the carnivorous response, i.e. formation of digestive cavity and/or expression of digestive enzymes and transporters (Nakamura *et al*., 2013; Libiaková *et al*., 2014; Mithöfer *et al*., 2014; Buch *et al*., 2015; Böhm *et al*., 2016a, b; Yilamujiang *et al*., 2016; Krausko *et al*., 2017; Pavlovič *et al*., 2017; Jakšová *et al*., 2020, 2021), confirming the role of endogenous JAs in the regulation of botanical carnivory. Taking into account the phylogenetic age of carnivorous plants and angiosperms, regulation of carnivory by the JAs is therefore not an ancient character, but derived from plant defence more recently in evolution. Thus, carnivorous plants co-opted the pre-existing JA signalling pathway from plant defence to botanical carnivory and turned defence into offense (Pavlovič and Saganová, 2015; Bemm *et al*., 2016; Pavlovič and Mithöfer, 2019). However, carnivorous plants have a polyphyletic origin and have evolved at least 11 times independently in a time span of 95.1–1.9 mya (Fleischmann *et al*., 2018) in six orders, 13 families, and 20 genera (Adamec *et al*., 2021 and updated by the genus *Triantha*, Lin *et al*., 2021). All the studied genera mentioned above belong to the same oldest order: *Caryophyllales*. Only recently, we showed that carnivorous plants from the order *Lamiales* (*Pinguicula*, *Utricularia*) have not co-opted the JA signalling pathway for the regulation of digestive physiology despite using similar enzymes (Kocáb *et al*., 2020; Jakšová *et al*., 2021), indicating that JAs are not ubiquitous signalling molecules in botanical carnivory.

In this study, we investigated the function of JAs in botanical carnivory in three non-related orders of carnivorous plants: *Oxalidales* (represented by *Cephalotus follicularis*), *Ericales* (represented by *Sarracenia purpurea* ssp. *venosa*) and *Caryophyllales* (represented by *Drosophyllum lusitanicum*) to complement the picture of involvement of JAs in botanical carnivory. The tested plants were experimentally fed or wounded and analysed for the phytohormone tissues level and enzyme activities. Coronatine, a molecular mimic of JA-Ile, was exogenously added to confirm or deny the role of JAs in the carnivorous response. The results showed that carnivorous plants have employed multiple mechanisms for regulation of digestive enzyme activity.

## Materials and methods

### Plant material and experimental set-up

**Fig. 1.**
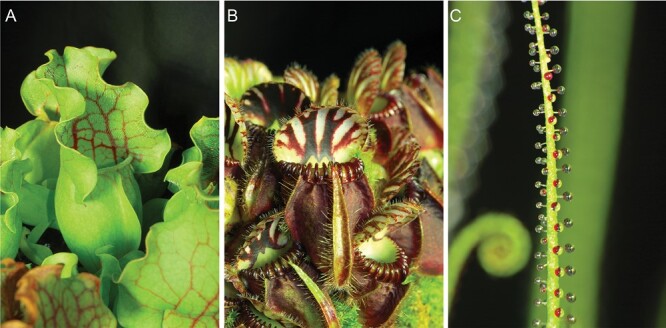
Traps of experimental carnivorous plants. (A) *Sarracenia purpurea* ssp. *venosa*. (B) *Cephalotus follicularis.* (C) *Drosophyllum lusitanicum.*

The carnivorous plants *Sarracenia purpurea* ssp. *venosa* L., *Cephalotus follicularis* Labill., and *Drosophyllum lusitanicum* (L.) Link (Fig. 1), growing under standard greenhouse conditions at Palacký University in Olomouc (Czech Republic), were used in our experiments. Distilled water (6 ml) was added into the 10- to 20-day-old *S. purpurea* ssp*. venosa* pitcher after opening and shortly before feeding because this species is not able to secrete the permanent level of digestive fluid but collects rainfall water. In contrast, *C. follicularis* pitchers are protected by the lid, which prevents rainwater from entering the pitcher and diluting the digestive enzymes inside. The mature fully developed pitchers of *S. purpurea* and *C. follicularis* were fed on two live mealworms (*Tenebrio molitor*). Digestive fluid (0.5 ml) was collected from the mature and fully developed pitchers before feeding (0 h) and then after 24, 48, 96, and 168 h for the enzyme assay and western blotting. Digestive fluid (0.5 ml) was also collected from control non-fed pitchers at the same time points. In a parallel experiment with another group of plants, the lower digestive part of the pitchers, which have contact with digestive fluid, were collected before (0 h) and 2, 24, and 48 (96) h after feeding and immediately frozen in liquid nitrogen for phytohormone analyses. The lower digestive parts of control non-fed pitchers were also collected at the same time points. Because *D. lusitanicum* with its sticky leaves is specialized for smaller prey in comparison with pitcher plants, it was fed on fruit flies (*Drosophila melanogaster*). Each investigated 10–15 cm-long sticky leaf was fed on five live fruit flies. Before (0 h) and 24, 48, and 96 h after feeding three non-fed control and fed leaves were cut off using a scalpel, and the traps were submerged in 4 ml of 50 mM Na-acetate buffer solution (pH 5.0) for 3 min to collect the exudates for enzyme assay, western blotting and LC-MS/MS as described in Krausko *et al*. (2017). In a parallel experiment with another group of plants, four leaves were collected before (0 h) and 2 and 24 h after feeding and frozen in liquid nitrogen for phytohormone analyses.

To investigate the possible role of JAs in enzyme induction, the molecular mimic of JA-Ile, coronatine (Sigma-Aldrich, MO, USA), was added into the pitchers of *S. purpurea* and *C. follicularis* to a final 100 µM concentration. The same volume of distilled water was added to control pitcher to adjust dilution of enzyme in coronatine-treated plants. Digestive fluid (0.5 ml) was collected at the same time points as mentioned above from coronatine-treated and control pitchers. *Drosophyllum lusitanicum* plants were sprayed by water (control) and 100 µM coronatine and the exudate was collected the same way as mentioned above. The potency of 100 µM coronatine was tested on *Drosera capensis* and *Nepenthes* × *Mixta* plants (positive controls), where it induced the strong leaf bending reaction and enzyme activity, respectively.

In wounding experiments (positive control), the pitcher plants *S. purpurea* and *C. follicularis* were wounded 10 times with a needle within 1 min in the lower digestive part of the pitchers and the same pitcher part was collected 2 h after wounding for phytohormone analyses. *Drosophyllum lusitanicum* was wounded five times (because it was fed on five fruit flies in the previous experiment) and the four wounded and control leaves were collected 2 h after wounding for phytohormone analyses.

For testing the developmental control of enzyme secretion in *S. purpurea*, we monitored and waited for the two traps that opened exactly on the same day. Five millilitres of distilled water was added upon its opening in the first trap, 100 µl of fluid was collected immediately, and then every week for 5 weeks. In the second trap, which opened on the same day as the first trap, 5 ml of water was added after 2 weeks. Again, 100 µl of fluid was collected immediately and then every week for 5 weeks.

For testing developmental control of enzyme secretion, we monitored developing pitchers of *C. follicularis*. On the day these pitchers first opened their lids (time point 0), 100 µl of digestive fluid was sampled and then every week for 7 weeks.

### Extracellular recording of electrical signals

The electrical signals were measured using non-invasive, non-polarizable Ag–AgCl surface electrodes (Scanlab Systems, Prague, Czech Republic) moistened with a drop of conductive EV gel (Hellada, Prague, Czech Republic), which is commonly used in electrocardiography, inside a Faraday cage under standard laboratory conditions (room temperature of 23 ± 1 °C and relative air humidity of 60 ± 5%) according to Ilík *et al.* (2010). The electrode was attached to the lower external part of *S. purpurea* and *C. follicularis* pitchers in the area where the pitcher is in contact with digestive fluid inside. After signal stabilization on the pitcher traps for approximately half an hour, two mealworms were added into the pitchers or the pitcher was wounded with a needle at 1–2 cm distance from the recording electrode. In *S. purpurea*, 6 ml of distilled water was added a few minutes before feeding. In *D. lusitanicum*, the electrode was attached to a stalked gland, and electrical signals were monitored in response to a gentle touch as had been described for sundew plants (*Drosera* spp.) before (Williams and Pickard 1972; Krausko *et al*., 2017) or in response to leaf wounding 1–2 cm distant from the recorded stalked gland. The reference electrode was immersed into a basin of water, which was placed below the pot with the measured plant. The electrodes were connected to an amplifier (gain: 1–1000; noise: 2–3 mV; bandwidth (−3 dB): 10^5^ Hz; response time: 10 µs; input impedance: 10^12^ Ω) and the data were collected every 30 ms.

### Quantitative analysis of phytohormones

Endogenous levels of phytohormones were quantified using the isotope dilution LC-MS/MS method (Ljung *et al.*, 2010). The collected plant tissues were frozen in liquid nitrogen and ground using a mortar and pestle. Homogenized material was extracted and purified as described by Šimura *et al.* (2018). Briefly, 25 mg of sample was extracted with 1 ml of ice-cold 50% acetonitrile (ACN) containing a mixture of stable isotope labelled standards. Unlabelled (salicylic acid (SA), JA-Ile, abscisic acid (ABA)) and labelled standards (D4-SA, D2-JA-Ile, D6-ABA) were purchased from OlChemIm (Olomouc, Czech Republic), and JA and D5-JA were purchased from Merck (Germany). The extraction was performed with the assistance of an ice-cold ultrasonic bath for 30 min. After centrifugation (20 000 *g*, 15 min, 4 °C), samples were purified using solid-phase extraction (SPE) with Waters (USA) Oasis HLB columns (30 mg, 1 ml cartridge) activated by 1 ml methanol and equilibrated by 1 ml H_2_O and 1 ml of 50% ACN. During sample loading, the flow-through fraction was collected and pooled with the fraction from a single washing step of 1 ml 30% ACN. Collected fractions were evaporated under vacuum. If necessary, dried samples were stored at −20 °C prior to analysis. For analysis, samples were resuspended in 40 µl of mobile phase, filtered through 0.2 µm microspins (Ciro, USA) and analysed by LC-(+)ESI-MS/MS in multiple reaction monitoring (MRM) mode. LC-MS/MS analyses were performed using a Nexera X2 modular liquid chromatograph system coupled to an MS-8050 triple quadrupole mass spectrometer (Shimadzu, Japan) via an electrospray interface. Chromatographic separation was performed using a reverse-phase analytical column (Waters CSH C18, 2.1 mm × 150 mm, 1.7 µm). The aqueous solvent A consisted of 15 mM formic acid adjusted to pH 3.0 with ammonium hydroxide. Solvent B was pure ACN. Separation was achieved with gradient elution at a flow rate of 0.4 ml min^−1^ at 40 °C. A linear gradient consisted of two parts (0–1 min 20% B; 1–11 min 80% B) followed by washing and equilibration to initial conditions for a further 7 min. If possible, up to three MRM transitions (one quantitative, the others qualitative; Supplementary Table S1) were monitored for each analyte to ensure as much confidence as possible in the correct identification of analytes in the different plant matrices. Raw data were processed using Shimadzu software LabSolutions v. 5.97 SP1. All data were log-transformed to calculate the results. To reduce experimental bias, the procedure included a randomized sample list and blinding was imposed on the analyst (OV).

### Proteomic characterization of digestive fluid

Because the composition of digestive fluid was analysed previously in *S. purpurea* and *C. follicularis* (Fukushima *et al*., 2017), we focused our analysis on the digestive fluid of *D. lusitanicum*. The protein composition of *Drosophyllum* digestive fluid was characterized following the approach by Kocáb *et al.* (2020) with several modifications. Briefly, 100-µl aliquots of the collected digestive fluids from control, fed, and coronatine-treated plants in 50 mM Na-acetate buffer solution (pH 5.0) were mixed with Laemmli sample buffer in a ratio of 1:1 (v/v) and separated on 12% polyacrylamide gel by SDS-PAGE (Laemmli, 1970). The resolved proteins were stained with colloidal Coomassie (Candiano *et al.*, 2004) and digested *in gel* as described previously (Shevchenko *et al.*, 2006). The resultant peptides were extracted, cleaned using C18 StageTips (Rappsilber *et al.*, 2008) and analysed by LC-ESI-MS/MS with all of the specific settings identical to that published earlier (Chamrád *et al.*, 2014). The acquired MS data were processed with DataAnalysis v4.3 (x64, Bruker Daltonics) and exported as corresponding mgf files. The mgf files were uploaded into the software Peaks X Pro (Bioinformatic solutions Inc., ON, Canada; Tran *et al.*, 2018) and searched against a database containing proteins related to *Caryophyllales* downloaded from the NCBI repository (285 389 sequences; downloaded on 12.8.2022). The searches were performed with Denovo, PEAKS, PEAKS PTM, and SPIDER options with the following settings: enzyme, trypsin; digest mode, semispecific; fixed modification, carbamidomethylation; variable modification, acetylation (protein N-term), oxidation of methionine, and deamidation of glutamine and asparagine. The mass error tolerances for MS and MS/MS were set at 50 ppm and 0.05 Da, respectively. The following parameters were applied for filtering the final SPIDER search results: FDR limits for peptide-spectrum matches and proteins, 1% and 5%, respectively; a requirement of at least one assigned unique peptide. A pBLAST search was performed against UniProtKB/Swiss-Prot database with green plants (taxid:33090) set as the organism of choice to assign the missing protein sequence annotations. The BLAST hits were subsequently filtered, and the lowest e-value, together with the highest bit score and percentage positives, was required for valid homolog identification.

### Enzyme assay

Proteolytic activity of digestive fluid was determined by incubating 150 μl of digestive fluid with 150 μl of 2% (w/v) bovine serum albumin (BSA) in 200 mM glycine–HCl (pH 3.0) at 37 °C for 1 h (*D. lusitanicum*) or 2 h (*C. follicularis*, *S. purpurea*). The reaction was stopped by the addition of 450 μl of 5% (w/v) trichloroacetic acid (TCA). Samples were incubated on ice for 10 min and centrifuged at 20 000 *g* for 10 min at 4 °C. The amount of released non-TCA-precipitable peptides was used as a measure of proteolytic activity, which was determined by comparing the absorbance of the supernatant at 280 nm with that of a blank sample with a Specord 250 Plus double-beam spectrophotometer (Analytik Jena, Germany). One unit of proteolytic activity is defined as an increase of 0.001 min^–1^ in the absorbance at 280 nm (Matušíková *et al*., 2005).

To measure the activity of acid phosphatases, we used chromogenic substrate 4-nitrophenyl phosphate (Sigma-Aldrich). The substrate was prepared in 50 mM (pH 5.0) acetate buffer at a concentration of 5 mM. Collected fluid (150 μl) was added to 400 μl of 50 mM acetate buffer (pH 5.0) and mixed with 400 μl of the substrate. For control, 400 μl of the substrate solution was mixed with 550 μl of the acetate buffer. Mixed samples were incubated at 25 °C for 1 h, and then 160 μl of 1.0 M NaOH was added to terminate the reaction. Absorbance was measured at 405 nm with a double-beam spectrophotometer Specord 250 Plus (Analytik Jena).

### Western blotting

To detect and quantify aspartic protease and type III chitinase, polyclonal antibodies against these proteins were raised in rabbits by Genscript (Piscataway, NJ, USA). The following amino acid sequences (epitopes) were synthesized: aspartic protease, (NH_2_-)SAIMDTGSDLIWTQC(-CONH_2_) (based on sequences BAD07474.1, BAD07474.1, AFV26025.1, AFV26024.1 from *N. gracilis* and *N. mirabilis*), and type III chitinase, (NH_2_-)CWSKYYDNGYSSAIKD(-CONH_2_) (based on sequence ABF74624.1 from *N. rafflesiana*), respectively (Genscript, Piscataway, NJ, USA). Because we were not able to detect a positive reaction for aspartic proteases despite their presence in digestive fluid in *S. purpurea* (Fukushima *et al*., 2017), we synthesized new epitope based on the sequence of aspartic protease (BAW35441.1) directly from *S. purpurea* ssp. *venosa*: (NH_2_-)QQQDPTFDPSKSTTC(-CONH_2_) (Genscript). All sequences were coupled to a carrier protein (keyhole limpet haemocyanin, KLH) and injected into two rabbits each. The terminal cysteine of the peptide was used for conjugation. The rabbit serum was analysed for the presence of antigen-specific antibodies using an ELISA.

The digestive fluid collected for the enzyme assays was subjected to western blotting. The samples were heated and denatured for 30 min at 70 °C and mixed with modified Laemmli sample buffer to a final concentration of 50 mM Tris–HCl (pH 6.8), 2% SDS, 10% glycerol, 1% β-mercaptoethanol, 12.5 mM EDTA, and 0.02% bromophenol blue. The same volume of digestive fluid was electrophoresed in 10% (v/v) SDS polyacrylamide gel (Schägger, 2006). The proteins in the gels were either visualized by silver staining (ProteoSilver; Sigma-Aldrich) or transferred from the gel to a nitrocellulose membrane (Bio-Rad) using a Trans-Blot SD Semi-Dry Electrophoretic Transfer Cell (Bio-Rad). After blocking in TBS-T containing 5% BSA overnight, the membranes were incubated with the primary antibody for 1 h at room temperature, and after washing, the membrane was incubated with the secondary antibody: goat antirabbit IgG (H + L) horseradish peroxidase conjugate (Bio-Rad). Blots were visualized and chemiluminescence was quantified by an Amersham Imager 600 gel scanner (GE Healthcare Life Sciences, Tokyo, Japan).

### Pigment quantification

Twenty days after coronatine application, five control and five coronatine-treated *C. follicularis* pitchers were harvested for pigment quantification. Because the coronatine-treated pitchers became red, they were investigated for the presence of anthocyanins and chlorophylls. Because the two groups of pigments have different pH optima for quantification, they were extracted separately (Sims and Gamon, 2002).

For anthocyanin determination, 100 mg fresh weight (FW) of pitcher tissue was ground with a mortar and pestle and extracted with 6 ml of −20 °C methanol–HCl (0.1% HCl v/v). The samples were centrifuged at 6000 *g* at 4 °C for 10 min. Spectrophotometric analysis of the supernatant was conducted with a double-beam spectrophotometer, Specord 250 Plus (Analytik Jena), at 532 nm. Because the red peak absorbance for degraded chlorophylls in these extracts occurred at 653 nm and had a tail that overlaps with the anthocyanin peak at 532 nm, the absorbance at 532 nm was corrected by subtracting 24% of the Chl maximum absorbance at 653 nm from the maximum anthocyanin absorbance at 532 nm. Total anthocyanin content was then calculated using this corrected absorbance and a molar extinction coefficient for anthocyanin 30 000 l mol^−1^ cm^−1^ (Murray and Hackett, 1991; Sims and Gamon, 2002).

For chlorophyll *a*+*b* determination, 150 mg FW of pitcher tissue was ground in a mortar and pestle and extracted with 6 ml of 4 °C 80% acetone (v/v). The samples were centrifuged at 6000 *g* at 4 °C for 10 min. Spectrophotometric analysis of the supernatant was conducted with the same spectrophotometer as mentioned above at 537 nm for anthocyanins, 647 nm for Chl *b* and 663 nm for Chl *a*. Because the high content of anthocyanins interferes with chlorophyll *a*+*b* calculation, we used the equations of Sims and Gamon (2002) that take into account the contribution of anthocyanins at 537 nm wavelength to Chl *a* and Chl *b* absorbance.

## Results

### Electrical signals

**Fig. 2.**
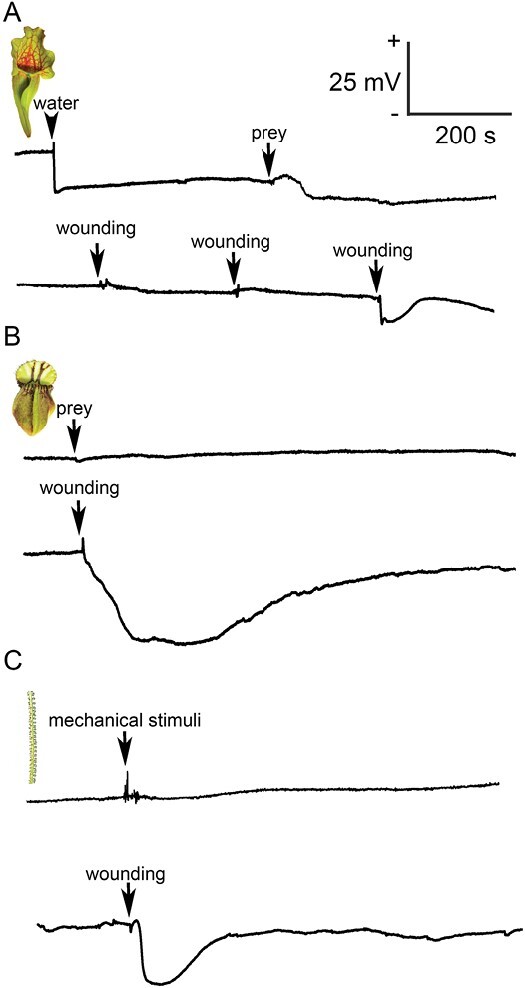
Extracellular recording of electrical signals in carnivorous plants. (A) Changes of membrane potential in response to water addition (arrowhead, upper trace), prey (arrow, upper trace), and repeated wounding with a needle 1–2 cm distance from the recording electrode (arrows, lower trace) in the pitcher of *Sarracenia purpurea*. The response to water and prey addition is probably not physiological, as it was recorded also in dead withered trap tissue. (B) Recording of membrane potential in response to prey addition (arrow, upper trace) or wounding 1–2 cm distance from the recording electrode (arrow, lower trace) in *Cephalotus follicularis*. (C) Recording of membrane potential from the stalked gland of *Drosophyllum lusitanicum* in response to mechanical contact (arrow, upper trace) or wounding of leaf 1–2 cm distance from the recorded stalked gland (arrow, lower trace).

In contrast to *C. follicularis*, *S. purpurea* does not produce a significant level of digestive fluid and relies on the collection of rainwater. For this purpose, *S. purpurea* lacks a pitcher lid in contrast to the majority of pitcher plants, where the lid prevents dilution of digestive fluid by rain (Fig. 1A). Adding distilled water into *S. purpurea* pitcher, simulating rain, resulted in fast hyperpolarization of the membrane potential (negative voltage shift recorded extracellularly, representing intracellular depolarization). Prey addition a few minutes later had a small effect on membrane potential in the opposite direction (Fig. 2A). What type of signals these recorded potential changes represent is not clear. Repeating the experiment with a withered dead pitcher gave a similar response (Supplementary Fig. S1), indicating that the signal probably does not have a physiological origin. The pitcher of *S. purpurea* did not generate electrical signals in response to wounding every time; they were recorded only occasionally and resemble a typical action potential (AP) with low amplitude (Fig. 2A). Sometimes, several dozen APs were triggered in response to a single wounding (data not shown). No changes of membrane potential were recorded in the pitchers of *C. follicularis* (Fig. 2B) in response to prey addition, but wounding generated hyperpolarization of the membrane potential (negative voltage shift recorded extracellularly, representing intracellular depolarization) representing a typical variation potential (VP; Fig. 2B). The stalked glands of *D. lusitanicum* (Fig. 1C) did not generate electrical signals in response to touch stimuli. A typical VP was triggered in stalked gland in response to leaf wounding (Fig. 2C).

### Phytohormone accumulation

**Fig. 3.**
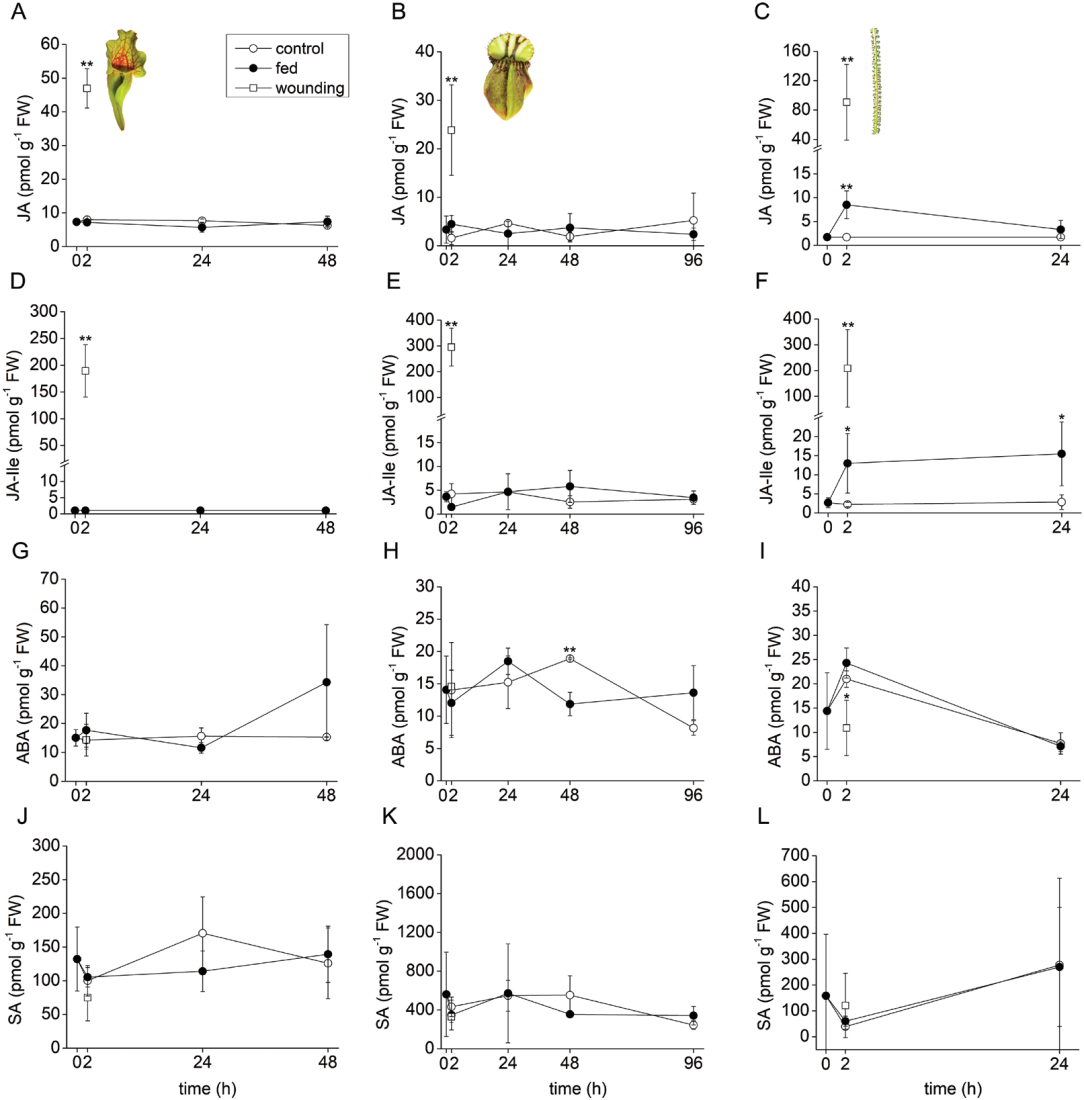
Phytohormone accumulation in response to feeding and wounding in carnivorous plants. (A–C) Jasmonic acid (JA); (D–F); isoleucine conjugate of jasmonic acid (JA-Ile); (G–I) abscisic acid (ABA); (J–L) salicylic acid (SA). (A, D, G, J) *Sarracenia purpurea* ssp. *venosa*; (B, E, H, K) *Cephalotus follicularis*; (C, F, I, L) *Drosophyllum lusitanicum*. Open circles, control plants; closed circles, fed plants; open squares, wounded plants. Significant differences (Student’s *t*-test) between the control and fed or wounded plants at the same time points are indicated: *P<0.05, **P<0.01. Data are means ±SD, *n*=4.

The pitcher plants *S. purpurea* and *C. follicularis* did not accumulate a significant level of JA or JA-Ile in response to feeding, in contrast to *D. lusitanicum* (Fig. 3A–F). But 2 h after wounding, the level of JA and JA-Ile significantly increased in all studied plants, documenting the ability of carnivorous plants to synthesize JAs in response to damaging stimuli, as is well known in non-carnivorous plants (Fig. 3A–F). The other stress-related hormones, ABA and SA, were not significantly increased in response to feeding or wounding (Fig. 3G–L). Only the content of ABA was significantly decreased 2 h after wounding in *D. lusitanicum*, and 48 h after feeding in *C. follicularis* (Fig. 3H, I).

### Proteomic composition of digestive fluid

Because the protein composition of digestive fluids in *S. purpurea* and *C. follicularis* has been characterized previously, we focused on the secretome of *D. lusitanicum*. Using the *de novo* identification approach, 45 different proteins were unambiguously identified in the *D. lusitanicum* digestive fluid (Table 1; Supplementary Dataset S1). From all those, 15 proteins exhibited extracellular localization suggesting a secretory origin. These proteins mainly had enzymatic properties. According to the assigned annotation, they comprised six different catabolic activities previously reported in carnivorous plant digestive fluids (Table 1; Supplementary Table S2). It should be noted that the obtained results are pioneering rather than comprehensive as our *de novo* identification approach was based on a protein homology search within the *Caryophyllales* order. Additional protein components of the digestive fluid can thus be present that are missing in the current dataset due to low sequence similarity. Interestingly, higher spectral counts were detected in fed or coronatine-treated samples for some of these proteins (e.g. purple acid phosphatase, serine carboxypeptidase), suggesting their up-regulation by these specific stimuli (see PSM values in Table 1 and Supplementary Table S2).

**Table 1.** *Drosophyllum lusitanicum* digestive fluid proteins identified by mass spectrometry

MS data peaks software searches					pBLAST homology searches		
Homologous protein accession^*a*^	Homologous protein name	Organism	Peptides/SC^*b*^	PSMs^*c*^	Homologous protein accession^*d*^	Homologous protein name	E-value/bit score/ % positives
BAW35430	Purple acid phosphatase	*Nepenthes alata*	5/8	3/28/52	Q5MAU8	Probable inactive purple acid phosphatase 27	1.10 × 10^−107^/338/52.23
KAH9619639	Hypothetical protein	*Heliosperma pusillum*	4/9	7/20/38	Q8H1R2	Probable inactive purple acid phosphatase 24	7.65 × 10^−95^/304/54.97
MCK0600380	Acid phosphatase	*Opuntia streptacantha*	2/13	6/20/35	Q9LMX4	Probable inactive purple acid phosphatase 1	4.63 × 10^−59^/191/84.8
XP_021758194	α-Galactosidase 3-like	*Chenopodium quinoa*	1/2	12/13/16	Q8VXZ7	α-Galactosidase 3	0/660/87.12
XP_021859482	Serine carboxypeptidase II-3-like	*Spinacia oleracea*	1/2	0/5/15	A0A0C3VJP4	Serine carboxypeptidase 1	2.39 × 10^−164^/474/70.38
XP_048495078	α-Galactosidase 1 isoform X1	*Beta vulgaris*	2/5	15/20/6	Q9FT97	α-Galactosidase 1	0/634/87.53
KAH9624815	Hypothetical protein	*Heliosperma pusillum*	3/6	3/11/6	P32826	Serine carboxypeptidase-like 49	0/698/82.72
XP_021852453	Probable inactive purple acid phosphatase 27	*Spinacia oleracea*	3/6	1/3/3	Q5MAU8	Probable inactive purple acid phosphatase 27	1.77 × 10^−107^/337/50.94
XP_021761383	Basic endochitinase-like	*Chenopodium quinoa*	1/3	1/1/3	P29061	Basic endochitinase	5.71 × 10^−127^/363/81.07
ABB89525	Glucanase	*Nepenthes khasiana*	1/3	0/0/2	P52408	Glucan endo-1,3-β-glucosidase, basic isoform	2.92 × 10^−113^/332/72.29
XP_021743448	Probable glucan 1 3-β-glucosidase A	*Chenopodium quinoa*	1/4	1/1/1	P85924	Glucan 1,3-β-glucosidase	0.032/32.7/88.24
XP_021867395	GDSL esterase/lipase 7-like	*Spinacia oleracea*	1/2	0/0/1	Q8LFJ9	GDSL esterase/lipase 7	2.51 × 10^−167^/471/79.12
XP_021719798	Cysteine proteinase inhibitor 1-like	*Chenopodium quinoa*	1/10	2/4/0	P86472	Cysteine proteinase inhibitor 1	2.04 × 10^−30^/105/71.58
BAM28610	Class III chitinase	*Nepenthes alata*	1/4	1/0/0	P36910	Acidic endochitinase SE2	1.20 × 10^−132^/377/80.56
XP_010676513	Protein P21	*Beta vulgaris*	1/5	1/0/0	P83958	Thaumatin-like protein	4.51 × 10^−106^/305/75

For more details see Supplementary Table S2 and Supplementary Dataset S1.

^*a*^ NCBI database accession.

^*b*^ SC, sequence coverage (%).

^*c*^ PSMs, peptide-spectrum matches for control, feeding experiment, and coronatine treatment, respectively.

^*d*^ UniProtKB/Swiss-Prot database accession.

### Enzyme activities

**Fig. 4.**
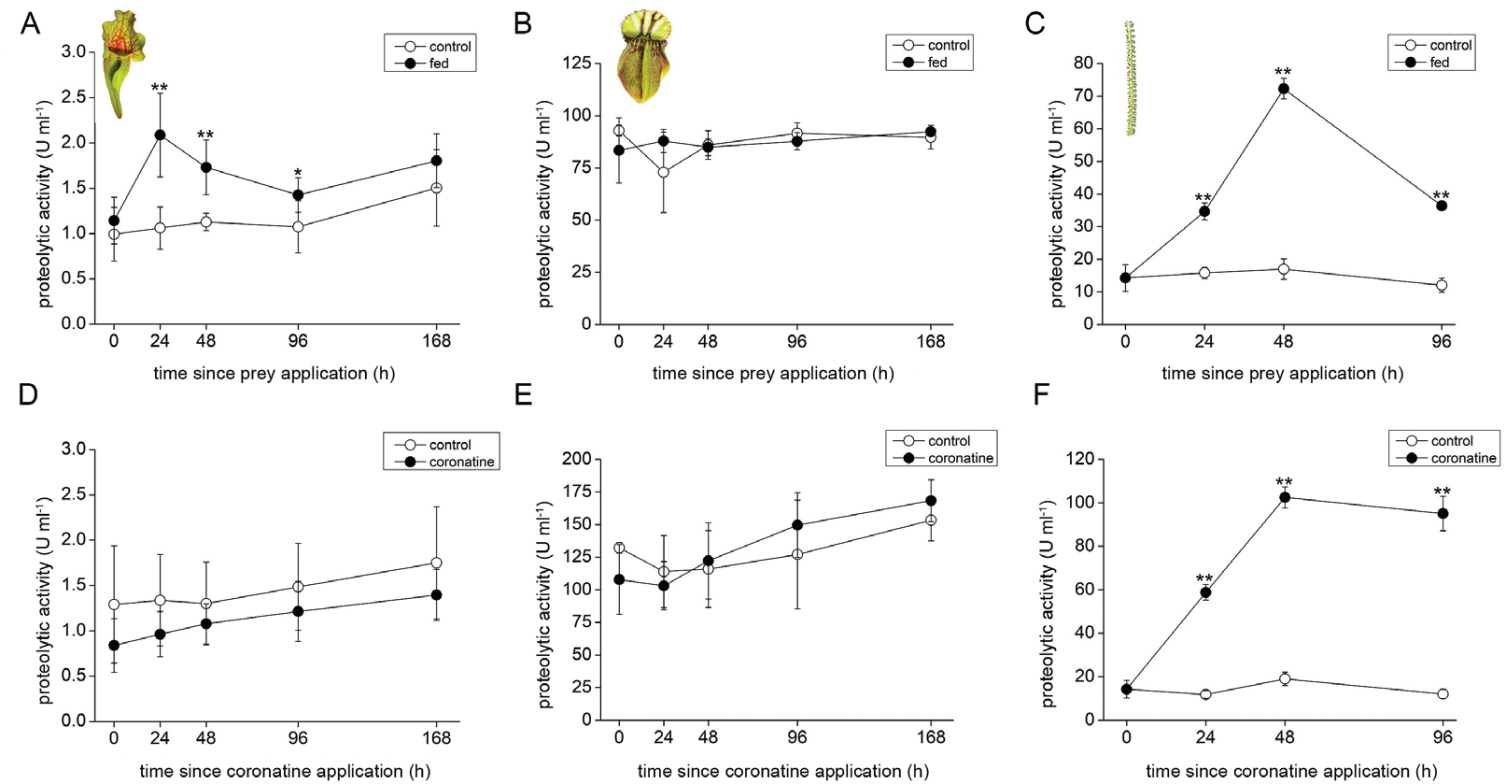
Proteolytic activities in the digestive fluid of carnivorous plants. (A–C) Feeding with insect prey; (D–F) coronatine application; (A, D) *Sarracenia purpurea* ssp. *venosa*; (B, E) *Cephalotus follicularis*; (C, F) *Drosophyllum lusitanicum*. Open circles, control plants; closed circles, fed or coronatine-treated plants. Data are means ±SD, *n*=4. Significant differences (Student’s *t*-test) between the control and fed samples at the same time point are indicated: *P<0.05, **P<0.01.

The overall proteolytic activity was significantly higher in fed *S. purpurea* pitchers in comparison with the control (Fig. 4A). In *C. follicularis*, the proteolytic activity was constitutive (Fig. 4B). In *D. lusitanicum* proteolytic activity significantly increased in response to feeding (Fig. 4C). In *S. purpurea*, phosphatase activity in digestive fluid increased over time in both control and fed pitchers without significant differences (Supplementary Fig. S2A). In *C. follicularis* the phosphatase activity was constitutive without any effect of prey addition on the enzyme activities (Supplementary Fig. S2B). In *D. lusitanicum* the activity significantly increased in response to feeding (Supplementary Fig. S2C).

To reveal the regulatory role of JAs on enzyme activities, 100 µM coronatine (a molecular agonist of JA-Ile) was applied exogenously and the enzyme activities were measured. As can be expected from analyses of phytohormone tissue levels, the strong induction of proteolytic and phosphatase activities was detected only in *D. lusitanicum* (Fig. 4F; Supplementary Fig S2F). The pitcher plants *S. purpurea* and *C. follicularis* did not increase enzyme activities (Fig. 4D, E; Supplementary Fig. S2D, E) but the traps of *C. follicularis*, in which the coronatine was applied, became red 20 d after its application. These traps contained significantly less chlorophyll *a*+*b* and accumulated an increased concentration of anthocyanins (Supplementary Fig. S3). As a positive control for pitcher traps, 100 µM coronatine induced enzyme activities in pitcher plant *Nepenthes* × *Mixta* (Supplementary Fig. S4A, B).

### Enzyme abundance in response to feeding and coronatine application

**Fig. 5.**
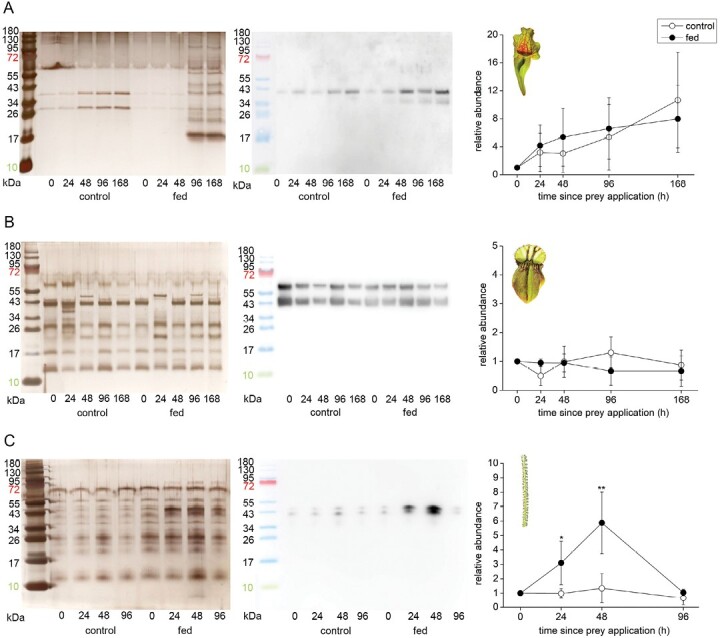
Immunodetection of aspartic protease in the digestive fluid in response to feeding in carnivorous plants. (A) *Sarracenia purpurea* ssp*. venosa*; (B) *Cephalotus follicularis*; (C) *Drosophyllum lusitanicum*. The proteins were separated in 10% (v/v) SDS-PAGE and silver stained (left) or subjected to western blot analysis (middle) and the chemiluminescence signal intensity was quantified (right). Both bands were used for quantification. Signal intensity at zero time point was set as 1. Open circles, control plants; closed circles, fed plants. Representative gels and blots are shown. Data are means ±SD, *n*=4. Significant differences (Student‘s *t*-test) between the control and treated samples at the same time point are indicated: *P<0.05, **P<0.01. The protein marker in the picture of immunodetected protein was added manually based on the merged image from the gel scanner. For immunodetection of type III chitinase in *S. purpurea* ssp. *venosa* from the same samples, see Supplementary Fig. S5.

In *S. purpurea*, the concentration of aspartic protease in digestive fluid increased over time regardless of pitcher feeding status (Fig. 5A). On the other hand, the aspartic protease was constitutively present in digestive fluid of control as well as fed pitchers in *C. follicularis* (Fig. 5B). In contrast, aspartic protease in *D. lusitanicum* was clearly up-regulated in response to feeding (Fig. 5C).

**Fig. 6.**
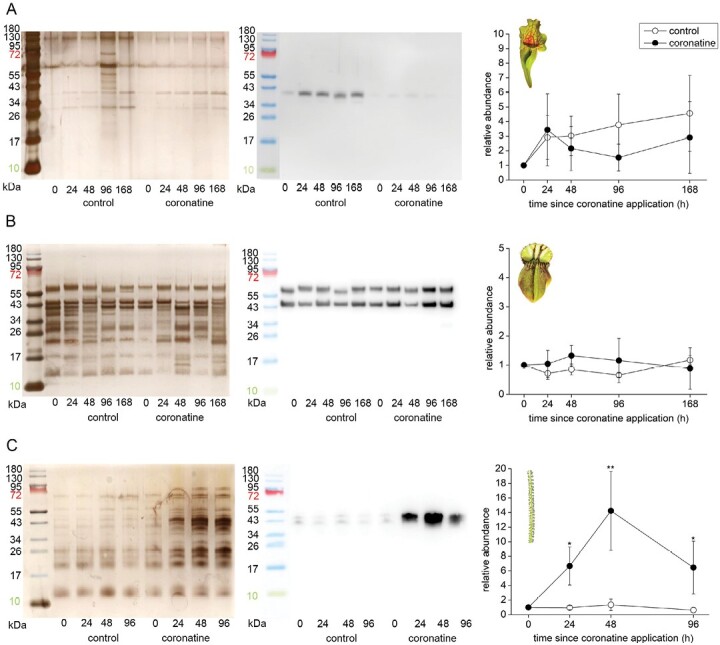
Immunodetection of aspartic protease in the digestive fluid in response to coronatine application in carnivorous plants. (A) *Sarracenia purpurea* ssp. *venosa*; (B) *Cephalotus follicularis*; (C) *Drosophyllum lusitanicum*. The proteins were separated in 10% (v/v) SDS-PAGE and silver stained (left) or subjected to western blot analysis (middle) and the chemiluminescence signal intensity was quantified (right). Both bands were used for quantification. Signal intensity at zero time point was set as 1. Open circles, control plants; closed circles, coronatine-treated. Representative gels and blots are shown. Data are means ±SD, *n*=4. Significant differences (Student‘s *t*-test) between the control and treated samples at the same time point are indicated: *P<0.05, **P<0.01. The protein marker in the image of immunodetected protein was added manually based on the merged image from the gel scanner. For immunodetection of type III chitinase in *S. purpurea* ssp. *venosa* from the same samples, see Supplementary Fig. S6.

In coronatine-treated traps, the clear up-regulation of aspartic protease was detected only in the case of *D. lusitanicum* (Fig. 6C). The abundance of aspartic proteases in digestive fluids is in accordance with the measurements of proteolytic activity (Fig. 4F).

Among the other antibodies available from our previous studies on carnivorous plants we were successful in immunodetection of type III chitinase in *S. purpurea*. The amount of chitinase III enzyme in digestive fluid increased over time in both control and fed pitchers similarly to aspartic protease (Supplementary Fig. S5), but this upward trend was significantly reduced when coronatine was added into the digestive fluid (Supplementary Fig. S6).

### Developmental control of enzyme secretion

Because enzyme abundance increased over time regardless of feeding status in *Sarracenia purpurea*, either the enzyme production is regulated developmentally or water might act as a stimulus. To distinguish between these two possibilities, we performed an experiment in which the first pitcher received water immediately after opening and the second pitcher, which had opened during the same day, received water 14 d later. Immediately after the addition of water, the aspartic protease was clearly detectable in neither trap. However, the enzyme was clearly detectable on the 14th day in the first trap, but not on the 14th day in the second trap, indicating that water may act as a stimulus (Supplementary Fig. S7). A different response was found for type III chitinase. The enzyme was clearly detectable after water addition in the second trap but not in the first trap, indicating rather developmental control of chitinase III secretion (Supplementary Fig. S8). Thus, *S. purpurea* may combine several control mechanisms to optimize enzyme secretion: prey, developmental, and water-mediated stimulation of enzyme secretion.

In *C. follicularis*, the aspartic protease was present in high abundance already in freshly open pitchers and decreased over time (Supplementary Fig. S9).

## Discussion

In this study, we investigated the regulatory role of JAs on enzyme activities in three distantly related taxa of carnivorous plants. Previously, the role of JAs in the regulation of enzyme secretion has been confirmed in four genera of carnivorous plants (*Aldrovanda*, *Dionaea*, *Drosera*, *Nepenthes*), which are all related and belong to the same order, *Caryophyllales* (Nakamura *et al*., 2013; Libiaková *et al*., 2014; Mithöfer *et al*., 2014; Buch *et al*., 2015; Böhm *et al*., 2016a, b; Yilamujiang *et al*., 2016; Krausko *et al*., 2017; Pavlovič *et al*., 2017; Jakšová *et al*., 2020, 2021). In this study, we were interested in whether JA signalling regulates enzyme activities outside the order *Caryophyllales*.

First, we investigated the species *S*. *purpurea* ssp*. venosa*. The data on the ability of *S. purpurea* to produce endogenous plant-derived enzymes are contradictory. Some studies showed that pitchers have no ability to produce plant-derived enzymes (Koller-Peroutka *et al*., 2019); others showed that the plant secrets limited amount of digestive enzymes into pitcher fluid but with a primary role of microbes in the digestion of prey (Luciano and Newell, 2017; Young *et al*., 2018). Recently, Fukushima *et al*. (2017) unequivocally confirmed the presence of plant-derived enzymes in the digestive fluid of *S. purpurea* and our data are in agreement with their findings. The plant produces only a few droplets of digestive fluid and due to the absence of a pitcher lid, typical pitchers may contain up to 30 ml of rainwater (Adlassnig *et al*., 2011). Thus, the enzymes are strongly diluted in nature, which hampered their identification using less sensitive methods (Koller-Peroutka *et al*., 2019), and digestion is taken over by microorganisms living in these phytotelmata-like traps. We did not observe stimulation of phosphatase (Supplementary Fig. S2A), but did observe proteolytic activity (Fig. 4A) in response to feeding in accordance with the study of Young *et al*. (2018). Other studies showed stimulation of both enzyme activities in response to prey or protein additions (Gallie and Chang, 1997; Luciano and Newell, 2017). However, there is a clear trend toward the increased abundance of aspartic protease and chitinase III in control as well as fed traps over time (Fig. 5A; Supplementary Fig. S5). This agrees with previous studies that documented that nuclease activities in *S. purpurea* are first developmentally regulated and only later are responsive to chemical stimuli from prey (Gallie and Chang, 1997; Luciano and Newell, 2017). However, our additional experiment supports also the option that water may also act as a stimulus for aspartic protease (Supplementary Fig. S7). A recent study on Arabidopsis showed that a drop of water can induce JA-independent expression of defence-related genes, and genes encoding aspartate proteases are among them (Matsumura *et al*., 2022), though on the other side, a drop of water on the sundew plant *D. capensis* did not trigger significant secretion of aspartic protease (Pavlovič *et al*., 2023). Using all these studies it can be concluded that *S. purpurea* pitchers combine developmental, water- and prey-induced enzyme regulation, but the JAs are not involved in signalling (Supplementary Fig. S10).

In contrast to the situation in *S. purpurea*, even closed immature traps of *C. follicularis* already contain a stable level of plant-derived fluid with digestive enzymes (Supplementary Fig. S9; Takahashi *et al*., 2009; Adlassnig *et al*., 2011; Fukushima *et al*., 2017). The pitchers are covered by the lid protecting the digestive fluid against enzyme dilution. Indeed, comparing the enzyme activities between *S. purpurea* and *C. follicularis* confirmed strongly diluted fluid and enzymes in the former (Fig. 4A, B). The enzyme activities and abundance of aspartic protease in the pitcher fluid did not respond to prey addition, indicating that the enzyme activity is constitutive. The JAs did not accumulate and external application of coronatine had no effect on enzyme activities and abundance of aspartic protease (Figs 4B, E, 6B). In accordance with our study, Nishimura *et al*. (2013) reported constitutive expression of S-like RNase in *C. follicularis* in contrast to its induced expression in *D. muscipula*. In this view, the traps of *C. follicularis* are completely passive and not responsive to prey. We observed that coronatine-treated pitchers turned red after 2 weeks (Supplementary Fig. S3), indicating JA-mediated accumulation of anthocyanins in *C*. *follicularis*. Jasmonate-induced accumulation of anthocyanins in non-carnivorous plants is well known (Shan *et al*., 2009; An *et al*., 2021).

Although the non-related genus of pitcher plant *Nepenthes* accumulated JA and JA-Ile in response to experimental feeding (Yilamujiang *et al*., 2016) and increased enzyme activity in response to JA or coronatine application, the exogenously added JA or coronatine induced expression of digestive enzyme and enzyme activity in digestive fluid only very slightly, 1–4-fold (Supplementary Fig. S4) (Buch *et al*., 2015; Yilamujiang *et al*., 2016; Vadassery *et al*., 2019). In contrast, stimulated *Drosera*, *Dionaea*, and *Drosophyllum* showed induced expression of digestive enzymes and enzyme activities to several tens or even hundreds of times in comparison with control plants (Fig. 4F) (Bemm *et al*., 2016; Krausko *et al*., 2017; Pavlovič *et al*., 2017) indicating that functional constraint on the JA signalling used for botanical carnivory is relaxed in *Nepenthes*. Taking into account that pitcher traps have likely evolved from sticky traps (Fleischmann *et al.*, 2018), *Nepenthes* probably only inherited JA signalling from its sticky ancestor, and with its new pitcher form it is not further necessary, and other genera of pitcher plants (e.g. *Cephalotus*, *Sarracenia*) have not co-opted JA signalling at all. Maybe passive pitcher traps are not under such strong selective pressure in comparison with active hunters with snap-traps or sticky traps that have to hurry and often form a digestive cavity only in response to prey capture through JA signalling. Recently it was shown that pitchers of *Nepenthes* can maintain the level of digestive enzymes in pitcher fluid over time without any prey stimuli by an autoregulation mechanism (Wan Zakaria *et al*., 2019; Goh *et al*., 2020). Moreover, besides digestive enzymes, pitcher and bladder suction traps (e.g. *Utricularia*) often rely on microorganism-mediated digestion, which may be in conflict with the use of JA signalling pathway involved in plant defence mechanisms against microbes (Renner *et al*., 2018).

The situation in *D. lusitanicum* is completely different from the studied pitcher plants. We showed clear induction of enzyme activities and enzyme abundance indicating a stimulatory mode of secretion regulated by JAs, as is known in *Drosera* and *Dionaea* plants (Figs 3C, F, 4C, F, 5C, 6C; Table 1; Supplementary Table S2) (Krausko *et al*., 2017; Pavlovič *et al*., 2017). However, in contrast to these plants, stalked glands and leaf traps of *D. lusitanicum* are not capable of any movement, and are not able to generate any fast APs in response to mechanical stimulation in contrast to sundew plants (Fig. 2C) (Williams and Pickard, 1972; Williams, 1976; Krausko *et al*., 2017). Although there is a clear connection between fast electrical signals (i.e. APs and VPs) and JA accumulation in non-carnivorous (Mousavi *et al*., 2013; Farmer *et al*., 2020) as well as in carnivorous plants (Krausko *et al*., 2017; Pavlovič *et al*., 2017), the JAs can accumulate also without fast electrical signalling in passive pitcher traps of *Nepenthes* (Yilamujiang *et al*., 2016). A recent study has shown that chemical signals from prey (chitin and protein) are more important for stable, high, and long-term accumulation of JAs and digestive enzymes in *Dionaea* than mechanical stimuli alone (Jakšová *et al*., 2020). Similarly, *D. lusitanicum* may respond mainly to chemical stimuli from insect prey, but Ca^2+^ signalling in response to mechanical touch, as is known in Arabidopsis trichome or *Drosera* tentacles, cannot be ruled out (Matsumura *et al*., 2022; Pavlovič, 2022; Procko *et al*., 2022). In accordance with our study, an ultrastructural study on digestive glands of *D*. *lusitanicum* showed an increased surface density of rough endoplasmic reticulum as well as the number of Golgi stacks after stimulation, indicating a sharp rise in the rate of synthesis and secretion of digestive enzymes after stimulation (Vassilyev, 2005).

All three investigated genera of carnivorous plants in this study use aspartic protease for protein digestion. The aspartic proteases are used for protein digestion also by the genera *Drosera*, *Dionaea*, *Nepenthes*, and *Pinguicula* (Athauda *et al*., 2004; Schulze *et al*., 2012; Takahashi *et al*., 2012; Krausko *et al*., 2017; Kocáb *et al*., 2020). Because digestive enzymes in *C. follicularis* and *S. purpurea* have already been identified before (Fukushima *et al*., 2017), we focused our attention on *D. lusitanicum*. The digestive enzymes we found in this species are very similar to those described from other genera of carnivorous plants (Table 1; Supplementary Table S2) (Schulze *et al*., 2012; Krausko *et al*., 2017; Lee *et al*., 2017), confirming the hypothesis that unrelated taxa of carnivorous plants use similar enzymes for prey digestion (Fukushima *et al*., 2017). They often belong to the group of pathogenesis-related (PR) proteins (Mithöfer, 2011). They were co-opted from plant defence to botanical carnivory several times independently in distantly related genera of carnivorous plants, in contrast to JA signalling regulating their activity.

**Fig. 7.**
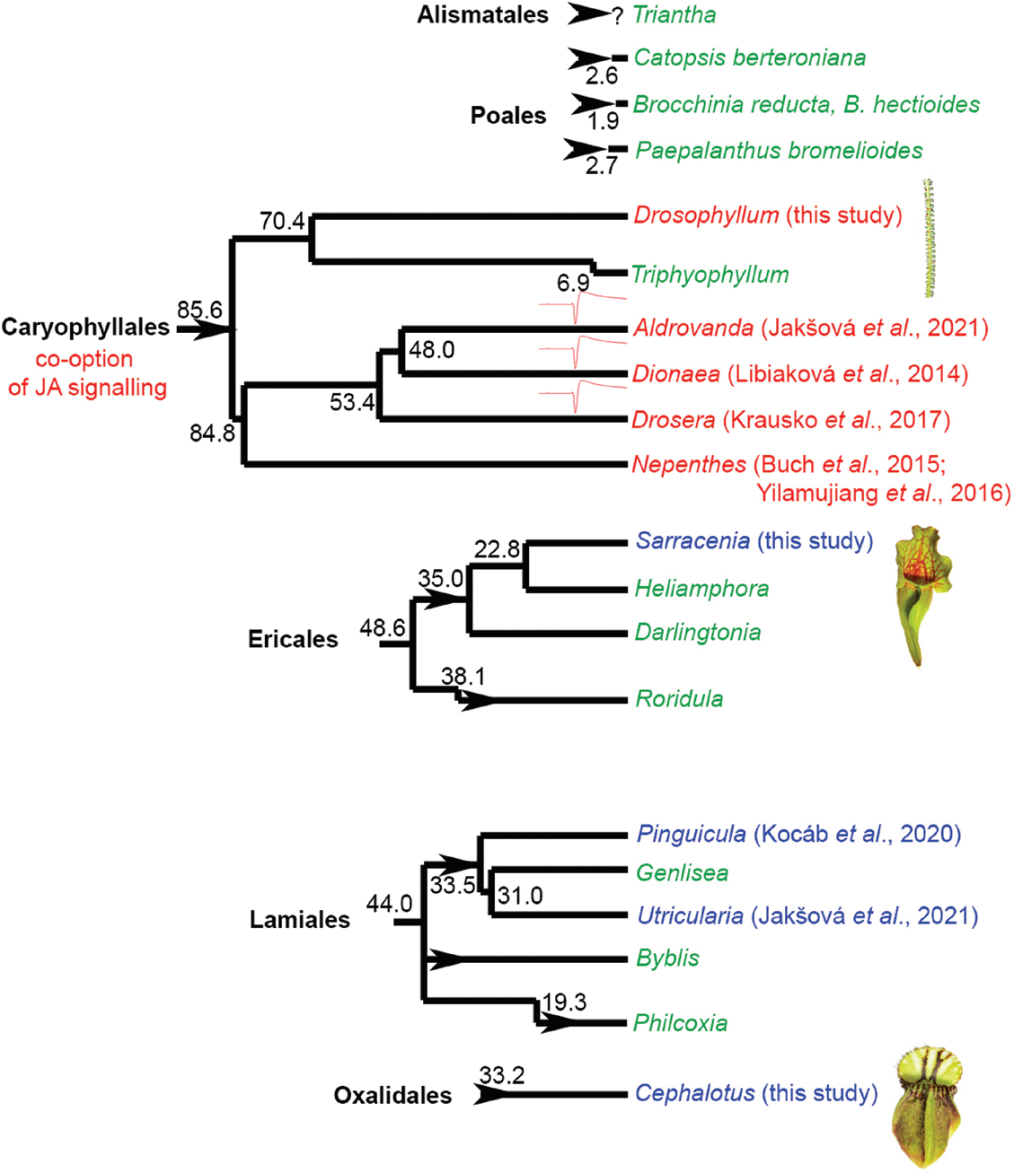
Jasmonate signalling in carnivorous plants from a phylogenetic point of view. Carnivorous plants have evolved at least 11 times independently (arrowheads) in six orders of angiosperms. The genera of carnivorous plants in which the jasmonates are involved in the regulation of enzyme secretion are depicted in red. The genera in which jasmonates do not play any role in the regulation of enzyme activities are depicted in blue. The genera that have not yet been investigated in this respect are depicted in green. The genera that use fast electrical signalling are marked by a symbol of action potential. It is clearly seen, that involvement of jasmonates in the regulation of enzyme activities forms a clear cluster in the order *Caryophyllales*. The genera that use fast electrical signals forms a clear cluster within the *Droseraceae* family. The estimated phylogenetic age depicted is in million years ago according to Fleischmann *et al*. (2018). Note that there are several hypotheses about the phylogenetic position of individual families within *Caryophyllales* (see e.g. Renner and Specht, 2011). References that first time showed (or did not) increased endogenous levels of bioactive isoleucine conjugate of jasmonic acid (JA-Ile) in response to prey application and enzyme secretion in response to exogenous application of jasmonates or coronatine are shown.

The involvement of JAs in botanical carnivory from the phylogenetic point of view is shown in Fig. 7. Although JA signalling in carnivorous plants has been studied only in 9 out of 20 genera of carnivorous plants so far, it seems that JAs have been co-opted for botanical carnivory only in the oldest order of carnivorous plants, *Caryophyllales*, although more studies are needed. Carnivory arose more recently and four times independently in monocots in the orders *Poales* (genera *Catopsis*, *Brocchinia*, *Paepalanthus*) and *Alismatales* (genus *Triantha*; Lin *et al*., 2021); however, we still do not have experimental evidence that these plants use endogenous digestive enzymes for prey digestion. Instead, as in the case of other plant phytotelmata (except *Triantha*), they probably rely on symbiotic microorganisms (Adlassnig *et al*., 2011). Another genus of carnivorous plants that does not produce digestive enzymes is *Roridula* (Ellis and Midgley, 1996; Plachno *et al*., 2009). Two species of carnivorous *Roridula* in the order *Ericales* rely on symbiotic bugs of the genus *Pameridea*, which consume prey captured by the sticky resin mucilage of the plant, and the plant absorbs nitrogen from the liquid hemipteran faeces directly through the cuticle (Anderson, 2005). Thus, it is unlikely that these genera have co-opted JAs for regulation of something that they do not produce. Endogenous enzyme secretion is dubious also in the genera *Darlingtonia* and *Heliamphora*, but we cannot completely rule it out as some studies indicate (Jaffe *et al*., 1996; Koller-Peroutka *et al*., 2019). But because their sister genus *Sarracenia* does not use JAs for enzyme regulation, this is also highly likely the same for *Darlingtonia* and *Heliamphora*. The underground traps of *Genlisea* complicate rigorous controlled feeding experiments. It is also still not clear if *Genlisea* produces its own endogenous digestive enzymes or relies on symbiotic microorganisms. The sister taxa *Utricularia* and *Pinguicula* produce endogenous enzymes, but do not use JAs for their induction (Kocáb *et al*., 2020; Jakšová *et al*., 2021). Due to their rarity, two genera of carnivorous plants within *Lamiales* with an independent origin of carnivory, *Byblis* and *Philcoxia*, remain also a totally unexplored group of carnivorous plants in regard to digestive physiology. It was suggested that *Byblis* resembles *Roridula* and relies on digestive mutualisms (Ellis and Midgley 1996), but Li *et al*. (2023) recently documented protease activity in *Byblis guehoi*, which makes *Byblis* an interesting object for further investigation.

Taking into account that carnivory in *Caryophyllales* (including families *Droseraceae*, *Nepenthaceae*, *Drosophyllaceae*, and *Dioncophyllaceae*) has evolved only once (Fleischmann *et al*., 2018), it is tempting to assume that JA signalling for botanical carnivory has been co-opted also only once in the last common ancestor before 85.6 mya. However, the monophyletic origin of carnivorous plants within *Caryophyllales* has recently been questioned by Palfalvi *et al*. (2020) based on genomic studies within *Droseraceae* and transcriptomic data for *Nepenthes alata* (de Vries and de Vries, 2020). They suggested that the co-option of JA signalling for botanical carnivory has evolved in the most recent common ancestor of *Droseraceae* (including genera *Drosera*, *Dionaea*, and *Aldrovanda*). However, our biochemical data clearly showed that the monotypic family *Drosophyllaceae*, including *D. lusitanicum*, uses JA signalling for botanical carnivory. Also, previous studies on *Nepenthes* showed the involvement of JAs in botanical carnivory in the family *Nepenthaceae* (Buch *et al*., 2015; Yilamujiang *et al*., 2016; Vadassery *et al*., 2019). Although the possibility, that JA signalling has been co-opted three times independently in families *Droseraceae*, *Nepenthaceae* and *Drosophyllaceae* cannot be completely ruled out, we consider this scenario as unlikely with an unnecessary complexity. This can be supported also by the fact that no other phylogenetic lineage of carnivorous plants investigated so far has co-opted JAs for botanical carnivory. To complement the picture of JA signalling in *Caryophyllales* it remains to investigate the monotypic genus *Triphyophyllum* (family *Dioncophyllaceae*) including *T. peltatum*. However, this plant is extremely rare in nature and is under-represented in private and scientific collections of carnivorous plants. Moreover, it produces only a few (three) carnivorous leaves, with mucilage secretion resembling *Drosophyllum* only during a short part of its juvenile phase and not frequently observed (Green *et al*., 1979; Rembold *et al*., 2010), which hampers serious experimental investigation. The part-time carnivore *T. peltatum* might be in an evolutionary transition away from carnivory, because its consecutive sisters *Habropetalum* and *Dioncophyllum* within *Dioncophyllaceae* are non-carnivorous (Rembold *et al*., 2010). However, the recent observation that phosphorus deficiency induced production of carnivorous sticky leaves may open a window for investigation of digestive physiology in this unusual plant (Winkelmann *et al*., 2023). Thus, *T. peltatum* remains an interesting object for the investigation of JA signalling in carnivorous plants.

Our study clearly showed that although carnivorous plants from different evolutionary lineages co-opted the same digestive enzymes (Fukushima *et al*., 2017), the regulation of their activities strongly differs (Supplementary Fig. S10). The carnivorous plants use JAs for their ancient role, defence, but the co-option of JA signalling for botanical carnivory has not occurred in all lineages of carnivorous plant. Although further studies are still needed, it seems that JA signalling has been co-opted for botanical carnivory only in the oldest order, *Caryophyllales*. Our experimental data are in agreement with the genome studies, where expansions of genes families involved in JA signalling were documented in *Droseraceae*, but not in *Cephalotus*, and even contraction was documented in aquatic *Utricularia* (Fukushima *et al*., 2017; Sun *et al*., 2018; Palfalvi *et al*., 2020). Thus, the constraints on the available routes to evolve plant carnivory are less than was previously thought.

## Supplementary data

The following supplementary data are available at *JXB* online.

Fig. S1. Extracellular recording of electrical signals in the withered pitcher of *Sarracenia purpurea* ssp. *venosa*.

Fig. S2. Phosphatase activities in the digestive fluid of carnivorous plants.

Fig. S3. Pigment content 20 d after 100 µM coronatine application in *Cephalotus follicularis*.

Fig. S4. Enzyme activities in response to 100 µM coronatine treatment in *Nepenthes* × *Mixta* plants.

Fig. S5. Immunodetection of type III chitinase in digestive fluid of *Sarracenia purpurea* ssp. *venosa* in response to feeding.

Fig. S6. Immunodetection of type III chitinase in the digestive fluid of *Sarracenia purpurea* ssp. *venosa* in response to coronatine application.

Fig. S7. Immunodetection of aspartic protease in digestive fluid during pitcher ontogeny in *Sarracenia purpurea* ssp. *venosa*.

Fig. S8. Immunodetection of type III chitinase in digestive fluid during pitcher ontogeny in *Sarracenia purpurea* ssp. *venosa*.

Fig. S9. Immunodetection of aspartic protease in digestive fluid during pitcher ontogeny in *Cephalotus follicularis*.

Fig. S10. Summary of enzyme activity regulation in studied carnivorous plants.

Table S1. List of MRM transitions used in quantitative analysis of phytohormones.

Table S2. Important characteristics for all identified proteins supplemented with functional annotations assigned by a pBLAST search in *Drosophyllum lusitanicum*.

Dataset S1. Original output pdf file from the Peaks X Pro software containing detailed information on protein and peptide identification characteristics.

erad359_suppl_Supplementary_Material

erad359_suppl_Supplementary_Table_S2

erad359_suppl_Supplementary_Data

## Data Availability

The data supporting the findings of this study are available within the paper and within its supplementary data published online.
