# Supplementary material for: Is the co-option of jasmonate signalling for botanical carnivory a universal trait for all carnivorous plants?
Source: J Exp Bot. 2023 Sep 14;75(1):334–49. doi: 10.1093/jxb/erad359 (PMC10735409; doi:10.1093/jxb/erad359)
Supplement: erad359_suppl_Supplementary_Material [file erad359_suppl_supplementary_material.pdf]

SUPPLEMENTARY DATA

Is the co-option of jasmonate signalling for botanical carnivory universal trait for all carnivorous plants?

Andrej Pavlovič[1*], Jana Koller[1], Ondřej Vrobel[2.3], Ivo Chamrád[4], René Lenobel[4], Peter Tarkowski[2.3]

[1] *Department of Biophysics, Faculty of Science, Palacký University, Šlechtitelů 27, CZ-783 71, Olomouc, Czech Republic.*

[2] *Center of the Region Haná for Biotechnological and Agricultural Research, Czech Advanced Technology and Research Institute, Palacký University, Šlechtitelů 27, CZ-783 71, Olomouc, Czech Republic.*

[3] *Center of the Region Haná for Biotechnological and Agricultural Research, Department of Genetic Resources for Vegetables, Medicinal and Special Plants, Crop Research Institute, Šlechtitelů 29, CZ-783 71, Olomouc, Czech Republic.*

[4] *Laboratory of Growth Regulators, Faculty of Science, Palacký University and Institute of Experimental Botany of the Czech Academy of Sciences, Šlechtitelů 27, CZ-783 71, Olomouc, Czech Republic.*

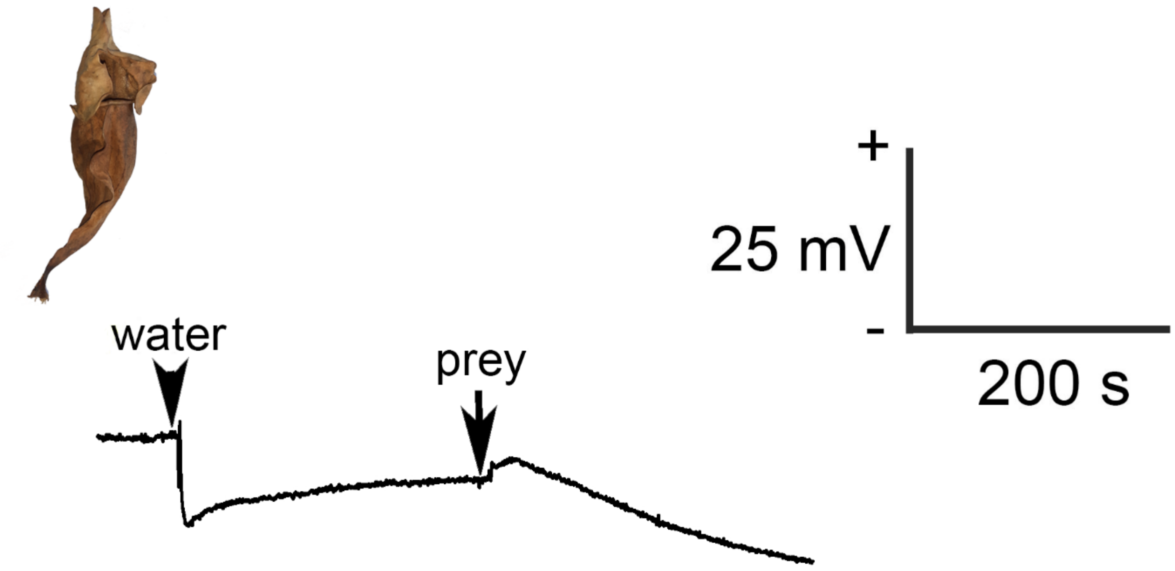

Fig. S1 Extracellular recording of electrical signals in the withered pitcher of *Sarracenia purpurea* ssp. *venosa*. Changes of membrane potential in response to water addition (arrowhead) and prey (arrow).

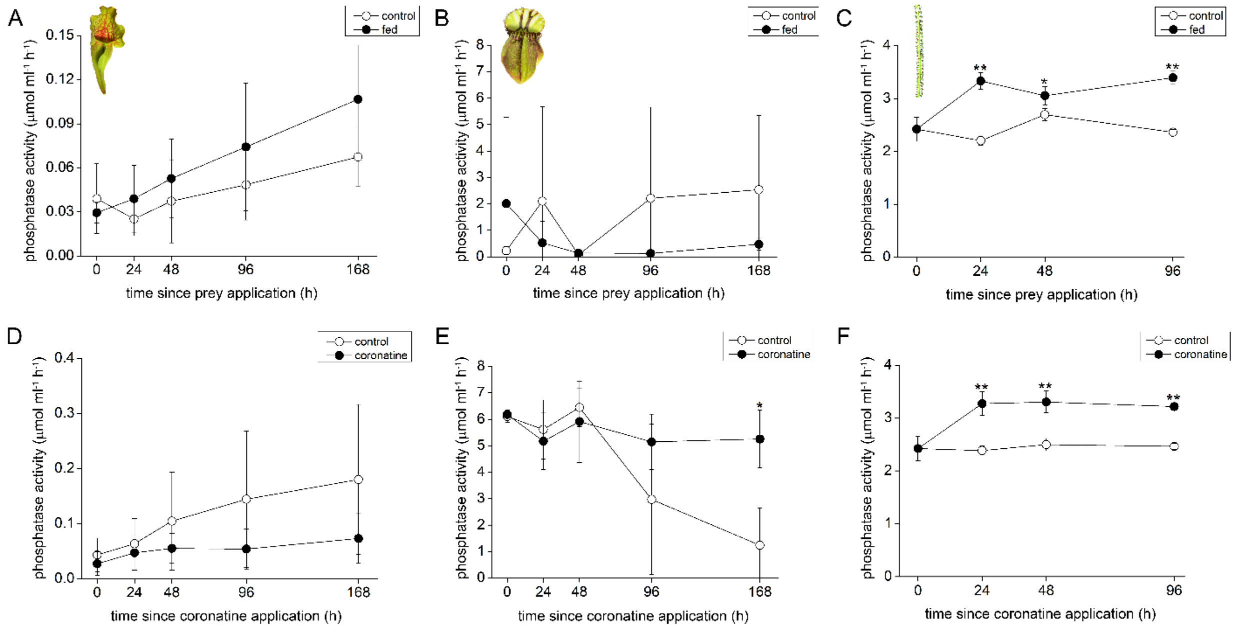

Fig. S2 Phosphatase activities in the digestive fluid of carnivorous plants. (A–C) feeding with insect prey; (D–F) coronatine application. (A, D) *Sarracenia purpurea* ssp. *venosa*; (B, E) *Cephalotus follicularis*; (C, F) *Drosophyllum lusitanicum*. Open circles (control plants); closed circles (fed or coronatine-treated plants). Data are means ± S.D., *n* = 4-8. Significant differences (Student's *t*-test) between the control and treated samples at the same time point are indicated, *P < 0.05 and **P < 0.01.

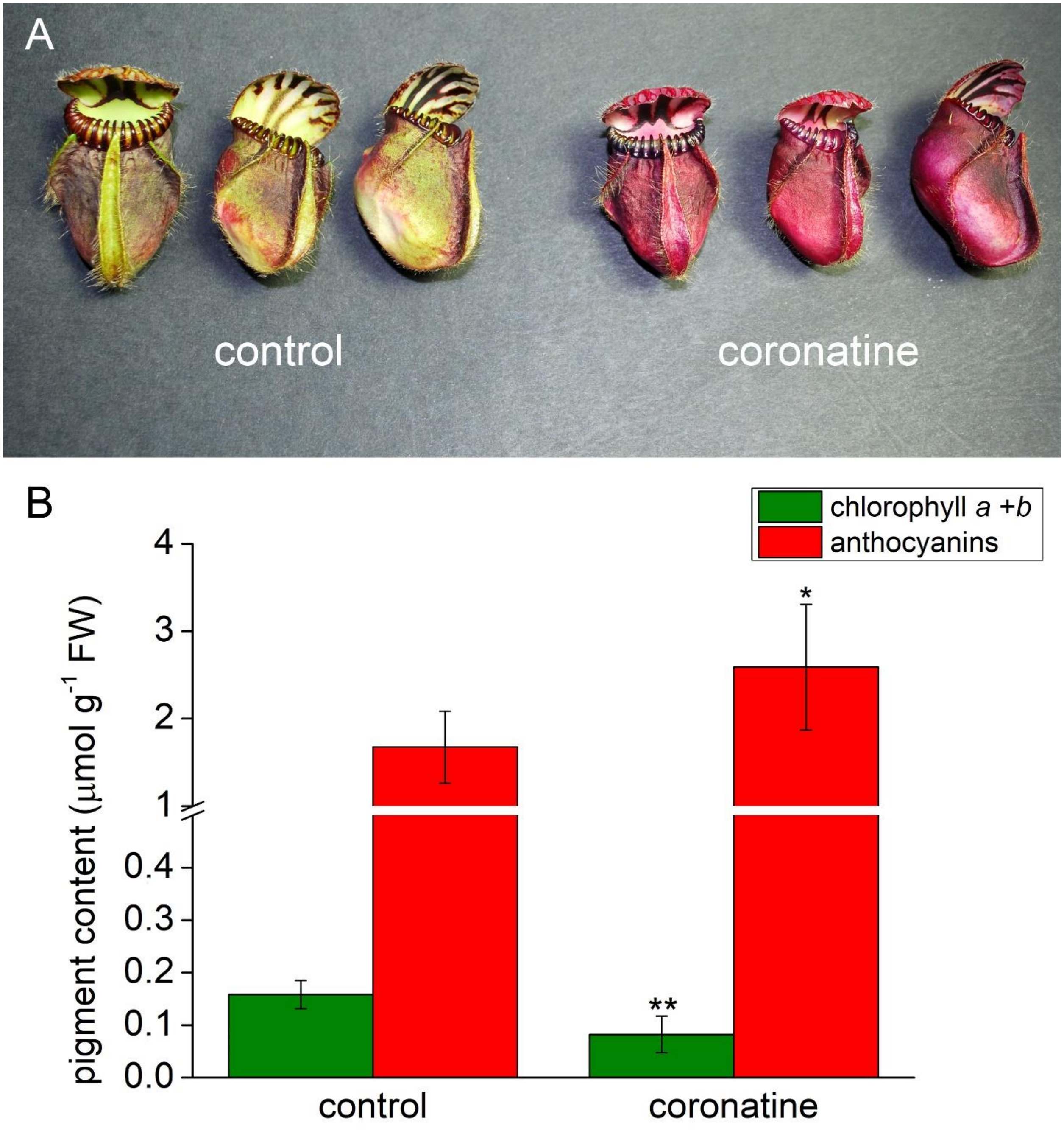

Fig. S3 Pigment content 20 d after 100 µM coronatine application in *Cephalotus follicularis*. (A) pitcher coloration; (B) pigment content. Data are means ± S.D., *n* = 4. Significant differences (Student's *t*-test) between control and coronatine-treated plants are indicated, *P < 0.05 and **P < 0.01.

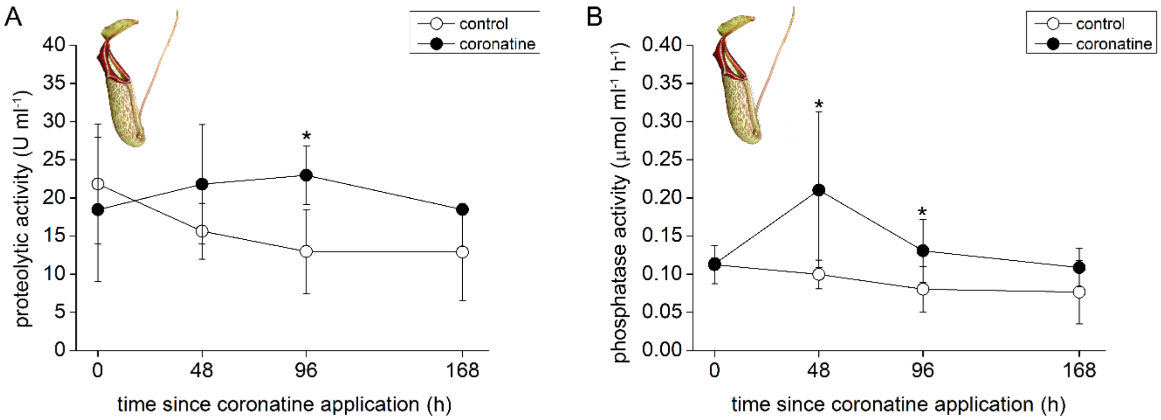

Fig. S4 Enzyme activities in response to 100 µM coronatine treatment in *Nepenthes* x *Mixta* plants. (A) proteolytic activity, (B) phosphatase activity. Open circles (control plants); closed circles (coronatine-treated plants). Data are means ± S.D., *n* = 6-9. Significant differences (Student's *t*-test) between the control and treated samples at the same time point are indicated, *P < 0.05.

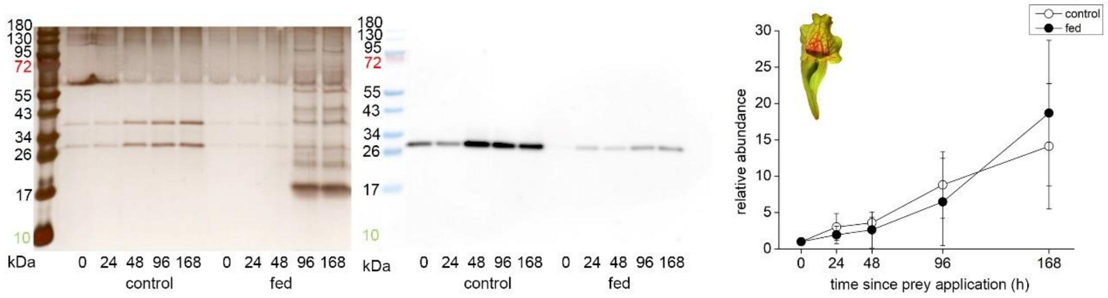

Fig. S5 Immunodetection of type III chitinase in the digestive fluid of *Sarracenia purpurea* ssp. *venosa* in response to feeding. The proteins were separated in 10% (v/v) sodium dodecyl sulphate–polyacrylamide gel electrophoresis (SDS-PAGE) and silver stained (left) or subjected to Western blot analysis (middle) and the chemiluminescence signal intensity was quantified (right). Signal intensity at zero time point was set up as 1. Open circles (control plants), closed circles (fed plants). Representative gel and blot are shown. Data are means ± S.D., $n = 4$. No significant differences between the control and treated samples at the same time points were found (Student's *t*-test). The protein marker in the image of immunodetected protein was added manually based on the merged image from the gel scanner. For immunodetection of aspartic protease in *S. purpurea* ssp. *venosa* from the same samples see Fig. 5A.

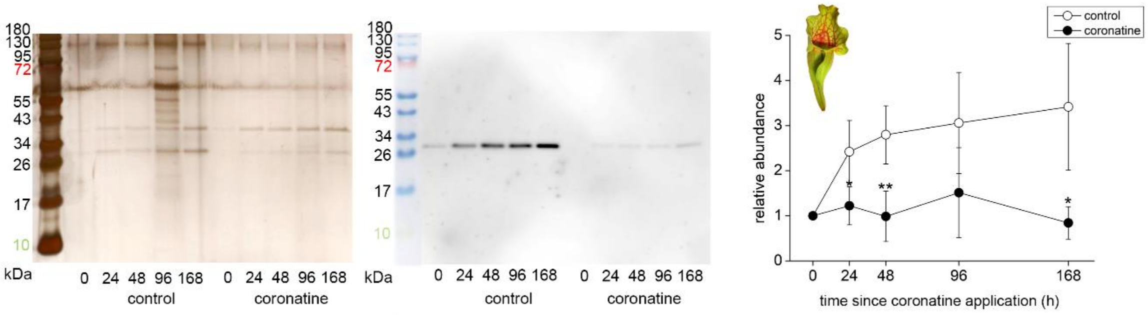

Fig. S6 Immunodetection of type III chitinase in the digestive fluid of *Sarracenia purpurea* ssp. *venosa* in response to coronatine application. The proteins were separated in 10% (v/v) sodium dodecyl sulphate–polyacrylamide gel electrophoresis (SDS-PAGE) and silver stained (left) or subjected to Western blot analysis (middle) and the chemiluminescence signal intensity was quantified (right). Signal intensity at zero time point was set up as 1. Open circles (control plants); closed circles (coronatine-treated plants). Representative gel and blot are shown. Data are means ± S.D., $n = 4$. Significant differences (Student's *t*-test) between the control and treated samples at the same time point are indicated, *P < 0.05 and **P < 0.01. The protein marker in the image of immunodetected protein was added manually based on the merged image from the gel scanner. For immunodetection of aspartic protease in *S. purpurea* ssp. *venosa* from the same samples see Fig. 6A.

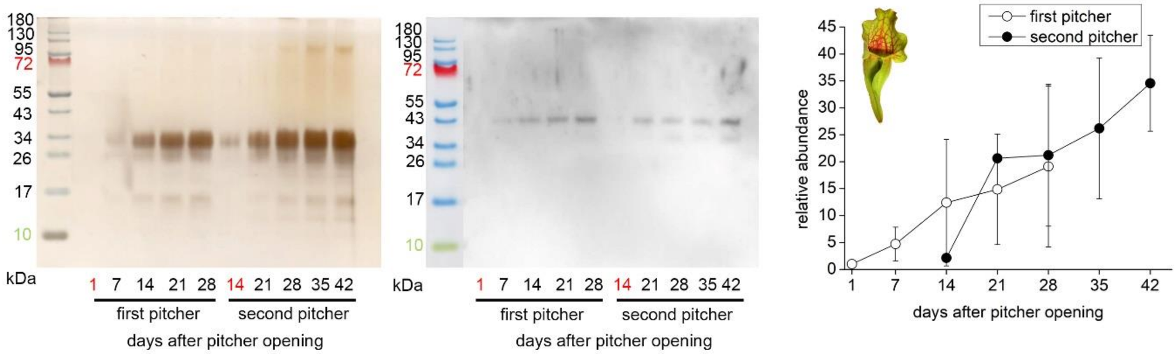

Fig. S7 Immunodetection of aspartic protease during pitcher ontogeny in *Sarracenia purpurea* ssp. *venosa*. The day the pitchers opened is marked as 1. In the first pitcher, 5 mL of distilled water was added on the same day of pitcher opening, in the second pitcher, the same volume was added on the 14th day after opening. The day of water addition is marked in red. The proteins were separated in 10% (v/v) sodium dodecyl sulphate–polyacrylamide gel electrophoresis (SDS-PAGE) and silver stained (left) or subjected to Western blot analysis (middle) and the chemiluminescence signal intensity was quantified (right). Both bands were used for quantification. The signal intensity of the first day was set up as 1. First pitcher (open circles), second pitcher (closed circles). Representative gel and blot are shown. Means ± S.D., n = 3. The protein marker in the image of immunodetected protein was added manually based on the merged image from the gel scanner.

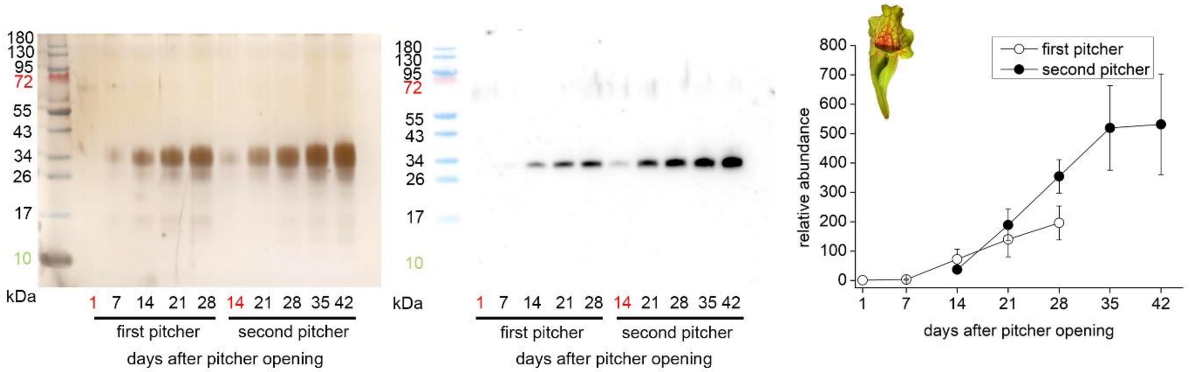

Fig. S8 Immunodetection of type III chitinase during pitcher ontogeny in *Sarracenia purpurea* ssp. *venosa*. The day the pitchers opened is marked as 1. In the first pitcher, 5 mL of distilled water was added on the same day of pitcher opening, in the second pitcher, the same volume was added on the 14th day after opening. The day of water addition is marked in red. The proteins were separated in 10% (v/v) sodium dodecyl sulphate–polyacrylamide gel electrophoresis (SDS-PAGE) and silver stained (left) or subjected to Western blot analysis (middle) and the chemiluminescence signal intensity was quantified (right). The signal intensity of first day was set up as 1. First pitcher (open circles), second pitcher (closed circles). Representative gel and blot are shown. Data are means ± S.D., $n = 4$. The protein marker in the image of immunodetected protein was added manually based on the merged image from the gel scanner.

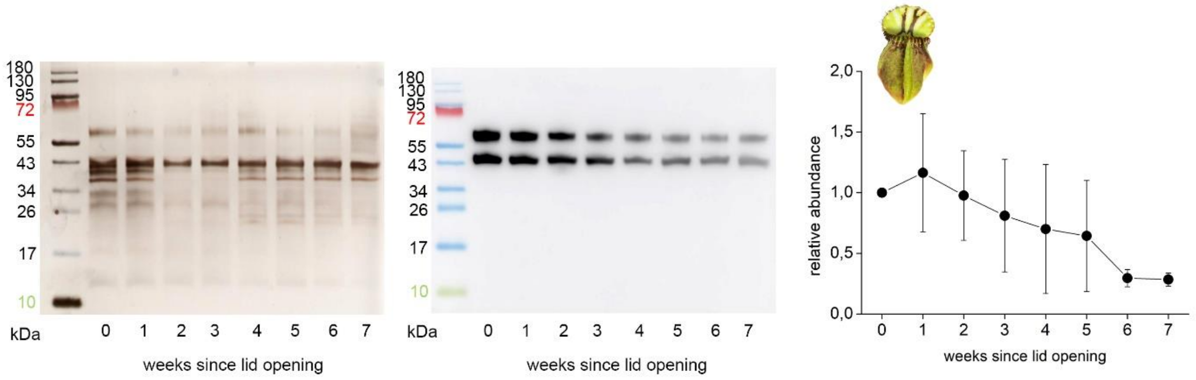

Fig. S9 Immunodetection of aspartic protease in digestive fluid during pitcher ontogeny in *Cephalotus follicularis*. The day the pitcher opened is marked as 0. The proteins were separated in 10% (v/v) sodium dodecyl sulphate–polyacrylamide gel electrophoresis (SDS-PAGE) and silver stained (left) or subjected to Western blot analysis (middle) and the chemiluminescence signal intensity was quantified (right). Both bands were used for quantification. Signal intensity at zero time point was set up as 1. Representative gel and blot are shown. Data are means ± S.D., $n = 4$. The protein marker in the image of immunodetected protein was added manually based on the merged image from the gel scanner.

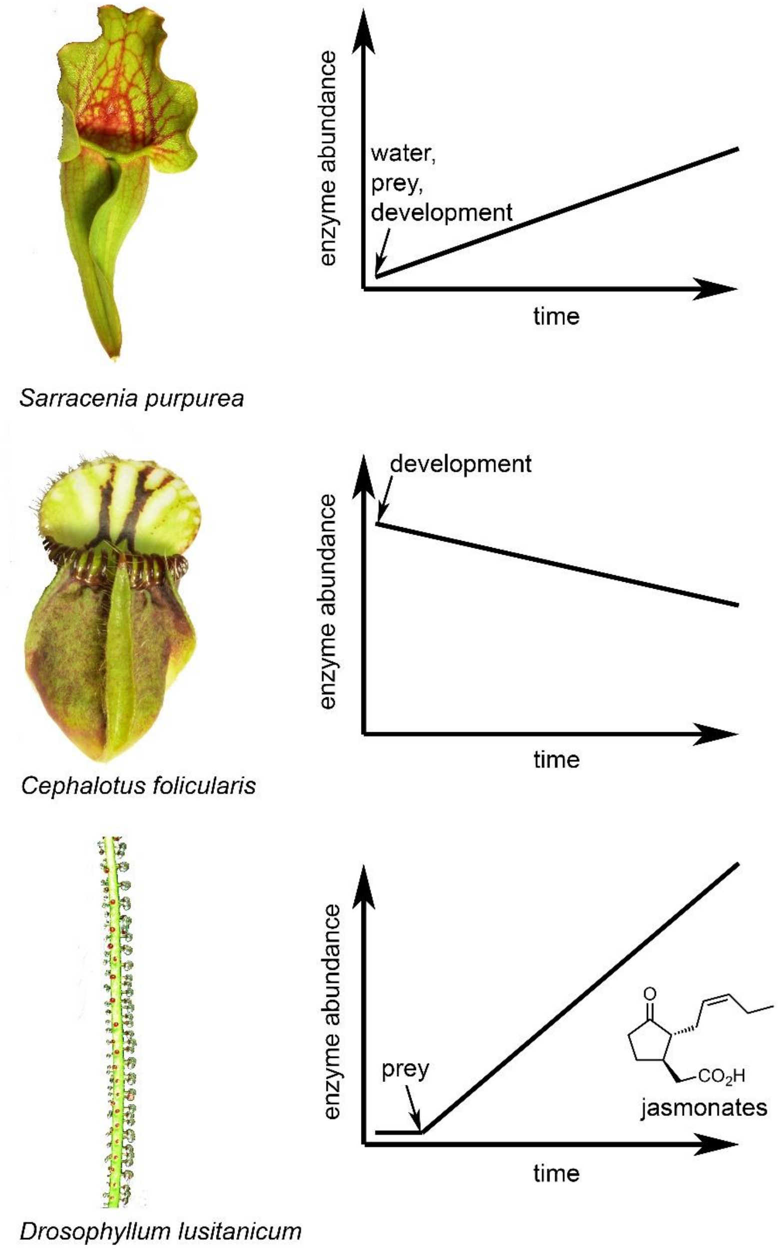

Fig. S10 Summary of enzyme activity regulation in studied carnivorous plants. In *Sarracenia purpurea ssp. venosa* enzyme activity is upregulated developmentally, by water and prey addition. In *Cephalotus follicularis,* the enzyme activity is rather constitutive or regulated developmentaly and decreases with pitcher ontogeny. In *Drosophyllum lusitanicum,* the enzyme activity is regulated by insect prey through jasmonate signaling.

Table S1: List of MRM transitions used in quantitative analysis of phytohormones. Transitions are listed in format Q1 Mass>Q3 Mass (Collision Energy in eV).

Analyte	ESI Mode	MRM 1	MRM 2	MRM 3
OPDA	(-)	291.30>165.35 (20)	291.30>247.40 (19)	291.30>273.35 (18)
JA	(-)	209.35>59.00 (14)	209.35>40.90 (37)	
JA-Ile	(+)	324.30>278.0 (-13)	324.30>151.20 (-15)	324.20>133.20 (-19)
ABA	(-)	263.20>153.20 (13)	263.20>204.20 (21)	263.20>219.20 (14)
SA	(-)	137.20>93.05 (21)	137.20>65.00 (29)	
IAA	(+)	176.00>130.05 (-16)	176.00>77.20 (-42)	176.00>103.15 (-32)
Internal Standards		MRM 1	MRM 2	MRM 3
D5-OPDA	(-)	296.20>170.45 (21)	296.20>252.45 (17)	296.20>278.40 (17)
D5-JA	(-)	214.30>61.95 (13)	214.30>42.05 (40)	
D2-JA-Ile	(+)	326.30>280.00 (-13)	326.30>151.2 (-15)	326.30>133.20 (-19)
D6-ABA	(-)	269.25>159.25 (12)	269.25>225.25 (15)	269.25>207.20 (22)
D4-SA	(-)	141.20>97.10 (21)	141.20>69.10 (31)	
$^{13}C_6$-IAA	(+)	182.05>136.05 (-16)	182.05>109.10 (-31)	182.05>81.10 (-43)

Table S2 Important characteristics for all identified proteins supplemented with functional annotations assigned by a pBLAST search in *Drosophyllum lusitanicum*, (Excel file).

Data S1 Original output pdf file from the Peaks X Pro software containing detailed information on protein and peptide identification characteristics.
