# Supplementary material for: Is the co-option of jasmonate signalling for botanical carnivory a universal trait for all carnivorous plants?
Source: J Exp Bot. 2023 Sep 14;75(1):334–49. doi: 10.1093/jxb/erad359 (PMC10735409; doi:10.1093/jxb/erad359)
Supplement: erad359_suppl_Supplementary_Data [file erad359_suppl_supplementary_data.pdf]

Summary

1. Notes

2. Result Statistics

Figure 1. False discovery rate (FDR) curve. X axis is the number of peptides being kept. Y axis is the corresponding FDR.

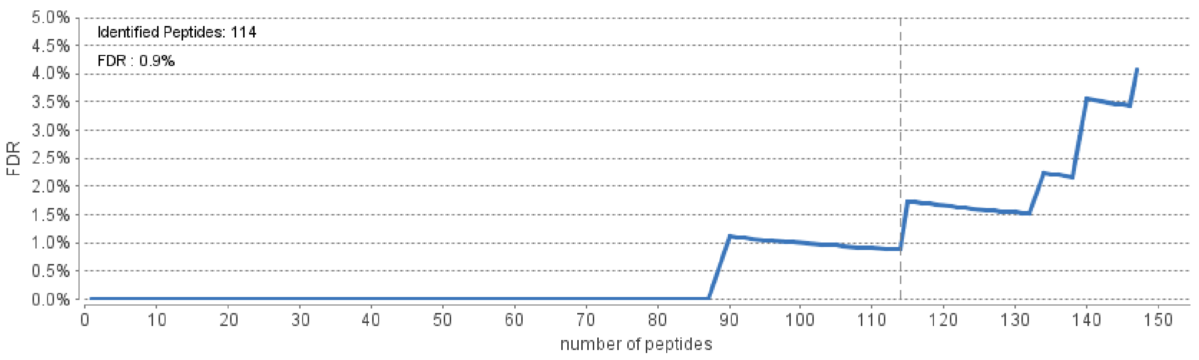

Figure 2. PSM score distribution. **(a)** Distribution of PEAKS peptide score; **(b)** Scatterplot of PEAKS peptide score versus precursor mass error.

(a) **(b)**

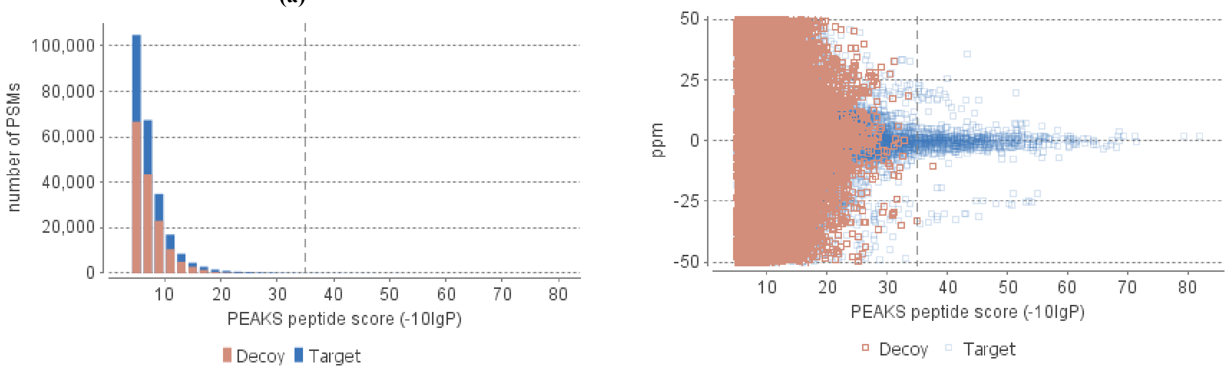

Table 1. Statistics of data.

	#Scans		#Features	Identified			#Peptides	#Sequences	#Proteins*		
	MS1	MS/MS		#PSMs	#Scans	#Features**			Groups	All	Top
Total	0	725845	0	646	646	0	112	77	45	384	305
AndrejP_JAtreatment	0	205135	0	174	174	0	38	31	27	174	132
AndrejP_Feeding	0	265916	0	206	206	0	49	36	25	215	156
AndrejP_CNTR	0	254794	0	266	266	0	80	59	36	290	211

* proteins with significant peptides are used in counts.
** features are identified by DB search only.

Figure 3. Sample overlap for Proteins and Peptides (up to 8 samples). **(a)** All Proteins; **(b)** Top Proteins; **(c)** Peptides;

(a) **(b)** **(c)**

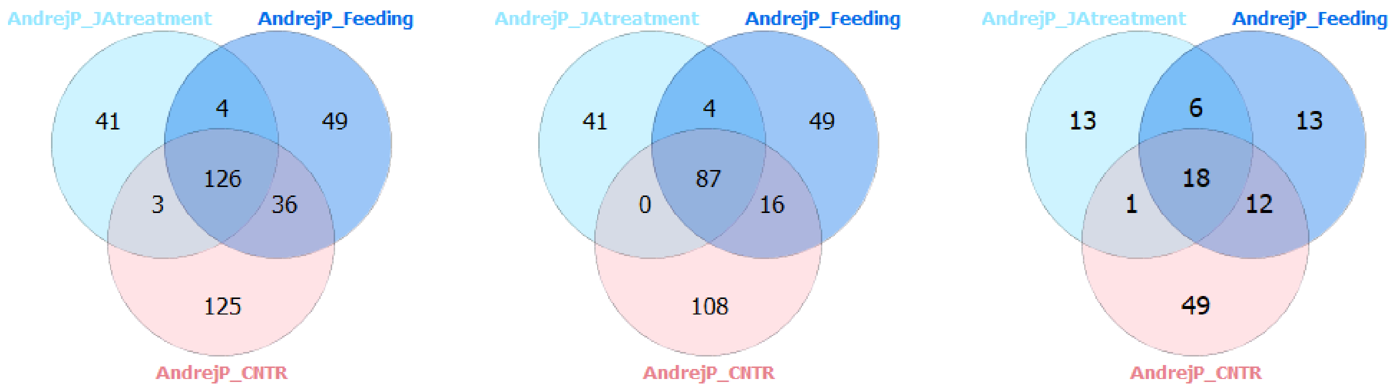

Figure 4. Distribution of peptide feature detection. **(a)** Feature m/z distribution; **(b)** Feature RT distribution.

(a)
No available data for figure

(b)
No available data for figure

Figure 5. Distribution of identified peptide features. **(a)** Feature abundance distribution; **(b)** *De novo* sequencing validation.

(a)
No available data for figure

(b)

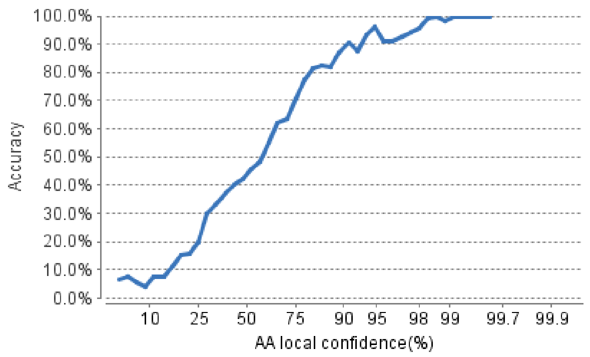

Table 2. Result filtration parameters.

Peptide -10lgP	≥34.9
PTM Ascore	≥100
Peptide mutation ion intensity	≥0%
Protein Group FDR	5.0%
Proteins unique peptides	≥1
De novo score(%)	≥70%

Table 3. Statistics of filtered result.

FDR (Peptide-Spectrum Matches)	0.2%
FDR (Peptide Sequences)	0.9%
FDR (Protein Group)	2.2%
De Novo Only Spectra	93380

Table 4. PTM profile.

Name	ΔMass	Position	#PSM	-10lgP	Abundance	AScore
Carbamidomethyl	57.02	C	95	53.85		1000.00
Oxidation	15.99	M	53	52.24		82.63
Methylation(KR)	14.02	KR	52	61.22		1000.00
Deamidation	.98	NQ	41	54.99		1000.00
Methylation(others)	14.02	DEH	22	54.00		8.69
Formylation	27.99	K,N-term	11	53.16		129.22
Kynurenin	3.99	W	6	55.22		1000.00
Acetylation	42.01	K	6	43.02		1000.00
Propionamide	71.04	N-term	5	64.80		1000.00
Guanidination	42.02	K	4	44.39		1000.00

3. Experiment Control

Figure 6. Precursor mass error of peptide-spectrum matches (PSM) in filtered result. **(a)** Distribution of precursor mass error in ppm; **(b)** Scatterplot of precursor m/z versus precursor mass error in ppm.

(a)

(b)

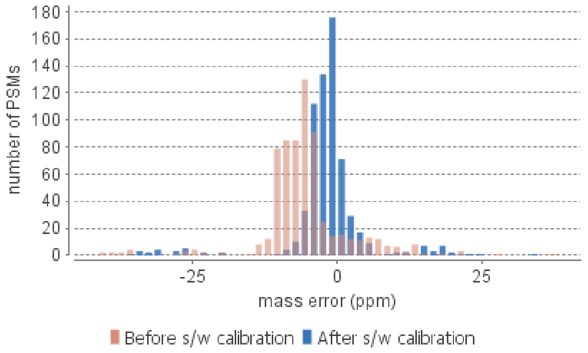

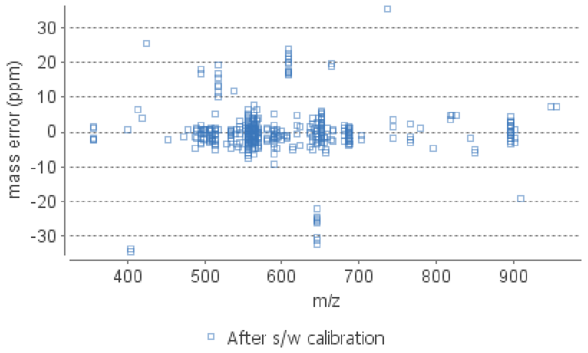

Table 5. Number of identified peptides in each sample by the number of missed cleavages.

Missed Cleavages	0	1	2	3	4+
AndrejP_JAtreatment	35	3	0	0	0
AndrejP_Feeding	43	6	0	0	0
AndrejP_CNTR	74	5	1	0	0

4. Other Information

Table 6. Search parameters.

Query Type: Homology Match
Fixed Modifications:
  Carbamidomethylation: 57.02
Variable Modifications:
  Acetylation (Protein N-term): 42.01
  Deamidation (NQ): 0.98
  Oxidation (M): 15.99
Fragment ion tolerance: 0.05
L equals I: true
Q equals K: true
Report number: 1
Maximum # of PTMs: 3
De novo score(%) threshold: 15
Peptide hit threshold (-10logP): 30.0
Peaks run ID: 69
Merge Options: no merge
Precursor Options: corrected
Charge Options: no correction
Filter Charge: 2 - 4
Process: true
Associate chimera: yes

Table 7. Instrument parameters.

Fractions: MAX09850_AndrejP_gel_SA01_BA1_01_19048.mgf, MAX09850
_AndrejP_gel_SA01_BA1_01_19049.mgf, MAX09851_AndrejP_gel_SA02
_BA2_01_19050.mgf, MAX09851_AndrejP_gel_SA02_BA2_01_19051.mgf
, MAX09852_AndrejP_gel_SA03_BA3_01_19052.mgf, MAX09852_Andrej
P_gel_SA03_BA3_01_19053.mgf, MAX09853_AndrejP_gel_SA04_BA4_01
_19054.mgf, MAX09853_AndrejP_gel_SA04_BA4_01_19055.mgf, MAX09
854_AndrejP_gel_SA05_BA5_01_19056.mgf, MAX09854_AndrejP_gel_S
A05_BA5_01_19057.mgf, MAX09855_AndrejP_gel_SA06_BA6_01_19058.
mgf, MAX09855_AndrejP_gel_SA06_BA6_01_19059.mgf, MAX09856_And
rejP_gel_SA07_BA7_01_19060.mgf, MAX09856_AndrejP_gel_SA07_BA7
_01_19061.mgf, MAX09857_AndrejP_gel_SA08_BA8_01_19062.mgf, MA
X09857_AndrejP_gel_SA08_BA8_01_19063.mgf, MAX09858_AndrejP_ge
l_SA09_BB1_01_19064.mgf, MAX09858_AndrejP_gel_SA09_BB1_01_190
65.mgf, MAX09859_AndrejP_gel_SA10_BB2_01_19066.mgf, MAX09859_
AndrejP_gel_SA10_BB2_01_19067.mgf, MAX09860_AndrejP_gel_SA11_
BB3_01_19068.mgf, MAX09860_AndrejP_gel_SA11_BB3_01_19069.mgf,
MAX09897_AndrejP_gel_F01_RA1_01_19173.mgf, MAX09897_AndrejP_
gel_F01_RA1_01_19174.mgf, MAX09898_AndrejP_gel_F02_RA2_01_191
75.mgf, MAX09898_AndrejP_gel_F02_RA2_01_19176.mgf, MAX09899_A
ndrejP_gel_F03_RA3_01_19177.mgf, MAX09899_AndrejP_gel_F03_RA3
_01_19178.mgf, MAX09900_AndrejP_gel_F04_RA4_01_19179.mgf, MAX
09900_AndrejP_gel_F04_RA4_01_19180.mgf, MAX09901_AndrejP_gel_
F05_RA5_01_19181.mgf, MAX09901_AndrejP_gel_F05_RA5_01_19182.m
gf, MAX09902_AndrejP_gel_F06_RA6_01_19183.mgf, MAX09902_Andre
jP_gel_F06_RA6_01_19184.mgf, MAX09903_AndrejP_gel_F07_RA7_01_
19185.mgf, MAX09903_AndrejP_gel_F07_RA7_01_19186.mgf, MAX0990
4_AndrejP_gel_F08_RA8_01_19187.mgf, MAX09904_AndrejP_gel_F08_
RA8_01_19188.mgf, MAX09905_AndrejP_gel_F09_RB1_01_19189.mgf,
MAX09905_AndrejP_gel_F09_RB1_01_19190.mgf, MAX09906_AndrejP_g
el_F10_RB2_01_19191.mgf, MAX09906_AndrejP_gel_F10_RB2_01_1919
2.mgf, MAX09907_AndrejP_gel_F11_RB3_01_19193.mgf, MAX09907_An
drejP_gel_F11_RB3_01_19194.mgf, MAX09908_AndrejP_gel_C01_RC1_
01_19198.mgf, MAX09908_AndrejP_gel_C01_RC1_01_19199.mgf, MAX0
9909_AndrejP_gel_C02_RC2_01_19200.mgf, MAX09909_AndrejP_gel_C
02_RC2_01_19201.mgf, MAX09910_AndrejP_gel_C03_RC3_01_19202.mg
f, MAX09910_AndrejP_gel_C03_RC3_01_19203.mgf, MAX09911_Andrej
P_gel_C04_RC4_01_19204.mgf, MAX09911_AndrejP_gel_C04_RC4_01_1
9205.mgf, MAX09912_AndrejP_gel_C05_RC5_01_19206.mgf, MAX09912
_AndrejP_gel_C05_RC5_01_19207.mgf, MAX09913_AndrejP_gel_C06_R
C6_01_19208.mgf, MAX09913_AndrejP_gel_C06_RC6_01_19209.mgf, M
AX09914_AndrejP_gel_C07_RC7_01_19210.mgf, MAX09914_AndrejP_ge
l_C07_RC7_01_19211.mgf, MAX09915_AndrejP_gel_C08_RC8_01_19212
.mgf, MAX09915_AndrejP_gel_C08_RC8_01_19213.mgf, MAX09916_And
rejP_gel_C09_RD1_01_19214.mgf, MAX09916_AndrejP_gel_C09_RD1_0
1_19215.mgf, MAX09917_AndrejP_gel_C10_RD2_01_19216.mgf, MAX09
917_AndrejP_gel_C10_RD2_01_19217.mgf, MAX09918_AndrejP_gel_C1
1_RD3_01_19218.mgf, MAX09918_AndrejP_gel_C11_RD3_01_19219.mgf

Ion Source: ESI(nano-spray)
Fragmentation Mode: CID, CAD(y and b ions)
MS Scan Mode: Quadrupole
MS/MS Scan Mode: Time of Flight (TOF)

Protein List

Protein Accession Contains:
Protein Description Contains:
Protein Sample Area >=
Protein PTM Contains:
Protein Mutations: False

Protein Group	Protein ID	Accession	-10lgP	Coverage (%)	Coverage (%) AndrejP_JAtreatment	Coverage (%) AndrejP_Feeding	Coverage (%) AndrejP_CNTR	Area AndrejP_JAtreatment	Area AndrejP_Feeding	Area AndrejP_CNTR	#Peptides	#Unique	#Spec AndrejP_JAtreatment	#Spec AndrejP_Feeding	#Spec AndrejP_CNTR	PTM	Avg. Mass	Description
**1**	1	APO15836.1	190.73	35	12	12	35	0	0	0	9	2	26	52	80	Y	41671	actin 11 [Sesuvium portulacastrum]
**3**	8	ASZ85168.1	167.97	28	8	8	28			0	9	1	21	26	69	Y	41726	actin 7 [Hylocereus polyrhizus]
**4**	21	QDK54754.1	156.47	26	8	8	26			0	8	1	21	26	66	Y	41637	actin [Polygonum cuspidatum]
**5**	42	BAW35430.1	123.96	8	5	8	2	0	0		5	2	52	28	3	Y	70511	purple acid phosphatase [Nepenthes alata]
**6**	94	KAH9619639.1	113.12	9	4	9	5		0	0	4	1	38	20	7	Y	69053	hypothetical protein [Heliosperma pusillum]
**7**	273	MCK0600380.1	110.35	13	13	13	13	0	0	0	2	1	35	20	6	Y	14338	acid phosphatase [Opuntia streptacantha]
**17**	82	XP_010695592.2	100.60	9	4	4	9	0	0	0	2	2	4	3	7	Y	28976	uncharacterized protein LOC104908202 [Beta vulgaris subsp. vulgaris]
**25**	69	KMT05278.1	93.61	5	0	0	5			0	3	1	0	0	7	Y	71296	hypothetical protein isoform B [Beta vulgaris subsp. vulgaris]
**15**	198	MCK0637628.1	91.93	3	3	3	3	0	0	0	2	2	7	13	11	Y	43906	hypothetical protein [Opuntia streptacantha]
**12**	68	ASZ85176.1	89.45	7	4	2	5			0	3	1	9	2	8	N	49870	elongation factor 1-alpha [Hylocereus polyrhizus]
**21**	138	XP_021841864.1	84.86	4	0	0	4			0	1	1	0	0	10	N	38488	fructose-bisphosphate aldolase 5, cytosolic [Spinacia oleracea]
**13**	47	KAH9613912.1	84.71	6	4	2	5			0	3	1	9	2	9	N	49331	hypothetical protein [Heliosperma pusillum]
**31**	174	XP_021760870.1	83.34	3	0	0	3			0	2	2	0	0	3	N	70384	heat shock cognate 70 kDa protein-like [Chenopodium quinoa]
**26**	44	KAH9602483.1	83.29	6	0	0	6			0	3	1	0	0	6	N	70976	hypothetical protein [Heliosperma pusillum]
**16**	134	KAH9624815.1	81.53	6	5	6	2	0	0	0	3	3	6	11	3	Y	55985	hypothetical protein [Heliosperma pusillum]
**29**	192	UGZ35775.1	80.01	8	0	0	8			0	2	2	0	0	6	Y	48164	enolase [Phytolacca americana]
**14**	157	KNA11181.1	75.76	9	4	9	4	0	0	0	2	2	3	10	11	N	27925	hypothetical protein [Spinacia oleracea]
**22**	121	XP_021852453.1	73.66	6	2	6	2		0	0	3	1	3	3	1	Y	71417	probable inactive purple acid phosphatase 27 [Spinacia oleracea]
**9**	130	XP_048495078.1	72.37	5	2	5	5	0	0	0	2	2	6	20	15	Y	46582	alpha-galactosidase 1 isoform X1 [Beta vulgaris subsp. vulgaris]
**8**	113	MCK0622870.1	69.82	6	6	2	6	0	0	0	2	2	17	15	19	Y	40003	hypothetical protein [Opuntia streptacantha]
**30**	227	XP_021719798.1	68.69	10	0	10	10		0	0	1	1	0	4	2	N	13008	cysteine proteinase inhibitor 1-like [Chenopodium quinoa]
**23**	146	XP_021769989.1	61.94	12	0	12	12		0	0	2	2	0	3	4	Y	20593	ADP-ribosylation factor 1-like [Chenopodium quinoa]
**19**	194	XP_021859482.1	54.99	2	2	2	0	0	0		1	1	13	5	0	Y	55310	serine carboxypeptidase II-3-like [Spinacia oleracea]
**10**	189	XP_021758194.1	53.12	2	2	2	2	0	0	0	1	1	16	13	12	Y	56353	alpha-galactosidase 3-like [Chenopodium quinoa]
**61**	281	MCK0601578.1	51.57	5	0	0	5			0	1	1	0	0	1	Y	26757	hypothetical protein [Opuntia streptacantha]
**36**	278	XP_021743448.1	50.75	4	4	4	4	0	0	0	1	1	1	1	1	N	57245	probable glucan 1,3-beta-glucosidase A [Chenopodium quinoa]
**62**	359	BAM28610.1	48.19	4	0	0	4			0	1	1	0	0	1	Y	31004	class III chitinase [Nepenthes alata]
**57**	222	XP_021730180.1	45.74	2	0	0	2			0	1	1	0	0	1	N	69051	phosphoglucomutase, chloroplastic-like [Chenopodium quinoa]
**47**	848	APO15851.1	43.42	2	2	0	0	0			1	1	1	0	0	Y	129217	RNA-dependent RNA polymerase 1 [Sesuvium portulacastrum]
**24**	171	KNA06751.1	42.86	3	3	3	3	0	0	0	1	1	1	1	2	Y	45065	hypothetical protein [Spinacia oleracea]
**65**	377	XP_021867395.1	41.83	2	2	0	0	0			1	1	1	0	0	N	40979	GDSL esterase/lipase 7-like [Spinacia oleracea]
**42**	237	KAH9604880.1	41.15	3	0	0	3			0	1	1	0	0	1	Y	46991	hypothetical protein [Heliosperma pusillum]
**40**	458	ABB89525.1	39.83	3	3	0	0	0			1	1	2	0	0	N	36577	glucanase [Nepenthes khasiana]
**55**	481	YP_009944427.1	38.94	3	3	0	0	0			1	1	1	0	0	Y	53772	ATP synthase CF1 beta subunit [Nepenthes khasiana]
**18**	367	XP_010685437.1	38.88	2	2	2	2	0	0	0	1	1	1	7	4	Y	50845	aluminum-activated malate transporter 2 [Beta vulgaris subsp. vulgaris]
**32**	358	XP_021743893.1	37.18	6	0	0	6			0	1	1	0	0	1	N	15141	histone H2A-beta, sperm-like [Chenopodium quinoa]
**64**	399	AAQ75601.1	37.16	2	2	0	0	0			1	1	1	0	0	Y	49577	ribulose-1,5-bisphosphate carboxylase/oxygenase large subunit, partial [Alternanthera caracasana]
**37**	364	XP_021774585.1	36.98	1	1	0	0	0			1	1	1	0	0	N	127851	uncharacterized protein LOC110738482 [Chenopodium quinoa]
**20**	521	XP_021761383.1	36.74	3	3	3	3	0	0	0	1	1	3	1	1	Y	32915	basic endochitinase-like [Chenopodium quinoa]
**51**	1940	XP_010669985.1	36.74	4	0	0	4			0	1	1	0	0	1	Y	42489	phosphoglycerate kinase 3, cytosolic [Beta vulgaris subsp. vulgaris]
**11**	380	XP_021838555.1	36.16	4	0	4	0		0		1	1	0	2	0	N	42329	uncharacterized protein LOC110778291 [Spinacia oleracea]
**45**	546	KAH9622019.1	35.74	7	0	0	7			0	1	1	0	0	1	N	16294	hypothetical protein [Heliosperma pusillum]
**60**	4248	XP_010676513.2	35.12	5	0	0	5			0	1	1	0	0	1	Y	24074	protein P21 [Beta vulgaris subsp. vulgaris]
**43**	454	XP_021861922.1	35.09	4	0	4	0		0		1	1	0	1	0	N	28382	14-3-3 protein 1-like [Spinacia oleracea]
**59**	384	XP_010690445.1	34.95	2	0	0	2			0	1	1	0	0	1	N	34326	triosephosphate isomerase, chloroplastic [Beta vulgaris subsp. vulgaris]
**total 45 proteins**																		

APO15836.1

back to list

| **Protein Coverage** | **Supporting Peptides** |

Protein Coverage:

1 MCDDVQALVI DNGSGMCKAG FAGDDAPRAV FPSIVGRPRH TGVMVGMGQK DSYVGDEAQS KRGILTLKYP IEHGIVTNWD

81 DMEKIWHHTF YNELRVAPEE HPVLLTEAPL NPKANREKMT QIMFETFNTP AMYVAIQAVL SLYASGRTTG IVMDSGDGVT

161 HTVPIYEGYA LPHAILRLDL AGRDLTDYLM KILTERGYSF TTTAEREIVR DIKEKLAYVA LDFEQEMQTA ASSSSLEKSY

241 ELPDGQVITI GNERFRCPEA LFQPSFLGME AAGVHETTYN SIMKCDVDIR KDLYGNVVLS GGSTMFPGIA DRMQKELTAL

321 APSTIKIKII APPERKYSVW IGGSILASLS TFQQMWISKE EYDESGPSIV HRKCF

d Dimethylation(KR) (+28.03)
o Oxidation (M) (+15.99)
t Tryptophan oxidation to kynurenin (+3.99)

Supporting Peptides:

Peptide	Uniq	-10lgP	Mass	Length	ppm	m/z	z	RT	Fraction	Scan	Source File	Area AndrejP_JAtreatment	Area AndrejP_Feeding	Area AndrejP_CNTR	#Feature	#Feature AndrejP_JAtreatment	#Feature AndrejP_Feeding	#Feature AndrejP_CNTR	Start	End	PTM	AScore	Found By
K.SYELPDGQVITIGNER.F	Y	82.03	1789.8846	16	1.8	895.9473	2	51.40	56	F56:12017	MAX09913_AndrejP_gel_C06_RC6_01_19209.mgf	0	0	0	0	0	0	0	239	254			PEAKS DB
R.VAPEEHPVLLTEAPLNPK.A	N	63.31	1953.0570	18	0.8	652.0269	3	30.71	20	F20:6520	MAX09859_AndrejP_gel_SA10_BB2_01_19067.mgf	0	0	0	0	0	0	0	96	113			PEAKS DB
K.IWHHTFYNELR.V	N	60.98	1514.7418	11	0.8	505.9194	3	28.89	56	F56:6292	MAX09913_AndrejP_gel_C06_RC6_01_19209.mgf			0	0	0	0	0	85	95			PEAKS DB
R.GYSFTTTAER.E	N	57.64	1131.5197	10	-1.6	566.7632	2	20.19	24	F24:4020	MAX09897_AndrejP_gel_F01_RA1_01_19174.mgf	0	0	0	0	0	0	0	197	206			PEAKS DB
K.YPIEH(+14.02)GIVTNWDDMEK.I	N	54.00	1959.9037	16	-1.6	654.3031	3	46.39	65	F65:11004	MAX09918_AndrejP_gel_C11_RD3_01_19218.mgf			0	0	0	0	0	69	84		H5:Methylation(others): 8.69	PEAKS PTM
K.AGFAGDDAPR.A	N	52.50	975.4410	10	0.6	488.7260	2	13.73	56	F56:2255	MAX09913_AndrejP_gel_C06_RC6_01_19209.mgf			0	0	0	0	0	19	28			PEAKS DB
K.YPIE(+14.02)HGIVTNWDDMEK.I	N	52.33	1959.9037	16	-1.9	654.3031	3	47.00	66	F66:11177	MAX09918_AndrejP_gel_C11_RD3_01_19219.mgf			0	0	0	0	0	69	84		E4:Methylation(others): 0.00	PEAKS PTM
K.Y(+27.99)PIEHGIVTNWDDM(+15.99)EK.I	N	51.45	1989.8778	16	19.7	664.3101	3	42.36	56	F56:9716	MAX09913_AndrejP_gel_C06_RC6_01_19209.mgf			0	0	0	0	0	69	84	Oxidation (M)	Y1:Formylation:78.75;M14:Oxidation (M):1000.00	PEAKS PTM
R.TTGIVMDSGDGVTHTVPIYEGYALPHAILR.L	Y	46.58	3182.6069	30	-4.7	796.6502	4	56.17	66	F66:13688	MAX09918_AndrejP_gel_C11_RD3_01_19219.mgf			0	0	0	0	0	148	177			PEAKS DB
K.IW(+31.99)HHTFYNELR.V	N	45.11	1546.7317	11	0.6	516.5826	3	28.63	55	F55:6246	MAX09913_AndrejP_gel_C06_RC6_01_19208.mgf			0	0	0	0	0	85	95		W2:Dihydroxy:52.86	PEAKS PTM
R.AVFPSIVGRPR.H	N	42.33	1197.6981	11	0.4	599.8540	2	32.95	55	F55:7328	MAX09913_AndrejP_gel_C06_RC6_01_19208.mgf			0	0	0	0	0	29	39			PEAKS DB
K.YPIE(+28.03)HGIVTNWDDMEK.I	N	42.21	1973.9193	16	1.1	658.9783	3	50.48	56	F56:11779	MAX09913_AndrejP_gel_C06_RC6_01_19209.mgf			0	0	0	0	0	69	84		E4:Ethylation:12.33	PEAKS PTM
R.DLTDYLM(+15.99)K.I	N	39.82	1013.4739	8	1.0	507.7426	2	33.90	56	F56:7492	MAX09913_AndrejP_gel_C06_RC6_01_19209.mgf			0	0	0	0	0	184	191	Oxidation (M)	M7:Oxidation (M):1000.00	PEAKS DB
K.SYELPDGQVITIGNER(+28.03).F	Y	39.08	1817.9159	16	-19.2	909.9438	2	52.28	55	F55:12278	MAX09913_AndrejP_gel_C06_RC6_01_19208.mgf			0	0	0	0	0	239	254	Dimethylation(KR)	R16:Dimethylation(KR): 1000.00	PEAKS PTM
R.DLTDYLMK.I	N	37.91	997.4790	8	0.6	499.7449	2	45.47	56	F56:10521	MAX09913_AndrejP_gel_C06_RC6_01_19209.mgf			0	0	0	0	0	184	191			PEAKS DB
K.IW(+3.99)HHTFYNELR.V	N	35.86	1518.7368	11	0.5	507.2509	3	28.33	56	F56:6298	MAX09913_AndrejP_gel_C06_RC6_01_19209.mgf			0	0	0	0	0	85	95	Tryptophan oxidation to kynurenin	W2:Tryptophan oxidation to kynurenin:1000.00	PEAKS PTM
**total 16 peptides**																							

ASZ85168.1

back to list

| Protein Coverage | Supporting Peptides |

Protein Coverage:

1 MADAEEIQPL VCDNGTGMVK AGFAGDDAPR AVFPSIVGRP RHTGVMVGMG QKDAYVGDEA QSKRGILTLK YPIEHGIVSN

81 WDDMEKIWHH TFYNELRVAP EEHPVLLTEA PLNPKANREK MTQIMFETFN VPAMYVAIQA VLSLYASGRT TGIVLDSGDG

161 VSHTVPIYEG YALPHAILRL DLAGRDLTDY LMKILTERGY MFTTSAERE I VRDIKEKLAY VALDFEQESE TAKSSSAVEK

241 NYELPDGQVI TIGAERFRCP EVLFQPSLIG MEAAGIHETT YNSIMKCDVD IRKDLYGNIV LSGGTTMFPG IADRMSKEIT

321 ALAPSSMKIK VVAPPERKYS VWIGGSILAS LSTFQQMWIS KGEYDESGPS IVHRKCF

- a Acetylation (K) (+42.01)
- d Deamidation (NQ) (+0.98)
- m Methylation(KR) (+14.02)
- o Oxidation (M) (+15.99)
- p Propionamide (K, X@N-term) (+71.04)
- s Sulphone (+31.99)
- t Tryptophan oxidation to kynurenin (+3.99)
- T V->T

Supporting Peptides:

Peptide	Uniq	-10lgP	Mass	Length	ppm	m/z	z	RT	Fraction	Scan	Source File	Area AndrejP_JAtreatment	Area AndrejP_Feeding	Area AndrejP_CNTR	#Feature	#Feature AndrejP_JAtreatment	#Feature AndrejP_Feeding	#Feature AndrejP_CNTR	Start	End	PTM	AScore	Found By
K.G(+71.04)EYDESGPSIVHR.K	Y	64.80	1515.6953	13	-1.6	506.2350	3	17.24	66	F66:3207	MAX09918_AndrejP_gel_C11_RD3_01_19219.mgf			0	0	0	0	0	362	374	Propionamide (K, X@N-term)	G1:Propionamide (K, X@N-term):1000.00	PEAKS PTM
R.VAPEEHPVLLTEAPLNPK.A	N	63.31	1953.0570	18	0.8	652.0269	3	30.71	20	F20:6520	MAX09859_AndrejP_gel_SA10_BB2_01_19067.mgf	0	0	0	0	0	0	0	98	115			PEAKS DB
K.IWHHTFYNELR.V	N	60.98	1514.7418	11	0.8	505.9194	3	28.89	56	F56:6292	MAX09913_AndrejP_gel_C06_RC6_01_19209.mgf			0	0	0	0	0	87	97			PEAKS DB
K.AGFAGDDAPR.A	N	52.50	975.4410	10	0.6	488.7260	2	13.73	56	F56:2255	MAX09913_AndrejP_gel_C06_RC6_01_19209.mgf			0	0	0	0	0	21	30			PEAKS DB
K.EITALAPSSMK(+14.02).I	N	48.58	1160.6111	11	-2.1	581.3079	2	27.15	66	F66:6035	MAX09918_AndrejP_gel_C11_RD3_01_19219.mgf			0	0	0	0	0	318	328	Methylation(KR)	K11:Methylation(KR):1000.00	PEAKS PTM
K.EITALAPSSM(+15.99)K(+14.02).I	N	46.20	1176.6060	11	0.4	589.3079	2	19.46	55	F55:3689	MAX09913_AndrejP_gel_C06_RC6_01_19208.mgf	0	0	0	0	0	0	0	318	328	Oxidation (M); Methylation(KR)	M10:Oxidation (M):1000.00; K11:Methylation(KR):1000.00	PEAKS PTM
K.IW(+31.99)HHTFYNELR.V	N	45.11	1546.7317	11	0.6	516.5826	3	28.63	55	F55:6246	MAX09913_AndrejP_gel_C06_RC6_01_19208.mgf			0	0	0	0	0	87	97		W2:Dihydroxy:52.86	PEAKS PTM
R.AVFPSIVGRPR.H	N	42.33	1197.6981	11	0.4	599.8540	2	32.95	55	F55:7328	MAX09913_AndrejP_gel_C06_RC6_01_19208.mgf			0	0	0	0	0	31	41			PEAKS DB
R.DLTDYLM(+15.99)K.I	N	39.82	1013.4739	8	1.0	507.7426	2	33.90	56	F56:7492	MAX09913_AndrejP_gel_C06_RC6_01_19209.mgf			0	0	0	0	0	186	193	Oxidation (M)	M7:Oxidation (M):1000.00	PEAKS DB
P.RHT(+79.97)GVMVGMGQ(+.98)KDAYVGDEAQSK.R	N	39.34	2544.1138	23	0.0	637.0330	4	13.88	56	F56:2290	MAX09913_AndrejP_gel_C06_RC6_01_19209.mgf			0	0	0	0	0	41	63	Deamidation (NQ)	T3:Phosphorylation (STY):16.22;Q11:Deamidation (NQ):112.07	PEAKS PTM
R.VAPEEHPT(sub V)LLTEAPLNPK.A	N	38.76	1955.0364	18	0.6	652.6837	3	37.57	56	F56:8450	MAX09913_AndrejP_gel_C06_RC6_01_19209.mgf			0	0	0	0	0	98	115			SPIDER
R.DLTDYLMK.I	N	37.91	997.4790	8	0.6	499.7449	2	45.47	56	F56:10521	MAX09913_AndrejP_gel_C06_RC6_01_19209.mgf			0	0	0	0	0	186	193			PEAKS DB
K.EITALAPSSM(+31.99)K(+14.02).I	N	36.66	1192.6010	11	-1.0	597.3046	2	21.02	55	F55:4110	MAX09913_AndrejP_gel_C06_RC6_01_19208.mgf			0	0	0	0	0	318	328	Sulphone; Methylation(KR)	M10:Sulphone:1000.00;K11:Methylation(KR):1000.00	PEAKS PTM
K.EITALAPSSM(+15.99)K(+42.01).I	N	36.08	1204.6010	11	-1.2	603.3044	2	21.23	55	F55:4207	MAX09913_AndrejP_gel_C06_RC6_01_19208.mgf			0	0	0	0	0	318	328	Oxidation (M); Acetylation (K)	M10:Oxidation (M):1000.00; K11:Acetylation (K):1000.00	PEAKS PTM

total 15 peptides

Peptide	Uniq	-10lgP	Mass	Length	ppm	m/z	z	RT	Fraction	Scan	Source File	Area AndrejP_JAtreatment	Area AndrejP_Feeding	Area AndrejP_CNTR	#Feature	#Feature AndrejP_JAtreatment	#Feature AndrejP_Feeding	#Feature AndrejP_CNTR	Start	End	PTM	AScore	Found By
K.IW(+3.99)HHTFYNELR.V	N	35.86	1518.7368	11	0.5	507.2509	3	28.33	56	F56:6298	MAX09913_AndrejP_gel_C06_RC6_01_19209.mgf			0	0	0	0	0	87	97	Tryptophan oxidation to kynurenin	W2:Tryptophan oxidation to kynurenin:1000.00	PEAKS PTM
total 15 peptides																							

QDK54754.1

back to list

| Protein Coverage | Supporting Peptides |

Protein Coverage:

1 MADEEIQPLV CDNGTGMVK**A GFAGDDAPRA VFPSIVGRPR HTGVMVGMGQ KDAYVGDEAQ SK**RGILTLKY PIEHGIVSNW

81 DDMEK**IWHHT FYNELRVAPE EHPVLLTEAP LNPK**ANREKM TQIMFETFNV PAMYVAIQAV LSLYASGRTT GIVLDSGDGV

161 SHTVPIYEGY ALPHAILRLD LAGRDLTDSL MKILTERGYM FTTTAEREIV RDIKEKLAYV ALDYEQELET AKSSSAIEKN

241 YELPDGQVIT IGAERFRCPE VLFQPSMIGM EAAGIHETTY NSIMKCDVDI RKDLYGNIVL SGGSTMFPGI ADRMSK**EITA**

321 **LAPSSMK**IKV VAPPERKYSV WIGGSILASL STFQQMWISK **GEYDESGPAI VHR**KCF

- a Acetylation (K) (+42.01)
- d Deamidation (NQ) (+0.98)
- m Methylation(KR) (+14.02)
- o Oxidation (M) (+15.99)
- p Propionamide (K, X@N-term) (+71.04)
- s Sulphone (+31.99)
- t Tryptophan oxidation to kynurenin (+3.99)
- T V->T

Supporting Peptides:

Peptide	Uniq	-10lgP	Mass	Length	ppm	m/z	z	RT	Fraction	Scan	Source File	Area AndrejP_JAtreatment	Area AndrejP_Feeding	Area AndrejP_CNTR	#Feature	#Feature AndrejP_JAtreatment	#Feature AndrejP_Feeding	#Feature AndrejP_CNTR	Start	End	PTM	AScore	Found By
R.VAPEEHPVLLTEAPLNPK.A	N	63.31	1953.0570	18	0.8	652.0269	3	30.71	20	F20:6520	MAX09859_AndrejP_gel_SA10_BB2_01_19067.mgf	0	0	0	0	0	0	0	97	114			PEAKS DB
K.IWHHTFYNELR.V	N	60.98	1514.7418	11	0.8	505.9194	3	28.89	56	F56:6292	MAX09913_AndrejP_gel_C06_RC6_01_19209.mgf			0	0	0	0	0	86	96			PEAKS DB
K.AGFAGDDAPR.A	N	52.50	975.4410	10	0.6	488.7260	2	13.73	56	F56:2255	MAX09913_AndrejP_gel_C06_RC6_01_19209.mgf			0	0	0	0	0	20	29			PEAKS DB
K.EITALAPSSMK(+14.02).I	N	48.58	1160.6111	11	-2.1	581.3079	2	27.15	66	F66:6035	MAX09918_AndrejP_gel_C11_RD3_01_19219.mgf			0	0	0	0	0	317	327	Methylation(KR)	K11:Methylation(KR):1000.00	PEAKS PTM
K.EITALAPSSM(+15.99)K(+14.02).I	N	46.20	1176.6060	11	0.4	589.3079	2	19.46	55	F55:3689	MAX09913_AndrejP_gel_C06_RC6_01_19208.mgf	0	0	0	0	0	0	0	317	327	Oxidation (M); Methylation(KR)	M10:Oxidation (M):1000.00; K11:Methylation(KR):1000.00	PEAKS PTM
K.IW(+31.99)HHTFYNELR.V	N	45.11	1546.7317	11	0.6	516.5826	3	28.63	55	F55:6246	MAX09913_AndrejP_gel_C06_RC6_01_19208.mgf			0	0	0	0	0	86	96		W2:Dihydroxy:52.86	PEAKS PTM
R.AVFPSIVGRPR.H	N	42.33	1197.6981	11	0.4	599.8540	2	32.95	55	F55:7328	MAX09913_AndrejP_gel_C06_RC6_01_19208.mgf			0	0	0	0	0	30	40			PEAKS DB
K.G(+71.04)EYDESGPAIVHR.K	Y	42.23	1499.7004	13	0.5	500.9055	3	18.28	56	F56:3484	MAX09913_AndrejP_gel_C06_RC6_01_19209.mgf			0	0	0	0	0	361	373	Propionamide (K, X@N-term)	G1:Propionamide (K, X@N-term):1000.00	PEAKS PTM
P.RHT(+79.97)GVMVGMGQ(+.98)KDAYVGDEAQSK.R	N	39.34	2544.1138	23	0.0	637.0330	4	13.88	56	F56:2290	MAX09913_AndrejP_gel_C06_RC6_01_19209.mgf			0	0	0	0	0	40	62	Deamidation (NQ)	T3:Phosphorylation (STY):16.22;Q11:Deamidation (NQ):112.07	PEAKS PTM
R.VAPEEHPT(sub V)LLTEAPLNPK.A	N	38.76	1955.0364	18	0.6	652.6837	3	37.57	56	F56:8450	MAX09913_AndrejP_gel_C06_RC6_01_19209.mgf			0	0	0	0	0	97	114			SPIDER
K.EITALAPSSM(+31.99)K(+14.02).I	N	36.66	1192.6010	11	-1.0	597.3046	2	21.02	55	F55:4110	MAX09913_AndrejP_gel_C06_RC6_01_19208.mgf			0	0	0	0	0	317	327	Sulphone; Methylation(KR)	M10:Sulphone:1000.00;K11:Methylation(KR):1000.00	PEAKS PTM

total 13 peptides

Peptide	Uniq	-10lgP	Mass	Length	ppm	m/z	z	RT	Fraction	Scan	Source File	Area AndrejP_JAtreatment	Area AndrejP_Feeding	Area AndrejP_CNTR	#Feature	#Feature AndrejP_JAtreatment	#Feature AndrejP_Feeding	#Feature AndrejP_CNTR	Start	End	PTM	AScore	Found By
K.EITALAPSSM(+15.99)K(+42.01).I	N	36.08	1204.6010	11	-1.2	603.3044	2	21.23	55	F55:4207	MAX09913_AndrejP_gel_C06_RC6_01_19208.mgf			0	0	0	0	0	317	327	Oxidation (M); Acetylation (K)	M10:Oxidation (M):1000.00; K11:Acetylation (K):1000.00	PEAKS PTM
K.IW(+3.99)HHTFYNELR.V	N	35.86	1518.7368	11	0.5	507.2509	3	28.33	56	F56:6298	MAX09913_AndrejP_gel_C06_RC6_01_19209.mgf			0	0	0	0	0	86	96	Tryptophan oxidation to kynurenin	W2:Tryptophan oxidation to kynurenin:1000.00	PEAKS PTM
**total 13 peptides**																							

BAW35430.1

back to list

| Protein Coverage | Supporting Peptides |

Protein Coverage:

```
  1 MIQYYSMMDW RNPSLQVLII TILVLSLTSS SSYAHRVYPH GHEYKIEDYD HTMISDFRLI NRMSLIKCPI ESAYIQINTS
 81 ATSTLPNEGN ITVTVSGVLH PATGDWVAMV SPSTADIGSC PLDAVMYMQT GDLASLPLLC HYPVKAQYVS NDPGYLSCQN
161 SACQDSSCLI KTCSGSITFH YTNIRTDLIF VFFAGGVKTP CILATTQPIQ FANPASPLYG HLSSIDSTAT SMTLTWVSGD
241 QAPQQVLYGN NQSQTSVVTT FTQNDMCTTT LLPSPAKDFG WHDPGYIHSA VMTGLLPSTT YSYQYGSNSV GWSNQIQFRT
321 PPAGGSNELT FLAFADMGKT PLDPSIEHYI QPGAVSVIQA MASEVAAGNI DAIYHIGDIS YATGFLVEWD FFLNLIKYIA
401 SSVSYMTSIG NHERDYISSG SVYITPDSGG ECGVPYETYF PMPTPAKDKP WYSIEQGPVH FTVISTEHPW SVNSEQYNWM
481 QSDMASVNRS RTPWLIFAGH RPMYSSIKGL EILNVDPRFV SAVEPLLLQY QVDLALFGHV HNYERLCAVY QKQCLGMPVK
561 DANGIDTYNN GNYTAPVHVI IGMAGFTLDS FTSNPNSWSL VRISEFGYSK VQATSNSLLF QFLNAHTRQI DDSFYITK
```

C Carbamidomethylation (+57.02)
f Formylation (+27.99)
m Methylation(KR) (+14.02)
m Methylation(others) (+14.02)

Supporting Peptides:

Peptide	Uniq	-10lgP	Mass	Length	ppm	m/z	z	RT	Fraction	Scan	Source File	Area AndrejP_JAtreatment	Area AndrejP_Feeding	Area AndrejP_CNTR	#Feature	#Feature AndrejP_JAtreatment	#Feature AndrejP_Feeding	#Feature AndrejP_CNTR	Start	End	PTM	AScore	Found By
Q.VDLALFGHVHNYER.L	N	68.52	1668.8372	14	0.3	557.2850	3	34.41	13	F13:7479	MAX09856_AndrejP_gel_SA07_BA7_01_19060.mgf	0	0		0	0	0	0	532	545			PEAKS DB
Q.VDLALFGHVHNYER(+14.02).L	N	51.23	1682.8529	14	3.0	561.9615	3	36.21	21	F21:8314	MAX09860_AndrejP_gel_SA11_BB3_01_19068.mgf	0			0	0	0	0	532	545	Methylation(KR)	R14:Methylation(KR):1000.00	PEAKS PTM
Q.VD(+14.02)LALFGHVHNYER.L	N	48.89	1682.8529	14	-1.5	561.9548	3	34.21	17	F17:7586	MAX09858_AndrejP_gel_SA09_BB1_01_19064.mgf	0	0	0	0	0	0	0	532	545	Methylation(others)	D2:Methylation(others):156.69	PEAKS PTM
K.GLEILNVDPR(+14.02).F	Y	46.16	1138.6346	10	-1.3	570.3218	2	48.43	41	F41:12069	MAX09906_AndrejP_gel_F10_RB2_01_19191.mgf	0	0		0	0	0	0	509	518	Methylation(KR)	R10:Methylation(KR):1000.00	PEAKS PTM
K.GLE(+14.02)ILNVDPR.F	Y	45.98	1138.6346	10	-1.6	570.3220	2	46.32	42	F42:11432	MAX09906_AndrejP_gel_F10_RB2_01_19192.mgf	0	0		0	0	0	0	509	518	Methylation(others)	E3:Methylation(others):142.99	PEAKS PTM
Q.YQVD(+14.02)LALFGHVHNYER.L	Y	45.33	1973.9747	16	16.9	494.5067	4	43.24	40	F40:10305	MAX09905_AndrejP_gel_F09_RB1_01_19190.mgf		0		0	0	0	0	530	545	Methylation(others)	D4:Methylation(others):181.88	PEAKS PTM
K.GLEILNVDPR.F	Y	45.14	1124.6189	10	-1.1	563.3141	2	43.91	41	F41:10810	MAX09906_AndrejP_gel_F10_RB2_01_19191.mgf	0	0		0	0	0	0	509	518			PEAKS DB
S.LPLLC(+57.02)HYPVK.A	N	40.49	1238.6846	10	1.7	620.3524	2	25.97	21	F21:5625	MAX09860_AndrejP_gel_SA11_BB3_01_19068.mgf	0	0		0	0	0	0	136	145	Carbamidomethylation	C5:Carbamidomethylation:1000.00	PEAKS DB
Q.V(+27.99)DLALFGHVHNYER.L	N	40.00	1696.8322	14	25.5	425.2274	4	35.17	21	F21:8000	MAX09860_AndrejP_gel_SA11_BB3_01_19068.mgf	0			0	0	0	0	532	545	Formylation	V1:Formylation:1000.00	PEAKS PTM
K.DKPWYSIEQGPVHF.T	N	35.62	1701.8151	14	0.1	568.2773	3	54.87	42	F42:13835	MAX09906_AndrejP_gel_F10_RB2_01_19192.mgf		0		0	0	0	0	448	461			PEAKS DB
**total 10 peptides**																							

KAH9619639.1

back to list

| Protein Coverage | Supporting Peptides |

Protein Coverage:

1 MLNRRKLIQC PLNVNPYLKI TTNVGSSSLL DDQNVIVTVS GVLQPSDKHW VAMISPSHSS VQSCLLNTLL YKETGDLSHL

81 PLLCHYPVKR AGLQRVGPGW KRAGLWEREP EPTLRARGVR ARPGRAGDLV ALDRPDWEKM GAGQCGLGGL GVWAGENGAR

161 DRPGLGKRVG RAGVAGQRAG EVGARLGGPD IEFVLFANGF DTPCILKRTD QPLPFVTPNK PLYPHLSAVD STATAMRVTW

241 VSGSIEPQQV EYGNAKSKIS SVTTFTQADM CGNAVKSPAQ DFGWHNPGYI HTAVMTGLTP STISSYKLGS DSVGWSDNIQ

321 FRTPPVGGSA ELRFLAFGDM GKAPQDGTVE HYIQPGSLSV AAALTEEVAS GKVDSIFHIG DISYATGFLV EWDYFLHQIT

401 PFASRLAYMT AIGNHERDYI DSGSVYITPD SGGECGVPYE TYFQMPTPAK DKPWYSIEQG PVHFTVISTE HDWKPNSEQY

481 KWMKSDMASV NRARTPWLIF TGHRPMYTSA KAALISIDPL FVNYVEPLLL QNKVDLALFG HVHNYERTCA IYKAQCKAMP

561 IKDENGIDTY NNLNYTAPVQ AVIGMAGFSL DEFSDNVHDW SLSRISKFGY TRIQATNKQL KFEVSVAIL

a Acetylation (K) (+42.01)
c Carbamidomethylation (+57.02)
f Formylation (+27.99)
g Guanidination (+42.02)
m Methylation(KR) (+14.02)
m Methylation(others) (+14.02)
o Oxidation (M) (+15.99)

Supporting Peptides:

Peptide	Uniq	-10lgP	Mass	Length	ppm	m/z	z	RT	Fraction	Scan	Source File	Area AndrejP_JAtreatment	Area AndrejP_Feeding	Area AndrejP_CNTR	#Feature	#Feature AndrejP_JAtreatment	#Feature AndrejP_Feeding	#Feature AndrejP_CNTR	Start	End	PTM	AScore	Found By
K.VDLALFGHVHNYER.T	N	68.52	1668.8372	14	0.3	557.2850	3	34.41	13	F13:7479	MAX09856_AndrejP_gel_SA07_BA7_01_19060.mgf	0	0		0	0	0	0	534	547			PEAKS DB
K.VDLALFGHVHNYER(+14.02).T	N	51.23	1682.8529	14	3.0	561.9615	3	36.21	21	F21:8314	MAX09860_AndrejP_gel_SA11_BB3_01_19068.mgf	0			0	0	0	0	534	547	Methylation(KR)	R14:Methylation(KR):1000.00	PEAKS PTM
K.VD(+14.02)LALFGHVHNYER.T	N	48.89	1682.8529	14	-1.5	561.9548	3	34.21	17	F17:7586	MAX09858_AndrejP_gel_SA09_BB1_01_19064.mgf	0	0	0	0	0	0	0	534	547	Methylation(others)	D2:Methylation(others):156.69	PEAKS PTM
R.TPWLIFTGHRPM(+15.99)YTSAK(+42.02).A	Y	44.39	2063.0410	17	11.5	516.7716	4	51.42	41	F41:12943	MAX09906_AndrejP_gel_F10_RB2_01_19191.mgf		0	0	0	0	0	0	495	511	Oxidation (M); Guanidination	M12:Oxidation (M):1000.00;K17:Guanidination:1000.00	PEAKS PTM
R.TPWLIFTGHRPM(+15.99)YTSAK(+42.01).A	Y	43.02	2063.0298	17	16.7	516.7706	4	51.78	40	F40:12744	MAX09905_AndrejP_gel_F09_RB1_01_19190.mgf		0	0	0	0	0	0	495	511	Oxidation (M); Acetylation (K)	M12:Oxidation (M):1000.00;K17:Acetylation (K):1000.00	PEAKS PTM
H.LPLLC(+57.02)HYPVK.R	N	40.49	1238.6846	10	1.7	620.3524	2	25.97	21	F21:5625	MAX09860_AndrejP_gel_SA11_BB3_01_19068.mgf	0	0		0	0	0	0	80	89	Carbamidomethylation	C5:Carbamidomethylation:1000.00	PEAKS DB
K.V(+27.99)DLALFGHVHNYER.T	N	40.00	1696.8322	14	25.5	425.2274	4	35.17	21	F21:8000	MAX09860_AndrejP_gel_SA11_BB3_01_19068.mgf	0			0	0	0	0	534	547	Formylation	V1:Formylation:1000.00	PEAKS PTM
K.DKPWYSIEQGPVHF.T	N	35.62	1701.8151	14	0.1	568.2773	3	54.87	42	F42:13835	MAX09906_AndrejP_gel_F10_RB2_01_19192.mgf		0		0	0	0	0	451	464			PEAKS DB
**total 8 peptides**																							

MCK0600380.1

back to list

| Protein Coverage | Supporting Peptides |

Protein Coverage:

1 QKYKVDIALF GHVHNYERTC PIYENICTRK GRDYYQGPLN GTIHVVAGGA GASLTEFANF QPRWSLYRDF DYGFVKLTAF

81 DHSNLLFEYK KSRDGKVYDS FRISRDYRDI LACFADSCPP GTLAS

m Methylation(KR) (+14.02)
m Methylation(others) (+14.02)

Supporting Peptides:

Peptide	Uniq	-10lgP	Mass	Length	ppm	m/z	z	RT	Fraction	Scan	Source File	Area AndrejP_JAtreatment	Area AndrejP_Feeding	Area AndrejP_CNTR	#Feature	#Feature AndrejP_JAtreatment	#Feature AndrejP_Feeding	#Feature AndrejP_CNTR	Start	End	PTM	AScore	Found By
K.VDIALFGHVHNYER.T	N	68.52	1668.8372	14	0.3	557.2850	3	34.41	13	F13:7479	MAX09856_AndrejP_gel_SA07_BA7_01_19060.mgf	0	0		0	0	0	0	5	18			PEAKS DB
K.YK(+14.02)VDIALFGHVHNYER(+14.02).T	Y	61.22	1988.0267	16	-0.6	498.0119	4	44.88	41	F41:11108	MAX09906_AndrejP_gel_F10_RB2_01_19191.mgf		0	0	0	0	0	0	3	18	Methylation(KR)	K2:Methylation(KR):1000.00;R16:Methylation(KR):1000.00	PEAKS PTM

total 7 peptides

Peptide	Uniq	-10lgP	Mass	Length	ppm	m/z	z	RT	Fraction	Scan	Source File	Area AndrejP_JAtreatment	Area AndrejP_Feeding	Area AndrejP_CNTR	#Feature	#Feature AndrejP_JAtreatment	#Feature AndrejP_Feeding	#Feature AndrejP_CNTR	Start	End	PTM	AScore	Found By
K.VDIALFGHVHNYER(+14.02).T	N	51.41	1682.8529	14	3.0	561.9615	3	36.21	21	F21:8314	MAX09860_AndrejP_gel_SA11_BB3_01_19068.mgf	0			0	0	0	0	5	18	Methylation(KR)	R14:Methylation(KR):1000.00	PEAKS PTM
K.YK(+14.02)VDIALFGHVHNYER.T	Y	51.17	1974.0111	16	0.5	494.5088	4	42.74	42	F42:10351	MAX09906_AndrejP_gel_F10_RB2_01_19192.mgf	0	0	0	0	0	0	0	3	18	Methylation(KR)	K2:Methylation(KR):131.98	PEAKS PTM
K.YKVD(+14.02)IALFGHVHNYER.T	Y	51.10	1974.0111	16	-1.8	494.5076	4	42.77	62	F62:10403	MAX09916_AndrejP_gel_C09_RD1_01_19215.mgf			0	0	0	0	0	3	18	Methylation(others)	D4:Methylation(others):151.86	PEAKS PTM
K.VD(+14.02)IALFGHVHNYER.T	N	49.05	1682.8529	14	-1.5	561.9548	3	34.21	17	F17:7586	MAX09858_AndrejP_gel_SA09_BB1_01_19064.mgf	0	0	0	0	0	0	0	5	18	Methylation(others)	D2:Methylation(others):156.69	PEAKS PTM
K.VD(+28.03)IALFGHVHNYER.T	N	34.93	1696.8685	14	-2.0	566.6260	3	43.20	40	F40:10260	MAX09905_AndrejP_gel_F09_RB1_01_19190.mgf		0		0	0	0	0	5	18		D2:Ethylation:12.28	PEAKS PTM
total 7 peptides																							

XP_010695592.2

back to list

| Protein Coverage | Supporting Peptides |

Protein Coverage:

```
  1 MSGRGKGGKG LGKGGAKRHR KVLRDNIQGI TKPAIRRLAR RGGVKRISGL IYEETRGVLK IFLENVIRDA VTYTEHARRK
 81 TVTAMDVVYA LKRQGRTLYG FGGKPNLKIS TIYPLYNHPP LLSSFPQQSH IHNTTQNNSN TKNHRKKKTQ KRREKMSGRG
161 KGGKGLGKGG AKRHRKVLRD NIQGITKPAI RRLARRGGVK RISGLIYEET RGVLKIFLEN VIRDAVTYTE HARRKTVTAM
241 DVVYALKRQG RTLYGFGG
```

Oxidation (M) (+15.99)

Supporting Peptides:

Peptide	Uniq	-10lgP	Mass	Length	ppm	m/z	z	RT	Fraction	Scan	Source File	Area AndrejP_JAtreatment	Area AndrejP_Feeding	Area AndrejP_CNTR	#Feature	#Feature AndrejP_JAtreatment	#Feature AndrejP_Feeding	#Feature AndrejP_CNTR	Start	End	PTM	AScore	Found By
K.TVTAMDVVYALK.R	Y	68.22	1309.6952	12	-3.2	655.8486	2	56.19	66	F66:13686	MAX09918_AndrejP_gel_C11_RD3_01_19219.mgf			0	0	0	0	0	81	92			PEAKS DB
K.TVTAM(+15.99)DVVYALK.R	Y	51.54	1325.6901	12	-2.1	663.8464	2	46.16	65	F65:10928	MAX09918_AndrejP_gel_C11_RD3_01_19218.mgf			0	0	0	0	0	81	92	Oxidation (M)	M5:Oxidation (M):1000.00	PEAKS DB
R.ISGLIYEETR.G	Y	41.95	1179.6135	10	-1.8	590.8092	2	30.21	66	F66:6816	MAX09918_AndrejP_gel_C11_RD3_01_19219.mgf	0	0	0	0	0	0	0	47	56			PEAKS DB
total 3 peptides																							

KMT05278.1

back to list

| Protein Coverage | Supporting Peptides |

Protein Coverage:

```
  1 MSGKGEGPAI GIDLGTTYSC VGVWQHDRVE IIANDQGNRT TPSYVAFTDT ERLIGDAAKN QVAMNPTNTV FDAKRLIGRR
 81 FSDPSVQSDM KLWPFKVIPG PADKPMIVVN HKGEEKQFSA EEVSSMVLTK MKEIAEAYLG TTIKNAVVTV PAYFNDSQRQ
161 ATKDAGVISG LNVMRIINEP TAAAIAYGLD KKATSSGEKN VLIFDLGGGT FDVSLLTIEE GIFEVKATAG DTHLGGEDFD
241 NRMVNHFVQE FKRKHKKDIN GNPRALRRLR TACERAKRTL SSTAQTTIEI DSLYEGVDFY TTITRARFEE LNMDLFRKCM
321 EPVEKCLRDA KMDKNSVHDV VLVGGSTRIP KVQQLLQDFF NGKELCKSIN PDEAVAYGAA VQAAILSGEG NEKVQDLLLL
401 DVTPLSLGLE TAGGVMTVLI PRNTTIPTKK EQVFSTYSDN QPGVLIQVFE GERTRTRDNN LLGKFELTGI PPAPRGVPQI
481 NVCFDIDANG ILNVSAEDKT TGQKNKITIT NDKGRLSKEE IERMVQEAEK YKSEDEEHKK KVESKNALEN YAYNMRNTIK
561 DEKISSKLSE ADKKKIEDAI EQAVQWLDGN QLAEADEFDD KMKELEGICN PIIAKMYQGS ADMGGQMDED GPAPGGASGA
641 GPKIEEVD
```

Methylation(KR) (+14.02)

Supporting Peptides:

Peptide	Uniq	-10lgP	Mass	Length	ppm	m/z	z	RT	Fraction	Scan	Source File	Area AndrejP_JAtreatment	Area AndrejP_Feeding	Area AndrejP_CNTR	#Feature	#Feature AndrejP_JAtreatment	#Feature AndrejP_Feeding	#Feature AndrejP_CNTR	Start	End	PTM	AScore	Found By
K.FELTGIPPAPR(+14.02).G	Y	54.39	1210.6709	11	-1.5	606.3380	2	43.88	66	F66:10271	MAX09918_AndrejP_gel_C11_RD3_01_19219.mgf			0	0	0	0	0	465	475	Methylation(KR)	R11:Methylation(KR):1000.00	PEAKS PTM
R.VEIIANDQGNR.T	N	49.09	1227.6207	11	-5.2	614.8103	2	15.77	65	F65:2829	MAX09918_AndrejP_gel_C11_RD3_01_19218.mgf			0	0	0	0	0	29	39			PEAKS DB
R.TTPSYVAFTDTER.L	N	44.02	1486.6940	13	-2.0	744.3478	2	32.49	65	F65:7434	MAX09918_AndrejP_gel_C11_RD3_01_19218.mgf			0	0	0	0	0	40	52			PEAKS DB
total 3 peptides																							

MCK0637628.1

back to list

| Protein Coverage | Supporting Peptides |

Protein Coverage:

1 MEKFRSLSTL LLIAALLFNC TSALQKLGET CASNRNCGSG LHCETCLANS NLRPRCTRIQ PLNPYSKVKG LPFNRYSWLT
81 THNSFARLGQ KSATGSVVIA PTNQQDSITD QLNNGVRGLM LDMYDFQNDV WLCHSFGGKC YNYTAFQPAI NVLNEIRIFL
161 EANPSEIVSI FIEDYVTSPK GLTKVFDAAG LRKFWFPVSR MPKNGGDWPI IDDMIQKNQR LLVFTSKSSK EASEGIAYEW
241 RYLVENQYGN GGMVAGSCPN RAESPTMNTT SRSLVLVNYF PDVPDLATAC KHNSAPLISM LNTCYNASAK R**WANFIAVDF**
321 **YKR**SDGGGAP EAVDEASGHL VCGCGNIADC RANMTYGSCD TPEAVPNAPE TAPASSFAYS TQLQFWWILG ISLTSLISL

312

Tryptophan oxidation to kynurenin (+3.99)

Supporting Peptides:

Peptide	Uniq	-10lgP	Mass	Length	ppm	m/z	z	RT	Fraction	Scan	Source File	Area AndrejP_JAtreatment	Area AndrejP_Feeding	Area AndrejP_CNTR	#Feature	#Feature AndrejP_JAtreatment	#Feature AndrejP_Feeding	#Feature AndrejP_CNTR	Start	End	PTM	AScore	Found By
R.W(+31.99)ANFIAVDFYK.R	Y	66.34	1404.6714	11	-2.1	703.3375	2	57.48	39	F39:14332	MAX09905_AndrejP_gel_F09_RB1_01_19189.mgf		0		0	0	0	0	312	322		W1:Dihydroxy:47.09	PEAKS PTM
R.WANFIAVDFYK.R	Y	57.01	1372.6815	11	-1.9	687.3431	2	57.80	40	F40:14533	MAX09905_AndrejP_gel_F09_RB1_01_19190.mgf	0	0	0	0	0	0	0	312	322			PEAKS DB
R.W(+3.99)ANFIAVDFYK.R	Y	55.22	1376.6764	11	-0.8	689.3413	2	57.73	40	F40:14500	MAX09905_AndrejP_gel_F09_RB1_01_19190.mgf		0	0	0	0	0	0	312	322	Tryptophan oxidation to kynurenin	W1:Tryptophan oxidation to kynurenin:1000.00	PEAKS PTM
R.WANFIAVDFYKR.S	Y	51.17	1528.7826	12	-1.0	510.5992	3	56.78	35	F35:14199	MAX09903_AndrejP_gel_F07_RA7_01_19185.mgf		0		0	0	0	0	312	323			PEAKS DB
**total 4 peptides**																							

ASZ85176.1

back to list

| Protein Coverage | Supporting Peptides |

Protein Coverage:

1 MGKDDKTHIN VVVIGHVDSG KSTTTGHLIY QCGGIDKRTI EKFEKEAAEM GKGSFKYAWV LDKLKAERER GITIDIALWK
81 FETPKYYVTV IDAPGHRDFI KNMITGTSQA DCAILIIAAG TGEFEAGISK DGQTR**EHALL AYTLGVK**QLI VAINKMDTTK
161 WSEARFQEII KETSNFIKKV GYNPKTVAFV PISGFHGDNM LSPSTNCPWY KGWEKETKAG KSTGKTLLEA IDSIEPPKRP
241 SDKPLR**LPLQ DVYKIGGIGT VPVGR**IETGV IKPGMVVTFA PSNVTTEVKS VEMHHEQLTE GVPGDNVGFN VKNVSVKEIR
321 RGNVAGDSKN DPPLGAASFD AQVIVLNHPG QVGAGYAPVL DCHTAHIACK FAEIKEKIDR RTGKSVESAP KFIKSGDSAI
401 VKMIPSKPMC VEAFTDYPPL GRFAVRDMRQ TVAVGVIKSV EKAAAGSGKV TKSAAKATKK

Supporting Peptides:

Peptide	Uniq	-10lgP	Mass	Length	ppm	m/z	z	RT	Fraction	Scan	Source File	Area AndrejP_JAtreatment	Area AndrejP_Feeding	Area AndrejP_CNTR	#Feature	#Feature AndrejP_JAtreatment	#Feature AndrejP_Feeding	#Feature AndrejP_CNTR	Start	End	PTM	AScore	Found By
K.IGGIGTVPVGR.I	N	45.70	1024.6029	11	-0.4	513.3059	2	18.54	3	F3:3437	MAX09851_AndrejP_gel_SA02_BA2_01_19050.mgf	0	0	0	0	0	0	0	255	265			PEAKS DB
R.LPLQDVYK.I	N	43.07	974.5436	8	-1.5	488.2784	2	22.02	20	F20:4369	MAX09859_AndrejP_gel_SA10_BB2_01_19067.mgf	0			0	0	0	0	247	254			PEAKS DB
R.EHALLAYTLGVK.Q	Y	41.82	1313.7343	12	-5.9	657.8661	2	42.64	65	F65:9907	MAX09918_AndrejP_gel_C11_RD3_01_19218.mgf			0	0	0	0	0	136	147			PEAKS DB
**total 3 peptides**																							

XP_021841864.1

back to list

| Protein Coverage | Supporting Peptides |

Protein Coverage:

1 MSAFVGKYAD ELIKTAKYIA TPGK**GILAAD ESTGTIGK**RF ASINVENIES NRQTLRELLF TSPGVLTLLS GVILFEETLY
81 QKTSEGKLFA ELLQENGVVP GIKVDKGVVD LAGTNGETTT QGFDNLGARC QEYYKAGARF AKWRAVLKIG PTEPSELSIQ
161 QNAQGLARYA IICQENGLVP IVEPEILTDG NHDIKKCAAV TETVLAAVYK ALNDQHVLLE GTLLKPNMVT PGSDSPKVAA
241 EVIAEHTVTA LRRTVPPAVP GIVFLSGGQS EEEATLNLNA MNKLDVLKPW TLSFSFGRAL QKSTLQIWGG KKENVSKAQE
321 VFLARCKANS EATLGKYVAG GSGADGAASE SLYEKGYKY

Supporting Peptides:

Peptide	Uniq	-10lgP	Mass	Length	ppm	m/z	z	RT	Fraction	Scan	Source File	Area AndrejP_JAtreatment	Area AndrejP_Feeding	Area AndrejP_CNTR	#Feature	#Feature AndrejP_JAtreatment	#Feature AndrejP_Feeding	#Feature AndrejP_CNTR	Start	End	PTM	AScore	Found By
K.GILAADESTGTIGK.R	Y	67.48	1331.6932	14	0.2	666.8511	2	24.54	56	F56:5197	MAX09913_AndrejP_gel_C06_RC6_01_19209.mgf			0	0	0	0	0	25	38			PEAKS DB
K.GILAADESTGTIGK(+27.99).R	Y	49.21	1359.6881	14	0.4	680.8486	2	27.68	55	F55:6028	MAX09913_AndrejP_gel_C06_RC6_01_19208.mgf			0	0	0	0	0	25	38		K14:Formylation: 81.10	PEAKS PTM
**total 2 peptides**																							

KAH9613912.1

back to list

| Protein Coverage | Supporting Peptides |

Protein Coverage:

1 MGKEKIHISI VVIGHVDSGK **STTTGHLIYK** LGGIDKRVIE RFEKEAAEMN KRSFKYAWVL DKLKAERERG ITIDIALWKF
81 ETTKYYCTVI DAPGHRDFIK NMITGTSQAD CAVLIIDSTT GGFEAGISKD GQTREHALLA FTLGVKQMIC CLNKMDATTP
161 KYSKSRYEEI VKEVGSYLKK VGYNPEKIPF VPISGFEGDN MIDRSTNLEW YKGPTLLEAL DQVSEPKRPS DKPLR**LPLQD**
241 **VYKIGGIGTV PVGR**VETGVI KPGMLVTFGP SGLTTEVKSV EMHHESMPEA LPGDNVGFNV KNVAVKDLKR GFVASDSKND
321 PAKEAANFTS QVIIMNHPGQ IGNGYAPVLD CHTSHIAVKF AELVTKIDRR SGKELEKEPK FLKNGDAGMV KMIPTKPMVV
401 ETFSEYPPLG RFAVRDMRQT VAVGVIKNVE KKDPTGAKVT KAALKKK

Supporting Peptides:

Peptide	Uniq	-10lgP	Mass	Length	ppm	m/z	z	RT	Fraction	Scan	Source File	Area AndrejP_JAtreatment	Area AndrejP_Feeding	Area AndrejP_CNTR	#Feature	#Feature AndrejP_JAtreatment	#Feature AndrejP_Feeding	#Feature AndrejP_CNTR	Start	End	PTM	AScore	Found By
K.STTTGHLIYK.L	Y	47.50	1119.5924	10	-2.5	560.7985	2	15.17	66	F66:2681	MAX09918_AndrejP_gel_C11_RD3_01_19219.mgf			0	0	0	0	0	21	30			PEAKS DB
K.IGGIGTVPVGR.V	N	45.70	1024.6029	11	-0.4	513.3059	2	18.54	3	F3:3437	MAX09851_AndrejP_gel_SA02_BA2_01_19050.mgf	0	0	0	0	0	0	0	244	254			PEAKS DB
R.LPLQDVYK.I	N	43.07	974.5436	8	-1.5	488.2784	2	22.02	20	F20:4369	MAX09859_AndrejP_gel_SA10_BB2_01_19067.mgf	0			0	0	0	0	236	243			PEAKS DB
total 3 peptides																							

XP_021760870.1

back to list

| Protein Coverage | Supporting Peptides |

Protein Coverage:

1 MAKEKMPAIG IDLGTTNSCV AVWRNGKVEI ITNDQGNRTT PSYVAFTKKE RLIGDAAKNQ ATINSANTIF DVKRLIGRGF
81 YDEAVQNDIK LWPFKVVPSN DEDKKPLIVV TYMGKEKKFT AEEISAMVLS KIKEIAEAYL GVTVKNVVVT VPAYFNNLQR
161 QATKDAGVIA GLNIIR**IINE PTAAAIAYGL DRK**AATGKTN VMVFDLGGGT LDVSLITIES DAFEVKSVSG DTHLGGEDFD
241 NRIVNYFVNE IKRKHRKDIS GNSKAIGRLR AACEKAKRIL SSAAVTSIDI DCLFEGIDFS SSFPRARFEK LSFDLCKRCI
321 GPVEKCLKDA KMEKKDVDEI VLVGGSSRIP KIQELLSKFF DGKQLCKSIN PDEAVAHGAA AHAAILSCVD NEDYVLVDVT
401 PLSLGIENHR GEMSVIIPRN TPIPVMKVKE FTTTEDNQSE ITFNVYEGEN PIANKNNFLG VFELSGIPPA PKENPNIKAW
481 FEIDADSIMK CYAQDEASGS KNGITISYQT GNLSKEDVER MLREAEKYRY EDELCKVKVQ AMNELEEYLE DMRYFLEAEK
561 SIVLEDRNMM EDVIEKTVQW MDWNFLLTEI SKFKDKLKEL KSVCEPIVKK MDKLCDAEAG ITNVGNDIVE VD

Supporting Peptides:

Peptide	Uniq	-10lgP	Mass	Length	ppm	m/z	z	RT	Fraction	Scan	Source File	Area AndrejP_JAtreatment	Area AndrejP_Feeding	Area AndrejP_CNTR	#Feature	#Feature AndrejP_JAtreatment	#Feature AndrejP_Feeding	#Feature AndrejP_CNTR	Start	End	PTM	AScore	Found By
R.IINEPTAAAIAYGLDR.K	Y	64.35	1686.8940	16	-2.0	844.4473	2	52.57	66	F66:12650	MAX09918_AndrejP_gel_C11_RD3_01_19219.mgf			0	0	0	0	0	177	192			PEAKS DB
R.IINEPTAAAIAYGLDRK.A	Y	37.99	1814.9890	17	-1.8	605.9987	3	47.08	66	F66:11172	MAX09918_AndrejP_gel_C11_RD3_01_19219.mgf			0	0	0	0	0	177	193			PEAKS DB
total 2 peptides																							

KAH9602483.1

back to list

| Protein Coverage | Supporting Peptides |

Protein Coverage:

1 MAGKGEGPAI GIDLGTTYSC VGVWQHDR**VE IIANDQGNRT TPSYVAFTDT ER**LIGDAAKN QVAMNPINTV FDAKRLIGRR
81 FSDASVQDDM KHWPFKVTPG PGDKPMIGVN YKGEDKQFSA EEISSMVLIK MKEIAEAFLG STVKNAVVTV PAYFNDSQRQ
161 ATK**DAGVIAG INVLR**IINEP TAAAIAYGLD KKATSTGEKN VLIFDLGGGT FDVSLLTIEE GIFEVKATAG DTHLGGEDFD
241 NRMVNHFVQE FKRKNKKDIS GNPRALRRLR TACERAKRTL SSTAQTTIEI DSLYEGVDFY TTITRARFEE LNMDLFRKCM
321 EPVEKCLRDA KMDKSTVHDV VLVGGSTRIP KVQQLLQDFF NGKELCKSIN PDEAVAYGAA VQAAILSGEG NEKVQDLLLL
401 DVTPLSLGLE TAGGVMTVLI PRNTTIPTKK EQVFSTYSDN QPGVLIQVYE GERTRTRDNN LLGKFELSGI PPAPRGVPQI
481 TVCFDIDANG ILNVSAEDKT TGQKNKITIT NDKGRLSKDE IEKMVQEAEK YKSEDEEHKK KVESKNALEN YAYNMRNTVR
561 DEKIAGKLAE DDKKKIDDAI DQTISWLDSN QLAEADEFDD KMKELESICN PIIAKMYQGA GGEAGAPMDD DIPAGSGGAG
641 PKIEEVD

Supporting Peptides:

Peptide	Uniq	-10lgP	Mass	Length	ppm	m/z	z	RT	Fraction	Scan	Source File	Area AndrejP_JAtreatment	Area AndrejP_Feeding	Area AndrejP_CNTR	#Feature	#Feature AndrejP_JAtreatment	#Feature AndrejP_Feeding	#Feature AndrejP_CNTR	Start	End	PTM	AScore	Found By
R.VEIIANDQGNR.T	N	49.09	1227.6207	11	-5.2	614.8103	2	15.77	65	F65:2829	MAX09918_AndrejP_gel_C11_RD3_01_19218.mgf			0	0	0	0	0	29	39			PEAKS DB
R.TTPSYVAFTDTER.L	N	44.02	1486.6940	13	-2.0	744.3478	2	32.49	65	F65:7434	MAX09918_AndrejP_gel_C11_RD3_01_19218.mgf			0	0	0	0	0	40	52			PEAKS DB
K.DAGVIAGINVLR.I	Y	36.57	1196.6877	12	-2.1	599.3461	2	52.88	66	F66:12743	MAX09918_AndrejP_gel_C11_RD3_01_19219.mgf			0	0	0	0	0	164	175			PEAKS DB
total 3 peptides																							

KAH9624815.1

back to list

| Protein Coverage | Supporting Peptides |

Protein Coverage:

```
  1 MAKLLFLCIF FIIFFFVTPL SAILSPFNDL RPRNLIESLN LFPKKSVNII EDNNGDLPKL DERLVEKWFK FPNFYDASGI
 81 SVEDLGHHAG YYQIEHSHDA RMFYFFFESR RSKEDPVVIW LTGGPGCSSE LALFYENGPF KIANNLSLVW NEYGWDQVSN
161 LIYVDQPIGT GFSYSSNRRD IRHDEEGVSS DLYDFLQAFF TKHPELVNND FYITGESYAG HYIPAFAARL HQGKKAEEDI
241 HINLKGFAIG NGLTDPAVQY AAYPDYALEM GLIKKSSYNL IKKMVPLCEL SVKLCGTDGT LSCTASFFLC NTIFGSIKLL
321 IGDANYYDVR KKCVGKLCYD FSSMEKFLNQ ASVRTALGVG DIDFVSCSTT VYAAMLVDWM RNLEVGIPAL LQDDIKLLVY
401 AGEYDLICNW LGNSRWVHAM KWSGQTDFVA SPEVPFEVDG SQAGILKSHG PLSFLKVHDA GHMVPMDQPK AALEMLKRWT
481 EGSLSKATSI PGKSVASY
```

d Deamidation (NQ) (+0.98)
m Methylation(KR) (+14.02)
o Oxidation (M) (+15.99)

102 110 392 396 475 477

Supporting Peptides:

Peptide	Uniq	-10lgP	Mass	Length	ppm	m/z	z	RT	Fraction	Scan	Source File	Area AndrejP_JAtreatment	Area AndrejP_Feeding	Area AndrejP_CNTR	#Feature	#Feature AndrejP_JAtreatment	#Feature AndrejP_Feeding	#Feature AndrejP_CNTR	Start	End	PTM	AScore	Found By
R.M(+15.99)FYFFFESR.R	Y	49.72	1288.5586	9	-2.9	645.2810	2	57.86	39	F39:14463	MAX09905_AndrejP_gel_F09_RB1_01_19189.mgf	0	0	0	0	0	0	0	102	110	Oxidation (M)	M1:Oxidation (M):1000.00	PEAKS DB
R.MFYFFFESR.R	Y	46.64	1272.5637	9	-4.1	637.2829	2	58.43	39	F39:14657	MAX09905_AndrejP_gel_F09_RB1_01_19189.mgf		0		0	0	0	0	102	110			PEAKS DB
R.M(+15.99)FYFFFESR(+14.02).R	Y	44.82	1302.5743	9	-1.0	652.2904	2	58.26	40	F40:14686	MAX09905_AndrejP_gel_F09_RB1_01_19190.mgf		0		0	0	0	0	102	110	Oxidation (M); Methylation(KR)	M1:Oxidation (M):1000.00;R9:Methylation(KR):1000.00	PEAKS PTM
R.NLEVGIPALLQDDIK.L	Y	39.05	1636.9036	15	5.0	819.4594	2	56.58	17	F17:13643	MAX09858_AndrejP_gel_SA09_BB1_01_19064.mgf	0			0	0	0	0	382	396			PEAKS DB
K.AALEM(+15.99)LK(+14.02).R	Y	36.86	804.4415	7	-33.5	403.2124	2	12.57	40	F40:2050	MAX09905_AndrejP_gel_F09_RB1_01_19190.mgf		0		0	0	0	0	471	477	Oxidation (M); Methylation(KR)	M5:Oxidation (M):1000.00;K7:Methylation(KR):1000.00	PEAKS PTM
R.NLEVGIPALLQ(+.98)DDIK.L	Y	35.58	1637.8876	15	4.6	819.9502	2	57.60	39	F39:14363	MAX09905_AndrejP_gel_F09_RB1_01_19189.mgf		0		0	0	0	0	382	396	Deamidation (NQ)	Q11:Deamidation (NQ):112.07	PEAKS DB
R.NLEVGIPALLQDDIK(+14.02).L	Y	34.94	1650.9192	15	4.7	826.4669	2	57.82	17	F17:13966	MAX09858_AndrejP_gel_SA09_BB1_01_19064.mgf	0			0	0	0	0	382	396	Methylation(KR)	K15:Methylation(KR):1000.00	PEAKS PTM
**total 7 peptides**																							

UGZ35775.1

back to list

| Protein Coverage | Supporting Peptides |

Protein Coverage:

```
  1 MVTIKCVKAR QIYDSRGNPT VEADVHLDDG TFARAAVPSG ASTGIYEALE LRDGGSDYMG KGVQKAVNNV NEIIGPALVG
 81 KDPTQQTAID NFMVQQLDGT VNEWGWCKQK LGANAILAVS LAVCKAGAQV KKTPLYKHIA ELAGNKNLVL PVPAFNVING
161 GSHAGNKLAM QEFMILPVGA SSFKEAMKMG SEVYHHLKSV IKKKYGQDAT NVGDEGGFAP NIQENKEGLE LLKTAIEKAG
241 YTGKVVIGMD VAASEFYKDD KTYDLNFKEE NNDGSQKISG NALKDLYKSF VSEYPIVSIE DPFDQDDWEH YAKMTAEIGD
321 KVQIVGDDLL VTNPKRVQKA IDEKTCNALL LKVNQIGSVT ESIEAVKMSK QAGWGVMASH RSGETEDTFI ADLSVGLSTG
401 QIKTGAPCRS ERLAKYNQLL RIEEELGAEA VYAGAKFRQP VEPY
```

f Formylation (+27.99)

184

Supporting Peptides:

Peptide	Uniq	-10lgP	Mass	Length	ppm	m/z	z	RT	Fraction	Scan	Source File	Area AndrejP_JAtreatment	Area AndrejP_Feeding	Area AndrejP_CNTR	#Feature	#Feature AndrejP_JAtreatment	#Feature AndrejP_Feeding	#Feature AndrejP_CNTR	Start	End	PTM	AScore	Found By
R.AAVPSGASTGIYEALELR.D	Y	53.43	1803.9366	18	0.5	902.9722	2	55.25	56	F56:13058	MAX09913_AndrejP_gel_C06_RC6_01_19209.mgf			0	0	0	0	0	35	52			PEAKS DB
K.LAMQEFMILPVGASSFK(+27.99).E	Y	53.16	1895.9525	17	7.1	948.9861	2	58.46	56	F56:14063	MAX09913_AndrejP_gel_C06_RC6_01_19209.mgf			0	0	0	0	0	168	184	Formylation	K17:Formylation:129.22	PEAKS PTM
K.LAM(+15.99)QEFMILPVGASSFK(+27.99).E	Y	52.24	1911.9474	17	7.4	956.9839	2	57.60	56	F56:13784	MAX09913_AndrejP_gel_C06_RC6_01_19209.mgf			0	0	0	0	0	168	184	Formylation	M3:Oxidation (M):82.63;K17:Formylation:166.52	PEAKS PTM
**total 3 peptides**																							

KNA11181.1

back to list

| Protein Coverage | Supporting Peptides |

Protein Coverage:

1 MAAGVGGAAT EATKRDVTTV VDRREQHHLR RENDGVLMQI FVKTLTGKTI TLEVESSDTI DNVKTK**IQDK EGIPPDQQR**L

81 IFAGKQLEDG RTLADYNIQK **ESTLHLVLR**L RGGMQIFVKT LTGKTITLEV ETSDTIENVK TKIHDKEGIA PDQQRLIFAG

161 KQLEDGRTLA DYNIQKESTL HLVLRLRGGM QIFVKTLTGK TITLEVESSD TIDNVKTKIQ DKEGIYPTRS AEDYLCWEAI

241 GGWENFGRL

Supporting Peptides:

Peptide	Uniq	-10lgP	Mass	Length	ppm	m/z	z	RT	Fraction	Scan	Source File	Area AndrejP_JAtreatment	Area AndrejP_Feeding	Area AndrejP_CNTR	#Feature	#Feature AndrejP_JAtreatment	#Feature AndrejP_Feeding	#Feature AndrejP_CNTR	Start	End	PTM	AScore	Found By
K.ESTLHLVLR.L	Y	46.83	1066.6135	9	-2.6	534.3091	2	31.17	65	F65:7087	MAX09918_AndrejP_gel_C11_RD3_01_19218.mgf	0	0	0	0	0	0	0	101	109			PEAKS DB
K.IQDKEGIPPDQQR.L	Y	36.42	1522.7739	13	-1.8	508.5950	3	11.60	24	F24:1788	MAX09897_AndrejP_gel_F01_RA1_01_19174.mgf		0		0	0	0	0	67	79			PEAKS DB
total 2 peptides																							

XP_021852453.1

back to list

| Protein Coverage | Supporting Peptides |

Protein Coverage:

C Carbamidomethylation (+57.02)

1 MSSVDTVLIA KLHTLILSII IPIFLLLSNT VAVPRSYSPN IHFRSSQIEN NEIPHFRTLN RRQLIDCPFP NPYLEITVNT

139 C

81 VSPLPDEIYI NVTVSGVLKT SPKYWVAMVS PSHSSVKYCP LKDLLYKETG DFSD**LPLLCH YPVK**AAYLKN DPEFLPCKNK

161 ECKKRGVDGN CMVETCSASL TFHVINIRTD IQFVLFDNGF KAPCILKGTT PLPFANPNKP LYGHISSVNS AATSMRVTWV

241 SGSIEPQQVE YGNGKSVTST VGTFTQDDMC GDIAGKSPAE DFGWHDPGYI HTAVITGLQP SANFSYKYGS GAAGWSDNIQ

321 FRTPPAGGAN DLKFLAFGDM GKAPRDSTLE HYIQPGSLSV VAAMAEEVAT GKVDSIFHIG DISYATGFLV EWDYFLHLIT

401 PLASRVSYMT AIGNHERDYV NSGSVYITPD SGGECGVPYE SYFQMPTSLK **DKPWYSIEQG PVHF**TVISTE NNWRPGSEQY

481 NWMKSDMASV DRRRTPWLVF TGHRPMYSSA KSALPSVDLS FVKYIEPLLV QNM**VDLVLFG HVHNYER**TCA IYRGKCMSKP

561 LKDENGIDTF DNSKYKAPVQ AVIGMAGFSL DEFSNFAPDW SLQRINKFGF ARIHVTRKEL KLEFVNSNTR QVEDSFRIIK

Supporting Peptides:

Peptide	Uniq	-10lgP	Mass	Length	ppm	m/z	z	RT	Fraction	Scan	Source File	Area AndrejP_JAtreatment	Area AndrejP_Feeding	Area AndrejP_CNTR	#Feature	#Feature AndrejP_JAtreatment	#Feature AndrejP_Feeding	#Feature AndrejP_CNTR	Start	End	PTM	AScore	Found By
M.VDLVLFGHVHNYER.T	Y	41.53	1696.8685	14	0.4	566.6287	3	42.84	42	F42:10364	MAX09906_AndrejP_gel_F10_RB2_01_19192.mgf		0	0	0	0	0	0	534	547			PEAKS DB
D.LPLLC(+57.02)HYPVK.A	N	40.49	1238.6846	10	1.7	620.3524	2	25.97	21	F21:5625	MAX09860_AndrejP_gel_SA11_BB3_01_19068.mgf	0	0		0	0	0	0	135	144	Carbamidomethylation	C5:Carbamidomethylation:1000.00	PEAKS DB
K.DKPWYSIEQGPVHF.T	N	35.62	1701.8151	14	0.1	568.2773	3	54.87	42	F42:13835	MAX09906_AndrejP_gel_F10_RB2_01_19192.mgf		0		0	0	0	0	451	464			PEAKS DB
total 3 peptides																							

XP_048495078.1

back to list

| Protein Coverage | Supporting Peptides |

Protein Coverage:

C Carbamidomethylation (+57.02)

1 MEIPGGIKLT VLLMLVMINY GRCSSSISSS MNNSSEKAHK VNNVRHDSYR RFLLSNGLAL TPPMGWNSWN HFGCGVTEKI

81 VKETADALVS TGLSKLGYKY VNIDDCWAEL TRDDKGNFVP KESTFPSGMK ALADYVHSKG LNIGIYSAAG YKTCSQKMPG

161 SLGFEEQDAK TFASWGIDYL KYDNCNNDRS KPTVRYPVMT RALMKAGRPI FFSLCEWGDM HPALWGYQVG NSWRTTDDIS

301 C

241 DNWESMVTRA DMNEFYADYA RPGGWNDPDM LEVGNGGMSK DEYI**VHFSIW AISKAPLLIG CDVR**NMTQET MGIIGNEEVI

321 AVNQDPLGIQ AKKVRMEGGL EVWAGPLTNY TVALLIVNRG AVTMSITAHW DDIGLPMDSI VEARDLWEHK TLEQRFVQNL

401 TASVDSHGSK MYVLKPIA

Supporting Peptides:

Peptide	Uniq	-10lgP	Mass	Length	ppm	m/z	z	RT	Fraction	Scan	Source File	Area AndrejP_JAtreatment	Area AndrejP_Feeding	Area AndrejP_CNTR	#Feature	#Feature AndrejP_JAtreatment	#Feature AndrejP_Feeding	#Feature AndrejP_CNTR	Start	End	PTM	AScore	Found By
K.APLLIGC(+57.02)DVR.N	Y	53.85	1112.6012	10	-2.6	557.3044	2	24.67	15	F15:5013	MAX09857_AndrejP_gel_SA08_BA8_01_19062.mgf	0	0	0	0	0	0	0	295	304	Carbamidomethylation	C7:Carbamidomethylation:1000.00	PEAKS DB
I.VHFSIWAISK.A	Y	37.04	1186.6499	10	-0.3	594.3282	2	52.65	37	F37:12560	MAX09904_AndrejP_gel_F08_RA8_01_19187.mgf		0	0	0	0	0	0	285	294			PEAKS DB
total 2 peptides																							

MCK0622870.1

back to list

| Protein Coverage | Supporting Peptides |

Protein Coverage:

1 MGLSLVPYLF IFIAISSSLA LSYASTIDDQ TKVDDDDECP TCEAGLYCFN CRLGSTGPKC VRTTTTDPFQ LVNNSLPFNK

81 YAYLTTHNAY AIEGAPSRTG VPRFTFTNQQ DNVTEQLQNG VRALMLDTYD FR**GDVWLCHS FGGK**CHDYTA FEPATDTLKE

161 IEAFMSSNPG EIVTLILEDY VEEPKGLSKV FTDAGLMKYW FPVSRMPKNG EDWPLVSDMV AKNQRLLVFT SIQSKEASEG

241 IAYQWNYMVE NQYGDGGMKP EQCTNRAESA PLNDTTN**SLV LVNYFR**SIPI KQFSCVDNSA SLTAMLRTCH DAAANRWANF

321 VAVDHYKRSE GGGAFQAVDM LNGELLCGCN DVHACVVSPT S

C Carbamidomethylation (+57.02)
d Deamidation (NQ) (+0.98)
f Formylation (+27.99)

Supporting Peptides:

Peptide	Uniq	-10lgP	Mass	Length	ppm	m/z	z	RT	Fraction	Scan	Source File	Area AndrejP_JAtreatment	Area AndrejP_Feeding	Area AndrejP_CNTR	#Feature	#Feature AndrejP_JAtreatment	#Feature AndrejP_Feeding	#Feature AndrejP_CNTR	Start	End	PTM	AScore	Found By
R.GDVWLC(+57.02)HSFGGK.C	Y	47.58	1361.6187	12	-2.5	681.8115	2	26.04	18	F18:5462	MAX09858_AndrejP_gel_SA09_BB1_01_19065.mgf	0		0	0	0	0	0	133	144	Carbamidomethylation	C6:Carbamidomethylation:1000.00	PEAKS DB
N.SLVLVNYFR.S	Y	44.49	1109.6233	9	-2.1	555.8152	2	47.12	17	F17:11117	MAX09858_AndrejP_gel_SA09_BB1_01_19064.mgf	0	0	0	0	0	0	0	278	286			PEAKS DB
N.S(+27.99)LVLVNYFR.S	Y	42.61	1137.6182	9	0.0	569.8140	2	58.83	61	F61:15246	MAX09916_AndrejP_gel_C09_RD1_01_19214.mgf		0	0	0	0	0	0	278	286	Formylation	S1:Formylation:1000.00	PEAKS PTM
N.SLVLVN(+.98)YFR.S	Y	40.95	1110.6073	9	-0.1	556.3086	2	53.92	61	F61:13644	MAX09916_AndrejP_gel_C09_RD1_01_19214.mgf	0	0	0	0	0	0	0	278	286	Deamidation (NQ)	N6:Deamidation (NQ):1000.00	PEAKS DB
total 4 peptides																							

XP_021719798.1

back to list

| Protein Coverage | Supporting Peptides |

Protein Coverage:

1 MSNRKILLIV FSLLAVISAV SAAGKRTGAV LGGYKPIKDI NDPYVQEVAK FAVAKHNEEA NSHLGIVRIV K**GESQVVAGT**

81 **NYR**FLIAADD HQQYETVVYD KPWQHFRSLT YFKKVSA

Supporting Peptides:

Peptide	Uniq	-10lgP	Mass	Length	ppm	m/z	z	RT	Fraction	Scan	Source File	Area AndrejP_JAtreatment	Area AndrejP_Feeding	Area AndrejP_CNTR	#Feature	#Feature AndrejP_JAtreatment	#Feature AndrejP_Feeding	#Feature AndrejP_CNTR	Start	End	PTM	AScore	Found By
K.GESQVVAGTNYR.F	Y	68.69	1279.6156	12	3.8	640.8170	2	14.97	30	F30:2670	MAX09900_AndrejP_gel_F04_RA4_01_19180.mgf		0	0	0	0	0	0	72	83			PEAKS DB
K.GE(+14.02)SQVVAGTNYR.F	Y	52.41	1293.6313	12	3.9	647.8253	2	18.56	29	F29:3628	MAX09900_AndrejP_gel_F04_RA4_01_19179.mgf		0		0	0	0	0	72	83		E2:Methylation(others):8.69	PEAKS PTM
total 2 peptides																							

XP_021769989.1

back to list

| Protein Coverage | Supporting Peptides |

Protein Coverage:

1 MGLTFTKLFS GLFSKKEMR**I LMVGLDAAGK** TTILYKLKLG EIVTTIPTIG FNVETVEYKN ISFTVWDVGG QDKIRPLWRH

o Oxidation (M) (+15.99)

81 YFQNTQGLIF VVDSNDRDRV VEARDELHRM LNEDELR**DAV LLVFANK**QDL PNAMNAAEIT DKLGLHSLHQ RHWYIQSTCA

161 TSGEGLYEGL DWLSNNIASK V

Supporting Peptides:

Peptide	Uniq	-10lgP	Mass	Length	ppm	m/z	z	RT	Fraction	Scan	Source File	Area AndrejP_JAtreatment	Area AndrejP_Feeding	Area AndrejP_CNTR	#Feature	#Feature AndrejP_JAtreatment	#Feature AndrejP_Feeding	#Feature AndrejP_CNTR	Start	End	PTM	AScore	Found By
R.DAVLLVFANK.Q	Y	42.44	1088.6229	10	3.1	545.3203	2	52.45	29	F29:13113	MAX09900_AndrejP_gel_F04_RA4_01_19179.mgf		0	0	0	0	0	0	118	127			PEAKS DB
R.ILM(+15.99)VGLDAAGK.T	Y	38.99	1102.6056	11	2.0	552.3110	2	30.70	29	F29:7374	MAX09900_AndrejP_gel_F04_RA4_01_19179.mgf		0	0	0	0	0	0	20	30	Oxidation (M)	M3:Oxidation (M):1000.00	PEAKS DB
R.ILMVGLDAAGK.T	Y	36.36	1086.6107	11	-4.1	544.3069	2	40.14	66	F66:9301	MAX09918_AndrejP_gel_C11_RD3_01_19219.mgf			0	0	0	0	0	20	30			PEAKS DB
total 3 peptides																							

XP_021859482.1

back to list

| Protein Coverage | Supporting Peptides |

Protein Coverage:

```
  1 MSKGKHYNFL LLLSLYLCLV CNILILTPTN ASDQVTNLYK LLKSKRSKNP SLAASWVTLP AQIKESRVHI QPQDGLMEAD
 81 KITSLPGQPD GVDFDHYSGY VTVDPAVEKA LFYYFAESPT NSSSKPLVLW LNGGPGCSSF GNGGLLELGP FRVKSDGKTL
161 IRNPYAWNNV ANVMFLESPA GVGFSYGNVT IIDKQTGDIS TAKDAYTFLA NWFERFPHYK HRDFYLTGES YAGHYVPQLA
241 YTILHNNNNT NHTFINLKGI AIGNAWIDDA TNTQGLYDYL WNHATHSDET HAKINKFCNF SETIPSKECD QYCNKAWEEA
321 GNIDIYNIYA PVCLKSGNSK AHILHSVEVF DPCSSTYVDT YLNTKQVQQA LHVKPTKWIS CSGFAWQDTP STVLPVIQKL
401 MESNISVWLY SGDVDGRVPV TSTKYSLNTL KLPIKTDWRP WYDTDNQVGG YVVGYEGLVF VTVRGAGHLV PSYQPQRALT
481 IISSFLQGTL PPPKSS
```

d Deamidation (NQ) (+0.98)

Supporting Peptides:

Peptide	Uniq	-10lgP	Mass	Length	ppm	m/z	z	RT	Fraction	Scan	Source File	Area AndrejP_JAtreatment	Area AndrejP_Feeding	Area AndrejP_CNTR	#Feature	#Feature AndrejP_JAtreatment	#Feature AndrejP_Feeding	#Feature AndrejP_CNTR	Start	End	PTM	AScore	Found By
K.YSLN(+.98)TLKLPIK.T	Y	54.99	1289.7594	11	-21.8	645.8752	2	47.64	22	F22:11893	MAX09860_AndrejP_gel_SA11_BB3_01_19069.mgf	0	0		0	0	0	0	425	435	Deamidation (NQ)	N4:Deamidation (NQ): 1000.00	PEAKS DB
total 1 peptides																							

XP_021758194.1

back to list

| Protein Coverage | Supporting Peptides |

Protein Coverage:

```
  1 MEHKIFTSRP PSKLIILFLF LFCSSISFTK AIKPPPLLDN FNSNKLYFNS RFYSLYDTSK YGQFQLFNGL ARTPQMGWNS
 81 WNYFACNINE TVIKETADAL VSTGLADLGY VFVNIDDCWS RLTRNLEDQL VPDPKTFPSG IKALADYVHD KGLKLGIYSD
161 AGAFTCQVRP GSIFHEKDDA QIFASWGVDY LKYDNCFNLG IPPKKRYPPM HDALNATERP IFYSLCEWGV DDPALWAGEV
241 GNSWRTTDDI NDSWASMTTI ADLNDKWASY AGPGGWNDPD MLEVGNGGMT YNEYRAHFSI WALMKAPLLI GCDIRNMTAE
321 TLKILSNTEV IGVNQDPLGV QGRKVHASGK DGCQQVWAGP LSGNRLAVAL WNRCSSAALI TAEWNVLGLQ SSISVSIRDL
401 WKHNLVASDA VSSFGASVES HDCAMYIFTP KMSESYSAST DSTATSGKVF SPPIWRPNDR KSKKENVASF RLYTIEMKIK
481 MDRHLELLEQ GKGQEQERKR GNK
```

c Carbamidomethylation (+57.02)

Supporting Peptides:

Peptide	Uniq	-10lgP	Mass	Length	ppm	m/z	z	RT	Fraction	Scan	Source File	Area AndrejP_JAtreatment	Area AndrejP_Feeding	Area AndrejP_CNTR	#Feature	#Feature AndrejP_JAtreatment	#Feature AndrejP_Feeding	#Feature AndrejP_CNTR	Start	End	PTM	AScore	Found By
K.APLLIGC(+57.02)DIR.N	Y	53.12	1126.6168	10	-2.7	564.3110	2	39.80	39	F39:9400	MAX09905_AndrejP_gel_F09_RB1_01_19189.mgf	0	0	0	0	0	0	0	306	315	Carbamidomethylation	C7:Carbamidomethylation:1000.00	PEAKS DB
total 1 peptides																							

MCK0601578.1

back to list

| Protein Coverage | Supporting Peptides |

Protein Coverage:

```
  1 MATQMSKKRK FVADGVFFAE LNEVLTRELA EDGYSGVEVR VTPMRTEIII RATRTQNVLG EKGRRIRELT SVVQKRFKFP
 81 ENSVELYAEK VNNRGLCAIA QAESLRYKLL GGLAVRRACY GVLRFVMESG AKGCEVIVSG KLRAQRAKSM KFKDGYMISS
161 GQPVNEYIDS AVRHVLLRQG VLGIKVKIML EWDPKGKQGP PTPLPDLVTI HPPKEEEVLI QPPVLTTNIE VEVPVPVPVA
```

c Carbamidomethylation (+57.02)

Supporting Peptides:

Peptide	Uniq	-10lgP	Mass	Length	ppm	m/z	z	RT	Fraction	Scan	Source File	Area AndrejP_JAtreatment	Area AndrejP_Feeding	Area AndrejP_CNTR	#Feature	#Feature AndrejP_JAtreatment	#Feature AndrejP_Feeding	#Feature AndrejP_CNTR	Start	End	PTM	AScore	Found By
R.GLC(+57.02)AIAQAESLR.Y	Y	51.57	1287.6605	12	-2.1	644.8318	2	38.16	65	F65:8846	MAX09918_AndrejP_gel_C11_RD3_01_19218.mgf			0	0	0	0	0	95	106	Carbamidomethylation	C3:Carbamidomethylation:1000.00	PEAKS DB
total 1 peptides																							

XP_021743448.1

back to list

| Protein Coverage | Supporting Peptides |

Protein Coverage:

```
  1 MKIGAKFAFL CSLLVGICCI QSLIIGCQAG RVVSKLSINP VLKVKAVNLG GWLLTEGWIK PSLFDGIPNK DLLDGTQIQL
 81 KSVTIGKYVC AENGGGNILV ANRISASGWE TFKLWRIDEN TFNFRVFSNQ FAGVQAGGGS PTVVTVASTP GASETFKIVR
161 NPNDKNRVRI QAPSGLFLQA KTQDQVTADY AGDGGWGDDN PSVFVMTIVA TLQGEFQVTN GYGPDKAPEV MREHWNTFIV
241 EDDFRFISQN GLNAVRIPVG WWIASDPTPP KPFVGGSLQA LDNAFTWAQK YGIQVIVDLH AAPGSQNGNE HSGTRDGSLE
321 WGKTDATVQQ SVAVIDFLAS RYAQKSNLLA IELINEPVAP AVTLDTLKSY YQQGYDTVRK YSSSAYVIMS NRLGPIDARE
401 LFPLASGKQL TVVDVHYYNL FSSIFDNMSV QQNIDFINNN RSSDLGYITT SDGPLTFVGE WVAEWQVNGA TKEDFQNFAN
481 AQLQVYGRAT FGWAYWTYKN VNNHWSLQWM IQNGYITLTN
```

Supporting Peptides:

Peptide	Uniq	-10lgP	Mass	Length	ppm	m/z	z	RT	Fraction	Scan	Source File	Area AndrejP_JAtreatment	Area AndrejP_Feeding	Area AndrejP_CNTR	#Feature	#Feature AndrejP_JAtreatment	#Feature AndrejP_Feeding	#Feature AndrejP_CNTR	Start	End	PTM	AScore	Found By
Q.VIVDLHAAPGSQN(+.98)GNEHSGTR.D	Y	50.75	2159.0354	21	0.5	540.7642	4	19.61	61	F61:3961	MAX09916_AndrejP_gel_C09_RD1_01_19214.mgf		0	0	0	0	0	0	295	315		N13:Deamidation (NQ):12.28	PEAKS DB
Q.VIVDLHAAPGSQNGNEHSGTR.D	Y	37.44	2158.0515	21	-0.6	540.5173	4	15.27	17	F17:2501	MAX09858_AndrejP_gel_SA09_BB1_01_19064.mgf	0			0	0	0	0	295	315			PEAKS DB
**total 2 peptides**																							

BAM28610.1

back to list

| **Protein Coverage** | **Supporting Peptides** |

Protein Coverage:

d Deamidation (NQ) (+0.98)

```
  1 MKTHYSSAIL PIVTLFVFLS INPSHGSGIA VYWGQNGNEG TLSDTCATGN YNYVLVSFLT TFGNGQTPVL NLAGHCDPSS
 81 NGCTGLSTDI TSCQNQGIKV LLSLGGASGS YSLVSTDDAN QVAAYLWNNY LGGQSDSRPL GAAVLDGIDF DIESGSDNYW
161 GDLASALKGY SQSVLVSAAP QCPYPDAHLD QAIATGIFDY VWVQFYNNEQ CEYVSDDTNL LSAWNQWTSS QANVVFLGLP
                                                282
                                                 d
241 ASTDAASSGY IPPDVLTSQV LPSIKASSKY GGVMLWSKYY DNGYSSAIKD SV
                                                 d
```

Supporting Peptides:

Peptide	Uniq	-10lgP	Mass	Length	ppm	m/z	z	RT	Fraction	Scan	Source File	Area AndrejP_JAtreatment	Area AndrejP_Feeding	Area AndrejP_CNTR	#Feature	#Feature AndrejP_JAtreatment	#Feature AndrejP_Feeding	#Feature AndrejP_CNTR	Start	End	PTM	AScore	Found By
K.YYDN(+.98)GYSSAIK.D	Y	48.19	1280.5560	11	0.9	641.2831	2	21.03	55	F55:4132	MAX09913_AndrejP_gel_C06_RC6_01_19208.mgf			0	0	0	0	0	279	289	Deamidation (NQ)	N4:Deamidation (NQ): 1000.00	PEAKS DB
**total 1 peptides**																							

XP_021730180.1

back to list

| **Protein Coverage** | **Supporting Peptides** |

Protein Coverage:

```
  1 MAFSVKIEPS LSLYSRKTTS RFPSHPLRSF SFSLPLQPSH NFISLTKMLS HSHSAIKASS SSPSTSTILE SQNFSIKMVP
 81 TKPYDGQKTG TSGLRKKVKV FMQENYLANW IQALFNSLPS VDYVNGVLVL GGDGRYFNKE AAQTIIKIAA ANGVAKILVG
161 QDGILSTPAV SAVIRKRKAN GGFIMSASHN PGGPDYDWGI KFNYSSGQPA PESITDKIYG NTLSISEIKM ATVPDVDLSC
241 VGVTKFGNFV VEVVDPVADY MELMESVFDF QLIGSLLSRS EFRFTFDAMH AVTGAYAKPI FVEKLGASPE SITNGVPLED
321 FGHGHPDPNL TYAKDLVDIM YGENGPDFGA ASDGDGDRNM ILGRSFFVTP SDSVAIIAAN ALEAIPYFRD GIKGLARSMP
401 TSGALDRVAE KLKLPFFEVP TGWKFFGNLM DSGKLSICGE ESFGTGSDHI REKDGIWAVL AWMSIIAYRN KETKPGERLV
481 SVSDVVKEYW ATYGRNFFSR YDYEECESEG ANKMVDHLRD LISRSREGDR YGEYVLRFAD DFSYTDPVDG SVASKQGVRF
561 VFSDGSRIIF RLSGTGSAGA TIRVYIEQFE PDASKHDLDA QIALKPLIDL ALSISKLKDF TGREKPTVIT
```

Supporting Peptides:

Peptide	Uniq	-10lgP	Mass	Length	ppm	m/z	z	RT	Fraction	Scan	Source File	Area AndrejP_JAtreatment	Area AndrejP_Feeding	Area AndrejP_CNTR	#Feature	#Feature AndrejP_JAtreatment	#Feature AndrejP_Feeding	#Feature AndrejP_CNTR	Start	End	PTM	AScore	Found By
R.LSGTGSAGATIR.V	Y	45.74	1089.5778	12	-0.6	545.7935	2	13.32	55	F55:2176	MAX09913_AndrejP_gel_C06_RC6_01_19208.mgf			0	0	0	0	0	572	583			PEAKS DB
**total 1 peptides**																							

APO15851.1

back to list

| **Protein Coverage** | **Supporting Peptides** |

Protein Coverage:

1 MGKTIQVSGF PSTVSAEAVK TYLEQYTGEK SVYALKIRQF KTGGKRSYAI VQFTSAGAAE RILSLGLPPK KLWYGSSYLQ

V->P

81 VRVMERDIVP KPRMYQHTMT NVTLHFGCQI SNDKYLWFSK GQDVSLLFGY GMRKLYFFLR HIRKEYKLEL SYESIWQIEL

161 RRPRSIRLKY LLIQLFRVPQ VYEKVEHSFG QLFENNFFRD QQDDQWIRTT DFTISCIGES SVLCLELPSD CQLPNFK**EYF**

241 **VYYKEENGQF IVER**GSTFSN NLKLVPIVAP PRGSSLPFDI LFKVNLLVQN GCLAGPNLDD TFFRLVDPVR ISKPCIEYAL

321 ERLFQVKECC YNPVGWLSEQ YQKYLKSMRR PEKPAISLDE GLVYVRRVQV TPCKVYFCGP EANVSNRVLR HYPDDIDNFL

401 RISFLDEDLE KLHSADLSAR LSSESAAFDR RTEIDKRIRL TLRNGILIGD KRFEFLAFSS SQLRDNSAWM FASRPGLSAA

481 DIRSWMGDFS GIRNVAKYAA RLGQSFSSST ETLTVAKHEV EMIPDVERND YVFSDGIGKI SADFAHKVAI KCGFKSSSPS

561 CFQIRYGGFK GVVAVDPSSS KKLSLRKSMC KYKSENEKLD VLAYSKYQPC YLNRQLITLL STLGVKDRVF EKKQREALNQ

641 LDAILTDPLK AQEALELMCP GEITNILKEM LKCGYKPDTE PFLSMMLQAF RSSKLQELRT KSRIFVPRGR AMMGCLDETK

721 ALEYGQVFVQ VSGARFRNVG NELLTHVGTE YEPCNYVVKG KVVVAKNPCL HPGDVRVLMA VDVPSLHHMV DCVIFPQKGN

801 RPHPNECSGS DLDGDIYFVC WDSDLIPPRQ VAPMDYSPAK CEELDHDVTI EEVMEYFANY IINDSLGIIA NAHTAFADKE

881 PGKAMSSSCI ELAKLFSIAV DFPKTGVPAV IPRNLYVKEY PDFMEKPDKP TYESVNVIGK LFREVKERSP QAPSIRRFTL

961 EIAMRSYDRD MEYDGFEDHL ADAHYYKSQY DFKLGNLMEY YGIKTEAEIL SGNIMRMSNS FDRRKDVEAI TMAVRSLRKE

1041 ARTWFNEKGT DSSSGADNMY AKASAWYHVT YHPSYWGSYN EGMNRDHFLS FPWSVYDRLI TIKKNRMTAS SAELSSLVHR

1121 FGKGFSMK

Supporting Peptides:

Peptide	Uniq	-10lgP	Mass	Length	ppm	m/z	z	RT	Fraction	Scan	Source File	Area AndrejP_JAtreatment	Area AndrejP_Feeding	Area AndrejP_CNTR	#Feature	#Feature AndrejP_JAtreatment	#Feature AndrejP_Feeding	#Feature AndrejP_CNTR	Start	End	PTM	AScore	Found By
K.EYFP(sub V)YYKEENGQFIVER.G	Y	43.42	2210.0320	17	35.3	737.7136	3	62.50	11	F11:14655	MAX09855_AndrejP_gel_SA06_BA6_01_19058.mgf	0			0	0	0	0	238	254			SPIDER
total 1 peptides																							

KNA06751.1

back to list

| Protein Coverage | Supporting Peptides |

Protein Coverage:

1 MAAHSSLLRS APSSSLFGSS SFSRSSDLSK AISSHNISSL KIQTNVFGAA VHCGSSSIRK CRSTNVQPIK ATATVIPPAV

C Carbamidomethylation (+57.02)

81 QESRSGGKTK VGINGFGRIG RLVLRVAISR DDLEVVAVND PFVDAKYMAY MFKYDSTHGP FKGTINVVDD STIEINGKQV

161 KVTNKRNPEE IPWGDFGVEY VVESSGVFTT LEKAAAHKKG GARKVVISAP SADAPMFVIG VNEKTYKPNM DVVSNASCTT

241 NCLAPLAKVV HEEFGILEGL MTTVHATTAT QKTVDGPSMK DWRGGRGASQ NIIPSSTGAA KAVGKVLPEL NGKLTGMAFR

333

321 **VPTANVSVVD LTCR**LQKEAS YEDVKAAIKF ASEGPLKGIL GYTEDDVVSN DFTGDTRSSI FDAKAGIALS SSFVKLVSWY

401 DNEWGYSNRV LDLIEHMALV AAI

Supporting Peptides:

Peptide	Uniq	-10lgP	Mass	Length	ppm	m/z	z	RT	Fraction	Scan	Source File	Area AndrejP_JAtreatment	Area AndrejP_Feeding	Area AndrejP_CNTR	#Feature	#Feature AndrejP_JAtreatment	#Feature AndrejP_Feeding	#Feature AndrejP_CNTR	Start	End	PTM	AScore	Found By
R.VPTANVSVVDLTC(+57.02)R.L	Y	42.86	1529.7871	14	-2.9	765.8959	2	29.64	15	F15:6301	MAX09857_AndrejP_gel_SA08_BA8_01_19062.mgf	0	0	0	0	0	0	0	321	334	Carbamidomethylation	C13:Carbamidomethylation:1000.00	PEAKS DB
total 1 peptides																							

XP_021867395.1

back to list

| Protein Coverage | Supporting Peptides |

Protein Coverage:

1 MERRRVTFAA TFLCLLLLLL PELNVVANAQ DGAATEPVTP ALFIFGDSLI DDGNNNYLVT AAKANYFPYG IDSGGPTGRF

81 SNGLTVVDYG AQYLGLPIIP PYFSLTSFGK HILRGINYAS AAAGILDETG RHYGQRTSLN GQISQFQETV ALKLPLLFQN

161 QDELTQYLAK SVFLIDIGGN DYLNNYLQPE RYDSSRIYDG EGFADLLMNT LTSQITRLYN IGARKMVLVG TGPLGCIPSF

241 LSKSSDNSCV QSVNNLIIPF NSRLTQITNT LNQSLPGSFF VYQNTYDFFY DVIKTPSK**YG FTVSTK**ACCG NGRAGGELTC

321 LPLQQPCADR NQYVFWDSFH PTQAVNAIIA RRGYSQYATD CIPISIYQLA QL

Supporting Peptides:

Peptide	Uniq	-10lgP	Mass	Length	ppm	m/z	z	RT	Fraction	Scan	Source File	Area AndrejP_JAtreatment	Area AndrejP_Feeding	Area AndrejP_CNTR	#Feature	#Feature AndrejP_JAtreatment	#Feature AndrejP_Feeding	#Feature AndrejP_CNTR	Start	End	PTM	AScore	Found By
K.YGFTVSTK.A	Y	41.83	901.4545	8	-2.1	451.7317	2	15.97	16	F16:2680	MAX09857_AndrejP_gel_SA08_BA8_01_19063.mgf	0			0	0	0	0	299	306			PEAKS DB
total 1 peptides																							

KAH9604880.1

back to list

| Protein Coverage | Supporting Peptides |

Protein Coverage:

1 MASSSLLRSA PSISLSDNLS SQLPAFRQSH LSQVINVGFN TQQVRSSFMS GAALHNASSF RKSKPFCIQP IKATAIEIPP

C Carbamidomethylation (+57.02)

81 TFQQSKSGGK TKVGINGFGR IGRLVLRIAT SRDDLEVVAV NDPFIDAKYM AYMFKYDSTH GVFKGTIEVI DDSTLEINGK
161 QVKVTSKRYD FDTRFIVIWN PEEIPWGNYG VEYVVESSGV FTTLEKAAAH KKGGAKKVVI SAPSADAPMF VVGVNERTYK
241 ANMDVVSNAS CTTNCLAPLA KVVHEEFGIV EGLMTTVHAT TATQKTVDGP SMKDWRGGRG AAQNIIPSST GAAKAVGKVL

346 C

321 PELNGKLTGM AFR**VPTPNVS VVDLTCR**LQK SATYEDVKAA VKFASEGPLN GILGYTEDDV VSNDFLGDSR SSIFDAKAGI
401 GLSSSFVKLV SWYDNEWGYS NRVLDLIEHM ALVAAS

Supporting Peptides:

Peptide	Uniq	-10lgP	Mass	Length	ppm	m/z	z	RT	Fraction	Scan	Source File	Area AndrejP_JAtreatment	Area AndrejP_Feeding	Area AndrejP_CNTR	#Feature	#Feature AndrejP_JAtreatment	#Feature AndrejP_Feeding	#Feature AndrejP_CNTR	Start	End	PTM	AScore	Found By
R.VPTPNVSVVDLTC(+57.02)R.L	Y	41.15	1555.8029	14	0.9	778.9060	2	43.61	55	F55:9982	MAX09913_AndrejP_gel_C06_RC6_01_19208.mgf			0	0	0	0	0	334	347	Carbamidomethylation	C13:Carbamidomethylation:1000.00	PEAKS DB
total 1 peptides																							

ABB89525.1

back to list

| **Protein Coverage** | **Supporting Peptides** |

Protein Coverage:

1 MLIALLLGIL FATINTRASQ IGTCFGMMAN NLPPLPDVVA QYNQYSIERM RIYGPVSSLS QALSGSGIEL VLGVPNQDLQ
81 AIASSQSNAN SWVQDNIGAY PNVNFR**YLAV GNEIRPN**LNN GAAQYAQCVL PAMQNLQNAI NQMGYGGRVK VSTAVEMGVA
161 INTYPPSAGQ FDPSISYFIN PIVRFMRDNG SPLLLNCYPY FAYAYSSNID LSYALFTSPG TVVQDGQYAY QNLFDAMVDS
241 IYSALEKADC GSVVIVVSES GWPTMGGKGT SIDNAKTYNN NLIQNVKKGT PKRPGAYLET YILDMYDEDL KSSELEQHWG
321 LFTANGDLKY PVNFN

Supporting Peptides:

Peptide	Uniq	-10lgP	Mass	Length	ppm	m/z	z	RT	Fraction	Scan	Source File	Area AndrejP_JAtreatment	Area AndrejP_Feeding	Area AndrejP_CNTR	#Feature	#Feature AndrejP_JAtreatment	#Feature AndrejP_Feeding	#Feature AndrejP_CNTR	Start	End	PTM	AScore	Found By
R.YLAVGNEIRPN.L	Y	39.83	1244.6512	11	1.5	623.3322	2	18.74	13	F13:3557	MAX09856_AndrejP_gel_SA07_BA7_01_19060.mgf	0			0	0	0	0	107	117			PEAKS DB
total 1 peptides																							

YP_009944427.1

back to list

| **Protein Coverage** | **Supporting Peptides** |

Protein Coverage:

1 MRINPTTSGS GVSTLEKKNL GRIDQIIGPV LDVAFPPGKM PNIYNALVVE GRDTAGQPIN VTCEVQQLLG NNRVRAVAMS

o Oxidation (M) (+15.99)

81 ATDGLMRGME VVDTGSSLSV PVGGATLGRI FNVLGEPVDN LGPVDTRTTS PIHRSAPAFI QLDTKLSIFE TGIKVVDLLA

183 o

161 PYRRGGKIGL FGGAGVGK**TV LIMELINNIA K**AHGGVSVFG GVGERTREGN DLYMEMKESG VINEQNIAES KVALVYGQMN
241 EPPGARMRVG LTALTMAEYF RDVNEQDVLL FIDNIFRFVQ AGSEVSALLG RMPSAVGYQP TLSTEMGSLQ ERITSTKEGS
321 ITSIQAVYVP ADDLTDPAPA TTFAHLDATT VLSRGLAAKG IYPAVDPLDS TSTMLQPRIV GEEHYETAQR VKQTLQRYKE
401 LQDIIAILGL DELSEEDRLT VARARKIERF LSQPFFVAEV FTGSPGKYVG LTETIRGFQL ILSGELDGLP EQAFYLVGNI
481 DEATAKAMNF EMENKLKK

Supporting Peptides:

Peptide	Uniq	-10lgP	Mass	Length	ppm	m/z	z	RT	Fraction	Scan	Source File	Area AndrejP_JAtreatment	Area AndrejP_Feeding	Area AndrejP_CNTR	#Feature	#Feature AndrejP_JAtreatment	#Feature AndrejP_Feeding	#Feature AndrejP_CNTR	Start	End	PTM	AScore	Found By
K.TVLIM(+15.99)ELINNIAK.A	Y	38.94	1486.8429	13	3.5	744.4315	2	59.14	20	F20:13917	MAX09859_AndrejP_gel_SA10_BB2_01_19067.mgf	0			0	0	0	0	179	191	Oxidation (M)	M5:Oxidation (M):1000.00	PEAKS DB
total 1 peptides																							

XP_010685437.1

back to list

| **Protein Coverage** | **Supporting Peptides** |

Protein Coverage:

1 MINGKRESKE MESPNQEKPS LFNSMFKAKY DKITTDVSHI SQITKELAQD DPRRVVHSIK VAFAITVVSL FYYFDPLYEG

d Deamidation (NQ) (+0.98)

81 FGVGAMWAVL TVVVVFEFSV GATLGKGVNR VLATILGGML AVGAHRLSSL PGKKLEPFCL GVCVFITAGV TTFLRFFPKM
161 KARFDYGVLI FILTFSLICV SGYRDDEVID MAHKRITTIL IGSSTSVIVC VVICPVWAGT DLHKLVATNL LTLALFFQEF

279 d

241 GVEYFKTSSD EIANDNNEAL LDGLKNVLDS K**NNLDSLLNL AK**WEPRHGRF KGSHPWTQYQ KIGELARHCA CRAEALHGFI
321 YPHLQIPKEV KSKFRETRCK MSSESGIALK ELSLAMKKMT KPSKAKPHLI NAKQAAQNLN LLLKSNIWKN LNLSEVTSVA
401 TVATLLVDIV TCIQEIADAV EELASLAKFK DFKDVKVAPQ IEKAKLGNQG SIKRAFLC

Supporting Peptides:

Peptide	Uniq	-10lgP	Mass	Length	ppm	m/z	z	RT	Fraction	Scan	Source File	Area AndrejP_JAtreatment	Area AndrejP_Feeding	Area AndrejP_CNTR	#Feature	#Feature AndrejP_JAtreatment	#Feature AndrejP_Feeding	#Feature AndrejP_CNTR	Start	End	PTM	AScore	Found By
K.NNLDSLLN(+.98)LAK.W	Y	38.88	1214.6506	11	20.8	608.3414	2	57.74	48	F48:14847	MAX09909_AndrejP_gel_C02_RC2_01_19201.mgf	0	0	0	0	0	0	0	272	282	Deamidation (NQ)	N8:Deamidation (N Q):161.52	PEAKS DB
total 1 peptides																							

XP_021743893.1

back to list

| Protein Coverage | Supporting Peptides |

Protein Coverage:

1 MAGKYGAKIV KGTGGRGKHK SGIKKVSRST K**AGLQFPVGR** IGRFLKQGRY AQRLGTYSPV YLSAVLEYLA AEVLELAGNA

81 ARDNKKKRIK PRHIQLAVRS DEELNKLFGS VTIANGGVMP YIHGALLPMN TSKSESNNGK

Supporting Peptides:

Peptide	Uniq	-10lgP	Mass	Length	ppm	m/z	z	RT	Fraction	Scan	Source File	Area AndrejP_JAtreatment	Area AndrejP_Feeding	Area AndrejP_CNTR	#Feature	#Feature AndrejP_JAtreatment	#Feature AndrejP_Feeding	#Feature AndrejP_CNTR	Start	End	PTM	AScore	Found By
K.AGLQFPVGR.I	Y	37.18	943.5239	9	-1.5	472.7655	2	34.59	66	F66:7909	MAX09918_AndrejP_gel_C11_RD3_01_19219.mgf			0	0	0	0	0	32	40			PEAKS DB
total 1 peptides																							

AAQ75601.1

back to list

| Protein Coverage | Supporting Peptides |

Protein Coverage:

1 LTYYTPEYET LDTDILAAFR VTPQPGVPPE EAGAAVAAES STGTWTTVWT DGLTNLDRYK GRCYHIEPVA GEENQYICYV

C Carbamidomethylation (+57.02)

81 AYPLDLFEEG SVTNMFTSIV GNVFGFKALR ALRLEDLRIP VAYVKTFQGP PHGIQVERDK LNKYGRPLLG CTIKPKLGLS

161 AKNYGRACYE CLRGGLDFTK DDENVNSQPF MRWRDRFVFC AEALYKAQAE TGEIKGHYLN ATAGTCEEMI KRAVFARELG

241 VPIVMHDYLT GGFTANTSLS HYCRDNGLLL HIHRAMHAVI DRQKNHGMHF RVLAKALRLS GGDHIHSGTV VGKLEGERDI

321 TLGFVDLLRD DYTEKDRSRG IYFTQSWVST PGVIPVASGG IHVWHMPALT EIFGDDSVLQ FGGGTLGHPW GNAPGAVANR

406 C

401 **VALEACVQAR** NEGRDLAREG NEIIRAAAKW SPELAAACEI WNEIKFE

Supporting Peptides:

Peptide	Uniq	-10lgP	Mass	Length	ppm	m/z	z	RT	Fraction	Scan	Source File	Area AndrejP_JAtreatment	Area AndrejP_Feeding	Area AndrejP_CNTR	#Feature	#Feature AndrejP_JAtreatment	#Feature AndrejP_Feeding	#Feature AndrejP_CNTR	Start	End	PTM	AScore	Found By
R.VALEAC(+57.02)VQAR.N	Y	37.16	1115.5757	10	0.9	558.7923	2	15.34	4	F4:2455	MAX09851_AndrejP_gel_SA02_BA2_01_19051.mgf	0			0	0	0	0	401	410	Carbamidomethylation	C6:Carbamidomethylation:1000.00	PEAKS DB
total 1 peptides																							

XP_021774585.1

back to list

| Protein Coverage | Supporting Peptides |

Protein Coverage:

1 MKRELAALEM QSPVIGRTTR SSKRLSVGGS SPVSESLVYK RVKRSTVRAR KSEVLSNGGG DNGAVEISEA SEGVGDQNGA

81 HDPQPEEKIG VPEVQEREIV VAAGVDSEVV KSVCDEEVKN VYEVSSMAVE EKGVNESSVN GDGDGDGLMK NEVCDDVEVN

161 GELFEEQNLV AIKIDYQDAV VNEVHANGGS IEVEKVLEGI STKSPLNAVN GKAKKSSIER YPRRFTRSAL KSKVDTVKIG

241 NDYVEQGNSK AVTNEGNGEV CKEIVVYSEA SRSESNGVKK NSLLEKYSRR VTRSALKPKV DDSTLR**SVEP ATLDNK**KAVS

321 APLDTKMAVA VTLDIETLVP VKNSENAECD AGKDSEEPSS APPRRKLEMK MSKQISHGKV PSNVQELLAT GLLEGCPVYY

401 DGGKGLKLNG RIRGIGILCS CGLCKGCKVI PPSLFEIHAC NKYKRAVQYI YLENGRSLIH ILKACKNTRL STLEATIQNA

481 IGPLPEKKLI VCQNCKEPFF SMDAESSEPV CSKCVMLNLS SIGPVYSTRK RCRSSKFVFP SQSTSACDMA QDSSPKNSIE

561 NSGEAILTPK LQTSQADVPY GVLQGRSRAK LVKKSAGAFL TPKLRSSSRI GRISETKMSE EIVKNAQDSS MSPKTCISPV

641 VGKSAGTKDH KKLKRRSSKL ALSPKPSPGN ACAKVKTRLR EEVLTPKPSK TPTTLKSSEK KTSGKITRKD LRLHKLVFED

721 DVLPDGTVLG YYARGQKLLE GSKKGSGILC NCCDTVVSAS QFEAHAGCAS RRKPYCYIYT SNGVSLHELS VTLIKDRRHS

801 AKYNDDLCSI CADGGNLLLC DGCPRAFHTE CASLPSIPRG KWYCKYCQNM FEREKFVAHN ANALAAGRVS GVDSIEQITK

881 QSIRIVNNLA SEVSACILCR GFDFCKTGFG PRTIILCDQC EKEYHVGCLK DHNMADLTEL PDGKWFCSMD CRRVESSLQN

961 LLVRGVEKLP ESLVDIIRKK NMHIGSDSGS DLEVSWRLLS GKVASPETRP LLSQAVAIFH ESFAPIIDVV SGHDLIPAMV

1041 YGRNVGGQEY GGMYCAVLTA NKVVVSAGIF RIFGPDVAEL PLVATSSGNH GKGYFQTLFA CIERLLAFLK VKTIVLPAAE

1121 EAGSIWTDRF GFTKMTLDQL RELKRNFWSL VRFQGTSMLH KLVPDCRECG SLAE

Supporting Peptides:

Peptide	Uniq	-10lgP	Mass	Length	ppm	m/z	z	RT	Fraction	Scan	Source File	Area AndrejP_JAtreatment	Area AndrejP_Feeding	Area AndrejP_CNTR	#Feature	#Feature AndrejP_JAtreatment	#Feature AndrejP_Feeding	#Feature AndrejP_CNTR	Start	End	PTM	AScore	Found By
total 1 peptides																							

Peptide	Uniq	-10lgP	Mass	Length	ppm	m/z	z	RT	Fraction	Scan	Source File	Area AndrejP_JAtreatment	Area AndrejP_Feeding	Area AndrejP_CNTR	#Feature	#Feature AndrejP_JAtreatment	#Feature AndrejP_Feeding	#Feature AndrejP_CNTR	Start	End	PTM	AScore	Found By
R.SVEPATLDNK.K	Y	36.98	1072.5400	10	11.8	537.2822	2	18.70	13	F13:3485	MAX09856_AndrejP_gel_SA07_BA7_01_19060.mgf	0			0	0	0	0	307	316			PEAKS DB
total 1 peptides																							

XP_021761383.1

back to list

| **Protein Coverage** | **Supporting Peptides** |

Protein Coverage:

Oxidation (M) (+15.99)

```
  1  MGVKIWSSPL FLLVITLVSL ALKANAGGIA VYWGQNRNEG TLTDTCRSML FKYVNIAFLS KFGSGQKPEI DLAGHCIPNG
 81  GGCQRVSVGI RNCQSLGIKV MLSIGGGNGS YSLSSSEDAR GVADYLWDHF LGGQSQFRPL GNAVLDGIDF DIESGKPHYQ
161  ALARRLTEHG QQSGKKVYLS AAPQCPFPDQ KLNGALNTGL FDYVWIQFYN NPICEFDVSN PEKFKNSWRT WTSNIPAKKF
                                                   282
241  FVGLPAAATK SAAGSGFVPA QTLINQVLPF VKGVGEKYGG VMLWDRANDV RTKYSQRIKN SV
```

Supporting Peptides:

Peptide	Uniq	-10lgP	Mass	Length	ppm	m/z	z	RT	Fraction	Scan	Source File	Area AndrejP_JAtreatment	Area AndrejP_Feeding	Area AndrejP_CNTR	#Feature	#Feature AndrejP_JAtreatment	#Feature AndrejP_Feeding	#Feature AndrejP_CNTR	Start	End	PTM	AScore	Found By
K.YGGVM(+15.99)LWDR.A	Y	36.74	1111.5121	9	-5.0	556.7593	2	26.51	14	F14:5609	MAX09856_AndrejP_gel_SA07_BA7_01_19061.mgf	0	0	0	0	0	0	0	278	286	Oxidation (M)	M5:Oxidation (M):1000.00	PEAKS DB
K.YGGVMLWDR.A	Y	36.02	1095.5171	9	-5.0	548.7619	2	32.85	14	F14:7153	MAX09856_AndrejP_gel_SA07_BA7_01_19061.mgf	0			0	0	0	0	278	286			PEAKS DB
total 2 peptides																							

XP_010669985.1

back to list

| **Protein Coverage** | **Supporting Peptides** |

Protein Coverage:

A->G

```
  1  MATKRSVSTL QESDLKGKRV FVRVDLNVPL DDNCNITDDT RIRAAIPTIK YLSGYGAKVI LCSHLGRPKG VTPKFSLKPL
                                                                   142
 81  VPRLSELLGC EVKMAKDSIG EEVEKMVAEL PEGGVVLLEN VRFYKEEEKN DPEFAKKLAS LADVYVNDAF GTAHRAHAST
161  EGVANYLKPS VAGYLMQKEL DYLVGAVANP KKPFAAIVGG SKVSSKIGVI ESLLAKVDYL ILGGGMIFTF YKAQGISVGS
241  SLVEEDKLEL ATSLIEKAKA KGVSLMLPTD VVIADKFAAD AESKTVPASA IPDGWMGLDI GPDSIKTFNE ALDKTQTIIW
321  NGPMGVFEFD KFAAGTEAVA RKLAELSGKG VTTIIGGGDS VAAVEKVGLA DKMSHISTGG GASLELLEGK PLPGVLALDD
401  A
```

Supporting Peptides:

Peptide	Uniq	-10lgP	Mass	Length	ppm	m/z	z	RT	Fraction	Scan	Source File	Area AndrejP_JAtreatment	Area AndrejP_Feeding	Area AndrejP_CNTR	#Feature	#Feature AndrejP_JAtreatment	#Feature AndrejP_Feeding	#Feature AndrejP_CNTR	Start	End	PTM	AScore	Found By
S.LG(sub A)DVYVNDAFGTAHR.A	Y	36.74	1633.7848	15	1.3	545.6006	3	35.34	56	F56:7915	MAX09913_AndrejP_gel_C06_RC6_01_19209.mgf			0	0	0	0	0	141	155			SPIDER
total 1 peptides																							

XP_021838555.1

back to list

| **Protein Coverage** | **Supporting Peptides** |

Protein Coverage:

```
  1  MVKKFAVQGK VWLGIMHFLP VLVRFLELDE LSLVNSGFKN LDVSSSDGIK ENVKSKVYQE RNLSQKLGVG SAVRQKTQSL
 81  MKTKVPSPQK INTGRLETIS RSAVAKSQSI TTSSKECLKS TGIRGYNKKP PLRAISRNGL GRVVSQQSDS RRSYPVSSSQ
161  SMLPSYATNM SVDFSPCKTS VTNLQTARVL KSQDKRGGSF TNDINASEEF NLKDQRKNHR HNSVTKQSLE QDASRQNNGT
241  TETKRSPFSS KTSSLGMPSQ KIGFFDMTSE KNRHDQDGKS NKVQRRTSQP SFKKQTNSLE FEEKENIFHP DDTVIELEPE
321  LNKGSSPSPS ESAAKGSSNA VQCSSTSKKS LTKAQRPAKQ ALFRTRTTSL TKVSPLKKSA FF
```

Supporting Peptides:

Peptide	Uniq	-10lgP	Mass	Length	ppm	m/z	z	RT	Fraction	Scan	Source File	Area AndrejP_JAtreatment	Area AndrejP_Feeding	Area AndrejP_CNTR	#Feature	#Feature AndrejP_JAtreatment	#Feature AndrejP_Feeding	#Feature AndrejP_CNTR	Start	End	PTM	AScore	Found By
R.HN(+.98)SVTKQSLEQDASR.Q	Y	36.16	1699.8125	15	-5.2	850.9059	2	51.79	44	F44:12906	MAX09907_AndrejP_gel_F11_RB3_01_19194.mgf		0		0	0	0	0	221	235		N2:Deamidation (N Q):56.99	PEAKS DB
total 1 peptides																							

KAH9622019.1

back to list

| **Protein Coverage** | **Supporting Peptides** |

Protein Coverage:

```
  1  MSKRKTREPK EETVTLGPAV RDGEHVFGVA HIFASFNDTF IHVTDLSGRE TLVRITGGMK VKADRDESSP YAAMLAAQDV
 81  SQRCKELGIT ALHIKLRATG GNKTKTPGPG AQSALRALAR SGMKIGRIED VTPIPTDSTR RKGGRRGRRL
```

Supporting Peptides:

Peptide	Uniq	-10lgP	Mass	Length	ppm	m/z	z	RT	Fraction	Scan	Source File	Area AndrejP_JAtreatment	Area AndrejP_Feeding	Area AndrejP_CNTR	#Feature	#Feature AndrejP_JAtreatment	#Feature AndrejP_Feeding	#Feature AndrejP_CNTR	Start	End	PTM	AScore	Found By
K.TPGPGAQSALR.A	Y	35.74	1053.5566	11	-3.3	527.7803	2	13.82	65	F65:2304	MAX09918_AndrejP_gel_C11_RD3_01_19218.mgf			0	0	0	0	0	106	116			PEAKS DB
total 1 peptides																							

XP_010676513.2

back to list

| Protein Coverage | Supporting Peptides |

Protein Coverage:

a Acetylation (Protein N-term) (+42.01)

1 MSHLKPLSIL FCLLLVITSL VFTPSNAITF TVKNNCPYTV WGAAVPGGGQ QMNSGSTWTV TANPGQQGAR IWARTGCTVT

81 GPNGLRCNTG DCGGLLQCTA YGTPPNTLAE YGLKQFNNLD FFDISLVDGF NVPMSFLPTG GCSRGITCSS DLNGPCPGPL

196 a

161 RVNGGCNNPC TVFKTDNYCC NSGSCGPTDY SRYFK**GKCPD AYSYPK**DDAT STFTCPTGTN YMVTFCP

Supporting Peptides:

Peptide	Uniq	-10lgP	Mass	Length	ppm	m/z	z	RT	Fraction	Scan	Source File	Area AndrejP_JAtreatment	Area AndrejP_Feeding	Area AndrejP_CNTR	#Feature	#Feature AndrejP_JAtreatment	#Feature AndrejP_Feeding	#Feature AndrejP_CNTR	Start	End	PTM	AScore	Found By
K.G(+42.01)KCPDAYSYPK.D	Y	35.12	1269.5699	11	1.5	635.7901	2	25.79	54	F54:6100	MAX09912_AndrejP_gel_C05_RC5_01_19207.mgf			0	0	0	0	0	196	206	Acetylation (Protein N-term)	G1:Acetylation (Protein N-term):1000.00	SPIDER
total 1 peptides																							

XP_021861922.1

back to list

| Protein Coverage | Supporting Peptides |

Protein Coverage:

1 MAIPDNLTRD QYVYMAKLAE QAERYEEMAK FMDKLVVTTT SSGGELTVEE RNLLSVAYKN VIGSLRAAWR IVSSIEQKEE

81 GRKNDDHVVL VRDYRTKVES ELSDICACIL KILDSNLIPT ASVSESRVFY LKMKGDYHRY LAEFKSGEER KSAAEDTMEA

161 YKNAQEIAVT DLPPTHPIRL GLALNFSVFY YEILNAADKA CSMAKQAFEE AIAELDTLGE ESYK**DSTLIM QLLR**DNLTLW

241 TSDQDQLDES

Supporting Peptides:

Peptide	Uniq	-10lgP	Mass	Length	ppm	m/z	z	RT	Fraction	Scan	Source File	Area AndrejP_JAtreatment	Area AndrejP_Feeding	Area AndrejP_CNTR	#Feature	#Feature AndrejP_JAtreatment	#Feature AndrejP_Feeding	#Feature AndrejP_CNTR	Start	End	PTM	AScore	Found By
K.DSTLIMQLLR.D	Y	35.09	1188.6537	10	-1.4	595.3302	2	58.09	24	F24:14454	MAX09897_AndrejP_gel_F01_RA1_01_19174.mgf		0		0	0	0	0	225	234			PEAKS DB
total 1 peptides																							

XP_010690445.1

back to list

| Protein Coverage | Supporting Peptides |

Protein Coverage:

1 MAVVSTSLAS QMTNPNSVVS TQFSGLRRSF FKLETSVSTQ SLFQNVESHL RLSSTRRGSR GVVAMAASGK **FFVGGNWK**CN

81 GTKESITKLV SDLNSATLEA DVDIVVAPPF VYIDQVKSSL TNRVEISAQN CWIGKGGAFT GEISAEQVKD LGCQWVILGH

161 SERRHVIGEK DEFIGKKAAY ALNQGLGVIA CIGELLEERE AGKTFDVCYQ QLKAFADALP SWENVVIAYE PVWAIGTGKV

241 ASPDQAQEVH VAVRDWLKKN VSEEVASKTR IIYGGSVNGG NCAELAKKED IDGFLVGGAS LKGPEFATIV NSVTAKKVAA

Supporting Peptides:

Peptide	Uniq	-10lgP	Mass	Length	ppm	m/z	z	RT	Fraction	Scan	Source File	Area AndrejP_JAtreatment	Area AndrejP_Feeding	Area AndrejP_CNTR	#Feature	#Feature AndrejP_JAtreatment	#Feature AndrejP_Feeding	#Feature AndrejP_CNTR	Start	End	PTM	AScore	Found By
K.FFVGGNWK.C	Y	34.95	953.4759	8	0.9	477.7436	2	40.74	55	F55:9270	MAX09913_AndrejP_gel_C06_RC6_01_19208.mgf			0	0	0	0	0	71	78			PEAKS DB
total 1 peptides																							

Peptide List

Prepared with PEAKS ™ (bioinfor.com)
